# Supplementary material for: Fossil-calibrated molecular clock data enable reconstruction of steps leading to differentiated multicellularity and anisogamy in the Volvocine algae
Source: BMC Biol. 2024 Apr 10;22:79. doi: 10.1186/s12915-024-01878-1 (PMC11007952; doi:10.1186/s12915-024-01878-1)
Supplement: Supplementary file 2 — Additional File 2: Tables S1-S7. [file 12915_2024_1878_MOESM2_ESM.pdf]

Additional File 2: Supplementary Tables

Table S1. Strain information for sampled taxa.

Division	Species	Source	Accession ID or DOI	Type
Rhodophyta	*Cyanidioschyzon merolae*	Ensembl	GCA_000091205.1	Proteome
Rhodophyta	*Galdieria sulphuraria*	Ensembl	GCA_000341285.1	Proteome
Rhodophyta	*Cyanidiococcus yangmingshanensis*	NCBI	ASM1399567v1	Proteome
Rhodophyta	*Porphyra umbilicalis*	JGI	MXAK01000000	Genome
Rhodophyta	*Pyropia yezoensis*	1kp	ERS1830191	Transcriptome
Rhodophyta	*Chondrus crispus*	Ensembl	GCA_000350225.2	Proteome
Rhodophyta	*Ahnfeltiopsis flabelliformis*	1kp	ERS1830200	Transcriptome
Rhodophyta	*Gloiopeltis furcata*	1kp	ERS1830197	Transcriptome
Rhodophyta	*Mazzaella japonica*	1kp	ERS1830199	Transcriptome
Rhodophyta	*Grateloupia livida*	1kp	ERS1830209	Transcriptome
Rhodophyta	*Grateloupia catenata*	1kp	ERS1830211	Transcriptome
Rhodophyta	*Grateloupia turuturu*	1kp	ERS1830210	Transcriptome
Streptophyta	*Klebsormidium nitens*	Tokyo Institute of Technology	https://doi.org/10.1038/ncomms4978	Proteome
Streptophyta	*Nitella mirabilis*	NCBI	PRJNA158153	Transcriptome
Streptophyta	*Chara braunii*	Ensembl	GCA_003427395.1	Proteome
Streptophyta	*Chara globularis*	Vries et al. (2018)	SRS2443003	Transcriptome
Streptophyta	*Coleochaete orbicularis*	NCBI	SRS10979585	Transcriptome
Streptophyta	*Coleochaete scutata*	Vries et al. (2018)	SRS2443011	Transcriptome
Streptophyta	*Staurastrum sebaldi*	1kp	ERS1830172	Transcriptome
Streptophyta	*Cosmarium granatum*	1kp	ERS1830163	Transcriptome
Streptophyta	*Euastrum affine*	1kp	ERS1830167	Transcriptome
Streptophyta	*Cylindrocystis cushleckae*	1kp	ERS368241	Transcriptome
Streptophyta	*Mesotaenium kramstae*	1kp	ERS1830178	Transcriptome
Streptophyta	*Zygnemopsis sp.*	1kp	ERS3670390	Transcriptome
Streptophyta	*Radula lindenbergiana*	1kp	ERS1830058	Transcriptome
Streptophyta	*Frullania sp.*	1kp	ERS3670367	Transcriptome
Streptophyta	*Scapania nemorea*	1kp	ERS1830046	Transcriptome
Streptophyta	*Barbilophozia barbata*	1kp	ERS1830047	Transcriptome
Streptophyta	*Marchantia polymorpha*	JGI	PNPG01000000	Genome
Streptophyta	*Selaginella moellendorffii*	JGI	https://doi.org/10.1126/science.1203810	Genome

Streptophyta	*Isoetes tegetiformans*	1kp	ERS1829935	Transcriptome
Streptophyta	*Phylloglossum drummondii*	1kp	ERS1829929	Transcriptome
Streptophyta	*Huperzia lucidula*	1kp	ERS1829926	Transcriptome
Streptophyta	*Lycopodium annotinum*	1kp	ERS1829933	Transcriptome
Streptophyta	*Polystichum tripteron*	Qi et al. (2018)	SRR6920645	Transcriptome
Streptophyta	*Cyrtomium fortunei*	Qi et al. (2018)	SRR6920659	Transcriptome
Streptophyta	*Dryopteris decipiens*	Qi et al. (2018)	SRR6920660	Transcriptome
Streptophyta	*Ctenitis subglandulosa*	Qi et al. (2018)	SRR6920661	Transcriptome
Streptophyta	*Thuja plicata*	JGI	https://doi.org/10.1101/gr.276358.121	Genome
Streptophyta	*Pinus hwangshanensis*	Jin et al. (2021)	SRR13823644	Transcriptome
Streptophyta	*Ginkgo biloba*	1kp	ERS368269	Transcriptome
Streptophyta	*Amborella trichopoda*	JGI	https://doi.org/10.1126/science.1241089	Genome
Streptophyta	*Nymphaea colorata*	JGI	https://doi.org/10.1038/s41586-019-1852-5	Genome
Streptophyta	*Nymphaea caerulea*	Zhang et al. (2019)	SRR10158664	Transcriptome
Streptophyta	*Cinnamomum kanehirae*	JGI	https://doi.org/10.1038/s41477-018-0337-0	Genome
Streptophyta	*Peumus boldus*	1kp	ERS1829196	Transcriptome
Streptophyta	*Gyrocarpus americanus*	1kp	ERS1829190	Transcriptome
Streptophyta	*Sarcandra glabra*	1kp	ERS368201	Transcriptome
Streptophyta	*Ascarina rubricaulis*	1kp	ERS1829208	Transcriptome
Streptophyta	*Arabidopsis thaliana*	JGI	https://doi.org/10.1093/nar/gkr1090	Genome
Streptophyta	*Capsella rubella*	JGI	https://doi.org/10.1038/ng.2669	Genome
Streptophyta	*Eutrema salsugineum*	JGI	https://doi.org/10.3389/fpls.2013.00046	Genome
Streptophyta	*Brassica rapa*	JGI	QMKI01000000	Genome
Streptophyta	*Brachypodium distachyon*	JGI	ADDN02000000	Genome
Streptophyta	*Oryza sativa*	JGI	https://doi.org/10.1093/nar/gkl976	Genome
Streptophyta	*Sorghum bicolor*	JGI	ABXC03000000	Genome
Streptophyta	*Zea Mays*	JGI	GCA_000005015.6	Genome
Chlorophyta	*Bathycoccus prasinos*	NCBI	ASM222023v1	Proteome
Chlorophyta	*Ostreococcus tauri*	NCBI	GCF_000214015.3	Genome
Chlorophyta	*Micromonas pusilla*	JGI	https://doi.org/10.1126/science.1167222	Proteome
Chlorophyta	*Tetraselmis cordiformis*	1kp	ERS1830070	Transcriptome
Chlorophyta	*Tetraselmis striata*	NCBI	SRS10979578	Transcriptome
Chlorophyta	*Coccomyxa subellipsoidea*	JGI	https://doi.org/10.1186/gb-2012-13-5-r39	Genome

Chlorophyta	*Botryococcus braunii*	1kp	ERS1830127	Transcriptome
Chlorophyta	*Botryococcus terribilis*	1kp	ERS1830129	Transcriptome
Chlorophyta	*Chlorella variabilis*	NCBI	GCF_000147415.1	Proteome
Chlorophyta	*Ulva mutabilis*	OrcAE; De Clerck et al. (2018)	https://doi.org/10.1016/j.cub.2018.08.015	Proteome
Chlorophyta	*Percursaria percursa*	1kp	ERS1830140	Transcriptome
Chlorophyta	*Ochlochaete sp.*	1kp	ERS3670385	Transcriptome
Chlorophyta	*Entocladia endozoica*	1kp	ERS1830144	Transcriptome
Chlorophyta	*Acrosiphonia sp.*	1kp	ERS3670386	Transcriptome
Chlorophyta	*Helicodictyon planctonicum*	1kp	ERS1830074	Transcriptome
Chlorophyta	*Hazenia basiliensis*	Hou et al. (2022)	SRS8850118	Transcriptome
Chlorophyta	*Rhexinema paucicellulare*	Hou et al. (2022)	SRS8850117	Transcriptome
Chlorophyta	*Planophila laetevirens*	1kp	ERS1830148	Transcriptome
Chlorophyta	*Planophila sp*	1kp	ERS1830149	Transcriptome
Chlorophyta	*Halochlorococcum marinum*	1kp	ERS1830151	Transcriptome
Chlorophyta	*Oltmannsiellopsis unicellularis*	NCBI-SRS10979569	SRS10979569	Transcriptome
Chlorophyta	*Oltmannsiellopsis viridis*	1kp	ERS1830142,ERS1830143	Transcriptome
Chlorophyta	*Ignatius tetrasporus*	1kp	ERS1830151	Transcriptome
Chlorophyta	*Caulerpa lentillifera*	Arimoto et al. (2019)	10.1111/dgd.12634	Transcriptome
Chlorophyta	*Caulerpa taxifolia*	Ranjan et al. (2015)	https://doi.org/10.1371/journal.pgen.1004900	Transcriptome
Chlorophyta	*Caulerpa cylindracea*	Unlu et al. (2019)	SRR7168057	Transcriptome
Chlorophyta	*Codium fragile*	1kp	ERS1830154	Transcriptome
Chlorophyta	*Bryopsis plumosa*	1kp	ERS1830153	Transcriptome
Chlorophyta	*Bryopsis hypnoides*	Hou et al. (2022)	SRS8850115	Transcriptome
Chlorophyta	*Ostreobium quekettii*	Hou et al. (2022)	SRS8850114	Transcriptome
Chlorophyta	*Oedogonium cardiacum*	1kp	ERS1830083	Transcriptome
Chlorophyta	*Oedogonium foveolatum*	1kp	ERS1830084	Transcriptome
Chlorophyta	*Chaetopeltis orbicularis*	1kp	ERS1830072	Transcriptome
Chlorophyta	*Stigeoclonium helveticum*	1kp	ERS1830076	Transcriptome
Chlorophyta	*Aphanochaete repens*	1kp	ERS1830078	Transcriptome
Chlorophyta	*Scenedesmus glucoliberatum PABB004*	Mancipe et al (2021)	https://doi.org/10.1111/jam.15311	Transcriptome

Chlorophyta	*Pediastrum duplex*	1kp	ERS3670374	Transcriptome
Chlorophyta	*Chromochloris zofingiensis*	JGI	https://doi.org/10.1073/pnas.1619928114	Genome
Chlorophyta	*Golenkinia longispicula*	1kp	ERS1830080	Transcriptome
Chlorophyta	*Astrephomene gubernaculifera - NIES-418*	Lindsey et al. (2021)	SAMN17884640	Transcriptome
Chlorophyta	*Astrephomene perforate NIES-564*	Lindsey et al. (2021)	SAMN17884616	Transcriptome
Chlorophyta	*Basichlamys sacculifera NIES-566*	Lindsey et al. (2021)	SAMN17884617	Transcriptome
Chlorophyta	*Colemanosphaera charkowiensis NIES-3383*	Lindsey et al. (2021)	SAMN17884629	Transcriptome
Chlorophyta	*Chlamydomonas debaryana SAG 11-55a*	Lindsey et al. (2021)	SAMN17884664	Transcriptome
Chlorophyta	*Chlamydomonas debaryana SAG 70.81*	Lindsey et al. (2021)	SAMN17884633	Transcriptome
Chlorophyta	*Chlamydomonas globosa SAG 81.72*	Lindsey et al. (2021)	SAMN17884666	Transcriptome
Chlorophyta	*Chlamydomonas moewusii SAG 11-16f*	Lindsey et al. (2021)	SAMN17884663	Transcriptome
Chlorophyta	*Chlamydomonas schloesseri*	Lindsey et al. (2021)	SAMN17884667	Transcriptome
Chlorophyta	*Eudorina cylindrica NIES-722*	Lindsey et al. (2021)	SAMN17884650	Transcriptome
Chlorophyta	*Eudorina elegans NIES-456*	Lindsey et al. (2021)	SAMN17884613	Transcriptome
Chlorophyta	*Eudorina elegans NIES-458*	Lindsey et al. (2021)	SAMN17884614	Transcriptome
Chlorophyta	*Eudorina elegans NIES-568*	Lindsey et al. (2021)	SAMN17884644	Transcriptome
Chlorophyta	*Eudorina elegans NIES-717*	Lindsey et al. (2021)	SAMN17884647	Transcriptome
Chlorophyta	*Eudorina elegans NIES-719*	Lindsey et al. (2021)	SAMN17884648	Transcriptome
Chlorophyta	*Eudorina elegans NIES-720*	Lindsey et al. (2021)	SAMN17884649	Transcriptome
Chlorophyta	*Eudorina illinoisensis NIES-460*	Lindsey et al. (2021)	SAMN17884641	Transcriptome
Chlorophyta	*Eudorina minodii NIES-856*	Lindsey et al. (2021)	SAMN17884655	Transcriptome
Chlorophyta	*Eudorina peripheralis NIES-725*	Lindsey et al. (2021)	SAMN17884651	Transcriptome
Chlorophyta	*Eudorina unicocca SAG 24-1c*	Lindsey et al. (2021)	SAMN17884665	Transcriptome

Chlorophyta	*Gonium multicoccum NIES-737*	Lindsey et al. (2021)	SAMN17884621	Transcriptome
Chlorophyta	*Gonium octonarium NIES-851*	Lindsey et al. (2021)	SAMN17884622	Transcriptome
Chlorophyta	*Gonium quadratum NIES-653*	Lindsey et al. (2021)	SAMN17884646	Transcriptome
Chlorophyta	*Gonium viridistellatum NIES-654*	Lindsey et al. (2021)	SAMN17884619	Transcriptome
Chlorophyta	*Pandorina colemaniae NIES-572*	Lindsey et al. (2021)	SAMN17884618	Transcriptome
Chlorophyta	*Pandorina morum NIES-890*	Lindsey et al. (2021)	SAMN17884659	Transcriptome
Chlorophyta	*Platydorina caudata NIES-728*	Lindsey et al. (2021)	SAMN17884652	Transcriptome
Chlorophyta	*Pleodorina indica NIES-736*	Lindsey et al. (2021)	SAMN17884654	Transcriptome
Chlorophyta	*Pleodorina japonica UTEX 2523*	Lindsey et al. (2021)	SAMN17884639	Transcriptome
Chlorophyta	*Pleodorina starrii NIES-1362*	Lindsey et al. (2021)	SAMN17884626	Transcriptome
Chlorophyta	*Pleodorina starrii NIES-1363*	Lindsey et al. (2021)	SAMN17884627	Transcriptome
Chlorophyta	*Pleodorina thompsonii NIES-4126*	Lindsey et al. (2021)	SAMN17884631	Transcriptome
Chlorophyta	*Vitreochlamys aulata NIES-878*	Lindsey et al. (2021)	SAMN17884623	Transcriptome
Chlorophyta	*Vitreochlamys aulata SAG 80.81*	Lindsey et al. (2021)	SAMN17884634	Transcriptome
Chlorophyta	*Vitreochlamys nekrassovii SAG 11-10*	Lindsey et al. (2021)	SAMN17884662	Transcriptome
Chlorophyta	*Vitreochlamys ordinate NIES-882*	Lindsey et al. (2021)	SAMN17884624	Transcriptome
Chlorophyta	*Volvox africanus NIES-863*	Lindsey et al. (2021)	SAMN17884637	Transcriptome
Chlorophyta	*Vitreochlamys aureus NIES-541*	Lindsey et al. (2021)	SAMN17884642	Transcriptome
Chlorophyta	*Vitreochlamys barberi NIES-730*	Lindsey et al. (2021)	SAMN17884653	Transcriptome
Chlorophyta	*Volvox carteri f. kawasakiensis NIES-732*	Lindsey et al. (2021)	SAMN17884620	Transcriptome
Chlorophyta	*Volvox carteri f. nagariensis NIES-865*	Lindsey et al. (2021)	SAMN17884656	Transcriptome
Chlorophyta	*Volvox carteri f. weismannia NIES-866*	Lindsey et al. (2021)	SAMN17884657	Transcriptome

Chlorophyta	*Volvox dissipatrix NIES-4128*	Lindsey et al. (2021)	SAMN17884661	Transcriptome
Chlorophyta	*Volvox ferrisii NIES-3986*	Lindsey et al. (2021)	SAMN17884630	Transcriptome
Chlorophyta	*Volvox gigas NIES-867*	Lindsey et al. (2021)	SAMN17884658	Transcriptome
Chlorophyta	*Volvox globator SAG 199.80*	Lindsey et al. (2021)	SAMN17884636	Transcriptome
Chlorophyta	*Volvox kirkiorum NIES-543*	Lindsey et al. (2021)	SAMN17884643	Transcriptome
Chlorophyta	*Volvox obversus NIES-868*	Lindsey et al. (2021)	SAMN17884638	Transcriptome
Chlorophyta	*Volvox ovalis NIES-2569*	Lindsey et al. (2021)	SAMN17884628	Transcriptome
Chlorophyta	*Volvox powersii NIES-4127*	Lindsey et al. (2021)	SAMN17884632	Transcriptome
Chlorophyta	*Volvox tertius NIES-544*	Lindsey et al. (2021)	SAMN17884615	Transcriptome
Chlorophyta	*Volvulina boldii NIES-893*	Lindsey et al. (2021)	SAMN17884660	Transcriptome
Chlorophyta	*Volvulina compacta NIES-582*	Lindsey et al. (2021)	SAMN17884645	Transcriptome
Chlorophyta	*Volvulina pringsheimii NIES-895*	Lindsey et al. (2021)	SAMN17884625	Transcriptome
Chlorophyta	*Volvulina steinii SAG 90-1*	Lindsey et al. (2021)	SAMN17884635	Transcriptome
Chlorophyta	*Colemanosphaera angeleri FACHB 2363*	Hu et al. (2020)	SRX5666821	Transcriptome
Chlorophyta	*Colemanosphaera charkowiensis FACHB 2326*	Hu et al. (2020)	SRX5666822	Transcriptome
Chlorophyta	*Eudorina cylindrica FACHB 2322*	Hu et al. (2020)	SRX5666825	Transcriptome
Chlorophyta	*Eudorina elegans FACHB 2321*	Hu et al. (2020)	SRX5666826	Transcriptome
Chlorophyta	*Pandorina colemaniae FACHB 2361*	Hu et al. (2020)	SRX5666819	Transcriptome
Chlorophyta	*Pandorina morum FACHB 2362*	Hu et al. (2020)	SRX5666823	Transcriptome
Chlorophyta	*Tetrabaena socialis NIES-571*	Featherston et al. (2018)	SRX3367144	Transcriptome
Chlorophyta	*Tetrabaena socialis NIES-691*	Zhang et al. (2019)	CRR044965	Transcriptome
Chlorophyta	*Volvulina compacta FACHB 2337*	Hu et al. (2020)	SRX5666820	Transcriptome
Chlorophyta	*Yamagishiella unicocca FACHB 2364*	Hu et al. (2020)	SRX5666824	Transcriptome

Chlorophyta	*Chlamydomonas reinhardtii*	JGI	https://doi.org/10.1126/science.1143609	Genome
Chlorophyta	*Gonium pectorale*	NCBI	GCA_001584585.1	Proteome
Chlorophyta	*Volvox carteri f. nagariensis*	JGI	https://doi.org/10.1126/science.1188800	Genome

Table S2. Fossil cross validation results

	Normal run					Fossil cross validation run			
	**Node of common ancestor**	**Mn. date**	**Lower 95% HPDI**	**Upper 95% HPDI**	**Fossil age calibration (MY)**	**Node of common ancestor**	**Mn. date**	**Lower 95% HPDI**	**Upper 95% HPDI**
**8 genes, CB**									
**CIR**	Cyamer & Chocri	1108.77	989.85	1319.82	MIN: 1047	Cyamer & Chocri	952.754	877.105	1058.41
	Graliv & Chocri	636.282	559.753	722.057	MIN: 609	Graliv & Chocri	566.615	361.991	706.595
	Euaaff & Zygsp	556.882	421.832	668.861	MIN: 350	Euaaff & Zygsp	537.815	397.252	667.063
	Marpol & Zeamay	467.31	449.339	493.85	MIN:480	Marpol & Zeamay	462.543	447.377	486.529
	Isoteg & Zeamay	421.59	419.16	423.083	423-419	Isoteg & Zeamay	620.792	544.026	704.688
	Ctesub & Zeamay	406.314	400.308	411.428	MIN: 385	Ctesub & Zeamay	406.549	400.621	412.06
	Ginbil & Zeamay	324.392	322.713	328.349	330-323	Ginbil & Zeamay	202.955	184.757	225.667
	Ambtri & Zeamay	128.213	125.996	129.166	129-125	Ambtri & Zeamay	260.388	244.092	275.015
	Sargla & Zeamay	105.277	99.0281	110.928	MIN: 125	Sargla & Zeamay	103.385	96.4085	109.64
	Aratha & Zeamay	89.27	81.9304	96.1691	MIN: 113	Aratha & Zeamay	86.1042	78.6394	93.5776
	Botbra & Botter	356.992	356.001	358	358-356	Botbra & Botter	300.992	205.501	392.715
	Bryhyp & Volcar	978.819	945.56	1042.22	1056-948	Bryhyp & Volcar	969.85	944.037	1029.52
	Codfra & Bryhyp	561.466	497.171	645.889	MIN: 541	Codfra & Bryhyp	460.462	318.694	627.277
	Acrsp & Plasp	464.043	457.97	470.033	470-458	Acrsp & Plasp	402.202	254.784	611.382
**LN**	Cyamer & Chocri	1159.85	994.913	1425	MIN: 1047	Cyamer & Chocri	991.513	858.215	1157.61
	Graliv & Chocri	660.431	544.313	782.352	MIN: 609	Graliv & Chocri	518.605	340.987	720.116
	Euaaff & Zygsp	602.242	501.799	721.559	MIN: 350	Euaaff & Zygsp	600.235	474.043	723.097
	Marpol & Zeamay	483.562	461.767	515.668	MIN:480	Marpol & Zeamay	477.555	457.249	507.995
	Isoteg & Zeamay	421.665	419.23	423.107	423-419	Isoteg & Zeamay	613.705	552.097	684.982
	Ctesub & Zeamay	403.404	397.384	409.095	MIN: 385	Ctesub & Zeamay	403.803	397.532	409.281
	Ginbil & Zeamay	324.231	322.677	328.012	330-323	Ginbil & Zeamay	203.845	185.967	224.696
	Ambtri & Zeamay	128.138	125.78	129.161	129-125	Ambtri & Zeamay	252.535	232.63	269.552
	Sargla & Zeamay	101.396	94.3616	107.919	MIN: 125	Sargla & Zeamay	98.4792	90.5993	105.985
	Aratha & Zeamay	83.3457	75.0767	91.1795	MIN: 113	Aratha & Zeamay	78.1526	68.8216	87.3476
	Botbra & Botter	357.003	356.001	358.001	358-356	Botbra & Botter	411.692	250.787	547.675
	Bryhyp & Volcar	981.857	945.324	1046.58	1056-948	Bryhyp & Volcar	977.473	945.168	1041.7
	Codfra & Bryhyp	566.887	508.585	657.879	MIN: 541	Codfra & Bryhyp	511.633	334.441	642.898
	Acrsp & Plasp	463.773	457.972	469.997	470-458	Acrsp & Plasp	390.511	279.518	533.471
**UGAM**	Cyamer & Chocri	1237.17	1043.86	1490.83	MIN: 1047	Cyamer & Chocri	993.897	849.154	1164.64
	Graliv & Chocri	648.391	531.747	804.893	MIN: 609	Graliv & Chocri	322.361	202.647	514.77
	Euaaff & Zygsp	498.656	352.225	688.264	MIN: 350	Euaaff & Zygsp	465.749	297.654	650.916
	Marpol & Zeamay	501.173	457.999	576.022	MIN:480	Marpol & Zeamay	487.069	449.414	560.295
	Isoteg & Zeamay	421.143	419.029	423.033	423-419	Isoteg & Zeamay	482.641	422.788	572.841
	Ctesub & Zeamay	391.489	375.648	405.254	MIN: 385	Ctesub & Zeamay	391.19	372.052	405.675
	Ginbil & Zeamay	325.909	322.914	329.878	330-323	Ginbil & Zeamay	231.066	181.507	296.479
	Ambtri & Zeamay	128.259	126.068	129.181	129-125	Ambtri & Zeamay	265.96	244.209	285.055

	Sargla & Zeamay	113.509	107.896	118.308	MIN: 125	Sargla & Zeamay	111.607	104.658	117.129
	Aratha & Zeamay	97.4493	89.8008	104.404	MIN: 113	Aratha & Zeamay	93.9419	84.8295	102.225
	Botbra & Botter	356.989	355.995	357.996	358-356	Botbra & Botter	175.941	85.5151	360.975
	Bryhyp & Volcar	968.636	942.785	1027.57	1056-948	Bryhyp & Volcar	965.219	942.588	1016.44
	Codfra & Bryhyp	564.364	478.222	683.923	MIN: 541	Codfra & Bryhyp	315.054	179.061	510.523
	Acrsp & Plasp	463.609	457.973	469.92	470-458	Acrsp & Plasp	326.935	230.326	431.51
**WN**	Cyamer & Chocri	1519.95	1305.91	1756.44	MIN: 1047	Cyamer & Chocri	1515.57	1150.07	1779.78
	Graliv & Chocri	587.438	497.992	672.502	MIN: 609	Graliv & Chocri	428.532	316.374	559.394
	Euaaff & Zygsp	571.745	471.764	675.812	MIN: 350	Euaaff & Zygsp	560.897	466.606	663.797
	Marpol & Zeamay	523.979	467.307	596.594	MIN:480	Marpol & Zeamay	518.69	449.306	595.411
	Isoteg & Zeamay	421.132	419.031	423.013	423-419	Isoteg & Zeamay	571.63	479.609	662.558
	Ctesub & Zeamay	394.933	369.891	415.822	MIN: 385	Ctesub & Zeamay	392.301	354.173	415.823
	Ginbil & Zeamay	326.106	322.952	329.939	330-323	Ginbil & Zeamay	261.624	199.439	327.518
	Ambtri & Zeamay	127.92	125.448	129.127	129-125	Ambtri & Zeamay	293.587	259.472	320.594
	Sargla & Zeamay	122.357	115.785	126.965	MIN: 125	Sargla & Zeamay	120.269	112.428	125.979
	Aratha & Zeamay	113.528	103.437	121.982	MIN: 113	Aratha & Zeamay	109.566	92.6081	121.597
	Botbra & Botter	356.985	355.993	358	358-356	Botbra & Botter	272.455	185.99	369.104
	Bryhyp & Volcar	976.837	944.744	1040.37	1056-948	Bryhyp & Volcar	972.551	944.721	1032.43
	Codfra & Bryhyp	540.86	463.239	616.331	MIN: 541	Codfra & Bryhyp	425.322	313.673	573.426
	Acrsp & Plasp	463.977	457.977	469.991	470-458	Acrsp & Plasp	456.047	357.675	564.529
**8 genes, ML**									
**CIR**	Cyamer & Chocri	1122.39	941.284	1348.46	MIN: 1047	Cyamer & Chocri	968.561	821.927	1167.21
	Graliv & Chocri	631.749	559.069	715.531	MIN: 609	Graliv & Chocri	530.194	347.899	688.558
	Euaaff & Zygsp	518.025	377.963	627.084	MIN: 350	Euaaff & Zygsp	494.368	371.262	616.002
	Marpol & Zeamay	464.312	445.528	492.542	MIN: 480	Marpol & Zeamay	458.16	443.314	480.746
	Isoteg & Zeamay	421.596	419.169	423.082	423-419	Isoteg & Zeamay	612.538	542.169	691.643
	Ctesub & Zeamay	403.201	396.649	409.008	MIN: 385	Ctesub & Zeamay	403.55	397.461	408.699
	Ginbil & Zeamay	324.42	322.699	328.467	330-323	Ginbil & Zeamay	201.75	182.417	227.494
	Ambtri & Zeamay	128.24	125.964	129.186	129-125	Ambtri & Zeamay	260.823	243.999	274.85
	Sargla & Zeamay	107.591	101.479	113.114	MIN: 125	Sargla & Zeamay	105.88	99.501	111.833
	Aratha & Zeamay	91.8245	84.4808	98.6075	MIN: 113	Aratha & Zeamay	88.3127	80.6419	96.0216
	Botbra & Botter	356.977	355.994	357.999	358-356	Botbra & Botter	288.795	184.5	376.223
	Ulvmut & Volcar	969.589	943.564	1026.73	1056-948	Ulvmut & Volcar	860.079	761.919	965.852
	Acrsp & Plasp	464.237	458.033	470.004	470-458	Acrsp & Plasp	421.882	262.062	609.196
	Codfra & Bryhyp	559.356	496.783	637.673	MIN: 541	Codfra & Bryhyp	466.799	312	600.452
**LN**	Cyamer & Chocri	1161.31	1019.27	1402.89	MIN: 1047	Cyamer & Chocri	1034.47	910.463	1210.77
	Graliv & Chocri	666.323	574.054	774.607	MIN: 609	Graliv & Chocri	496.547	298.407	682.236
	Euaaff & Zygsp	549.13	462.488	646.949	MIN: 350	Euaaff & Zygsp	534.353	434.11	648.129
	Marpol & Zeamay	477.499	457.414	503.744	MIN: 480	Marpol & Zeamay	472.164	453.312	499.375
	Isoteg & Zeamay	421.647	419.208	423.086	423-419	Isoteg & Zeamay	634.638	562.2	715.253
	Ctesub & Zeamay	400.155	393.664	406.23	MIN: 385	Ctesub & Zeamay	400.545	393.835	406.672

	Ginbil & Zeamay	324.208	322.638	327.81	330-323	Ginbil & Zeamay	203.234	185.625	226.447
	Ambtri & Zeamay	128.177	125.85	129.173	129-125	Ambtri & Zeamay	252.241	232.518	269.816
	Sargla & Zeamay	103.245	96.1049	109.789	MIN: 125	Sargla & Zeamay	100.727	93.2888	107.565
	Aratha & Zeamay	85.0434	76.5844	92.9169	MIN: 113	Aratha & Zeamay	80.0408	71.172	88.6051
	Botbra & Botter	357.015	356.003	358.006	358-356	Botbra & Botter	383.526	222.225	528.343
	Ulvmut & Volcar	977.833	944.997	1041.4	1056-948	Ulvmut & Volcar	883.338	789.212	998.671
	Acrsp & Plasp	463.826	457.981	470.007	470-458	Acrsp & Plasp	397.606	264.9	544.128
	Codfra & Bryhyp	553.43	496.047	617.988	MIN: 541	Codfra & Bryhyp	474.178	316.368	599.976
**UGAM**	Cyamer & Chocri	1223.92	1035.5	1486	MIN: 1047	Cyamer & Chocri	1290.31	945.61	1811.38
	Graliv & Chocri	639.487	528.472	784.493	MIN: 609	Graliv & Chocri	327.096	212.33	505.245
	Euaaff & Zygsp	482.34	351.657	646.127	MIN: 350	Euaaff & Zygsp	460.57	311.79	620.859
	Marpol & Zeamay	494.817	453.403	565.535	MIN: 480	Marpol & Zeamay	479.162	445.69	545.38
	Isoteg & Zeamay	421.148	419.03	423.022	423-419	Isoteg & Zeamay	487.132	427.998	586.139
	Ctesub & Zeamay	389.396	373.153	403.378	MIN: 385	Ctesub & Zeamay	388.356	370.819	403.502
	Ginbil & Zeamay	325.874	322.907	329.859	330-323	Ginbil & Zeamay	229.798	180.824	300.184
	Ambtri & Zeamay	128.264	126.121	129.181	129-125	Ambtri & Zeamay	266.324	244.271	284.618
	Sargla & Zeamay	114.437	108.796	119.146	MIN: 125	Sargla & Zeamay	112.333	106.17	117.644
	Aratha & Zeamay	98.7207	91.0944	105.484	MIN: 113	Aratha & Zeamay	95.1662	85.6905	103.088
	Botbra & Botter	356.995	356.002	358	358-356	Botbra & Botter	169.229	86.4677	350.505
	Ulvmut & Volcar	967.637	943.327	1023.64	1056-948	Ulvmut & Volcar	802.362	709.158	912.656
	Acrsp & Plasp	463.713	457.962	469.973	470-458	Acrsp & Plasp	345.931	246.14	494.284
	Codfra & Bryhyp	550.489	461.043	648.054	MIN: 541	Codfra & Bryhyp	307.777	186.775	487.737
**WN**	Cyamer & Chocri	1551.18	1348.72	1781.33	MIN: 1047	Cyamer & Chocri	1616.28	1430.7	1827.68
	Graliv & Chocri	588.486	497.531	669.895	MIN: 609	Graliv & Chocri	436.117	305.081	574.632
	Euaaff & Zygsp	557.766	457.948	652.765	MIN: 350	Euaaff & Zygsp	547.87	455.133	648.247
	Marpol & Zeamay	518.493	464.848	586.718	MIN: 480	Marpol & Zeamay	513.735	448.357	586.479
	Isoteg & Zeamay	421.174	419.028	423.033	423-419	Isoteg & Zeamay	581.76	498.439	671.945
	Ctesub & Zeamay	393.502	367.624	415.465	MIN: 385	Ctesub & Zeamay	391.058	351.373	416.3
	Ginbil & Zeamay	326.133	322.946	329.938	330-323	Ginbil & Zeamay	264.947	204.929	328.446
	Ambtri & Zeamay	127.932	125.463	129.124	129-125	Ambtri & Zeamay	290.274	256.183	319.669
	Sargla & Zeamay	122.365	115.738	126.888	MIN: 125	Sargla & Zeamay	120.387	112.312	126.174
	Aratha & Zeamay	113.723	103.694	122.078	MIN: 113	Aratha & Zeamay	110.849	94.5254	122.095
	Botbra & Botter	356.988	356	357.999	358-356	Botbra & Botter	264.473	171.869	358.723
	Ulvmut & Volcar	972.006	944.007	1031.87	1056-948	Ulvmut & Volcar	889.293	787.758	1008.51
	Acrsp & Plasp	464.025	457.978	470.025	470-458	Acrsp & Plasp	480.526	375.136	583.346
	Codfra & Bryhyp	538.064	459.218	613.245	MIN: 541	Codfra & Bryhyp	415.719	309.274	553.57
**16 genes, CB**									
**CIR**	Cyamer & Chocri	1131.1	966.263	1375.31	MIN: 1047	Cyamer & Chocri	1035.8	892.92	1195.25
	Graliv & Chocri	640.866	560.305	735.014	MIN: 609	Graliv & Chocri	583.95	414.536	729.13
	Euaaff & Zygsp	521.616	407.905	616.987	MIN: 350	Euaaff & Zygsp	495.731	394.098	617.186
	Marpol & Zeamay	458.704	444.374	480.311	MIN:480	Marpol & Zeamay	455.269	443.038	472.375

	Isoteg & Zeamay	421.647	419.233	423.087	423-419	Isoteg & Zeamay	589.969	512.004	676.002
	Ctesub & Zeamay	403.552	397.989	408.458	MIN: 385	Ctesub & Zeamay	403.63	398.01	408.392
	Ginbil & Zeamay	324.24	322.678	327.963	330-323	Ginbil & Zeamay	210.987	192.811	233.983
	Ambtri & Zeamay	128.245	126.092	129.189	129-125	Ambtri & Zeamay	251.26	235.386	265.304
	Sargla & Zeamay	100.116	93.9534	105.596	MIN: 125	Sargla & Zeamay	98.4234	91.3139	104.301
	Aratha & Zeamay	84.3725	77.2805	90.8907	MIN: 113	Aratha & Zeamay	81.0201	74.2431	87.4056
	Botbra & Botter	356.99	355.999	358.003	358-356	Botbra & Botter	293.286	184.096	385.085
	Bryhyp & Volcar	980.611	945.9	1045.02	1056-948	Bryhyp & Volcar	978.883	945.8	1041.59
	Codfra & Bryhyp	554.187	493.531	627.173	MIN: 541	Codfra & Bryhyp	477.885	322.195	613.754
	Acrsp & Plasp	464.16	458.004	470.059	470-458	Acrsp & Plasp	363.605	225.027	569.356
**LN**	Cyamer & Chocri	1176.93	1008.32	1411.48	MIN: 1047	Cyamer & Chocri	1042.6	877.893	1205.74
	Graliv & Chocri	659.64	559.393	771.775	MIN: 609	Graliv & Chocri	481.638	303.236	635.196
	Euaaff & Zygsp	560.485	482.758	671.63	MIN: 350	Euaaff & Zygsp	554.26	460.557	672.654
	Marpol & Zeamay	470.869	453.53	494.824	MIN:480	Marpol & Zeamay	465.549	451.208	486.382
	Isoteg & Zeamay	421.71	419.26	423.098	423-419	Isoteg & Zeamay	622.451	556.118	721.449
	Ctesub & Zeamay	401.197	395.907	406.207	MIN: 385	Ctesub & Zeamay	401.261	396.141	406.045
	Ginbil & Zeamay	324.144	322.643	327.713	330-323	Ginbil & Zeamay	211.666	195.375	228.759
	Ambtri & Zeamay	128.128	125.729	129.165	129-125	Ambtri & Zeamay	244.014	227.685	258.094
	Sargla & Zeamay	95.5552	88.7853	101.838	MIN: 125	Sargla & Zeamay	92.7495	84.6288	99.5887
	Aratha & Zeamay	78.2807	70.4461	85.7146	MIN: 113	Aratha & Zeamay	73.2701	64.6175	81.633
	Botbra & Botter	357.003	355.999	358.001	358-356	Botbra & Botter	393.728	232.51	534.491
	Bryhyp & Volcar	983.758	946.004	1047.57	1056-948	Bryhyp & Volcar	985.764	945.928	1051.22
	Codfra & Bryhyp	548.356	498.086	613.119	MIN: 541	Codfra & Bryhyp	461.695	307.467	581.744
	Acrsp & Plasp	463.876	458.009	469.973	470-458	Acrsp & Plasp	358.069	242.745	482.933
**UGAM**	Cyamer & Chocri	1232.78	1041.69	1480.39	MIN: 1047	Cyamer & Chocri	1307.57	950.061	1645.5
	Graliv & Chocri	643.271	526.879	803.033	MIN: 609	Graliv & Chocri	309.405	200.57	501.187
	Euaaff & Zygsp	484.49	351.604	657.205	MIN: 350	Euaaff & Zygsp	451.152	292.657	605.6
	Marpol & Zeamay	488.791	449.235	559.207	MIN:480	Marpol & Zeamay	473.817	441.796	537.848
	Isoteg & Zeamay	421.105	419.019	423.017	423-419	Isoteg & Zeamay	469.535	417.555	551.08
	Ctesub & Zeamay	390.015	372.766	404.36	MIN: 385	Ctesub & Zeamay	388.255	369.803	404.165
	Ginbil & Zeamay	325.989	322.942	329.884	330-323	Ginbil & Zeamay	228.141	178.74	294.57
	Ambtri & Zeamay	128.293	126.143	129.185	129-125	Ambtri & Zeamay	267.643	248.531	285.729
	Sargla & Zeamay	111.045	105.333	115.854	MIN: 125	Sargla & Zeamay	109.344	102.712	114.394
	Aratha & Zeamay	95.5079	88.4983	102.245	MIN: 113	Aratha & Zeamay	91.9391	83.3564	100.156
	Botbra & Botter	357.007	356.004	358.005	358-356	Botbra & Botter	145.229	76.1317	304.625
	Bryhyp & Volcar	970.365	943.823	1031.91	1056-948	Bryhyp & Volcar	966.633	943.007	1019.81
	Codfra & Bryhyp	551.798	463.57	657.386	MIN: 541	Codfra & Bryhyp	288.263	161.113	461.219
	Acrsp & Plasp	463.714	457.945	469.947	470-458	Acrsp & Plasp	322.159	215.648	449.557
**WN**	Cyamer & Chocri	1588.44	1381.46	1824.85	MIN: 1047	Cyamer & Chocri	1676.71	1454.21	1896.68
	Graliv & Chocri	591.388	503.996	671.737	MIN: 609	Graliv & Chocri	452.862	332.72	587.865
	Euaaff & Zygsp	561.427	464.967	654.049	MIN: 350	Euaaff & Zygsp	549.885	459.055	642.331

	Marpol & Zeamay	507.775	458.099	573.907	MIN:480	Marpol & Zeamay	497.46	441.615	575.456
	Isoteg & Zeamay	421.131	419.024	423.026	423-419	Isoteg & Zeamay	531.15	452.585	612.455
	Ctesub & Zeamay	392.981	367.457	415.131	MIN: 385	Ctesub & Zeamay	389.013	351.055	415.04
	Ginbil & Zeamay	326.059	322.938	329.895	330-323	Ginbil & Zeamay	260.289	199.377	323.288
	Ambtri & Zeamay	127.925	125.441	129.118	129-125	Ambtri & Zeamay	290.577	252.734	318.264
	Sargla & Zeamay	122.385	115.899	126.969	MIN: 125	Sargla & Zeamay	120.368	113.207	126.227
	Aratha & Zeamay	113.97	104.2	122.186	MIN: 113	Aratha & Zeamay	110.782	93.7865	122.961
	Botbra & Botter	356.971	355.996	358	358-356	Botbra & Botter	253.209	159.979	342.878
	Bryhyp & Volcar	989.835	946.781	1051.2	1056-948	Bryhyp & Volcar	984.682	945.908	1050.65
	Codfra & Bryhyp	531.838	446.248	603.729	MIN: 541	Codfra & Bryhyp	393.077	289.398	508.717
	Acrsp & Plasp	463.927	457.987	469.994	470-458	Acrsp & Plasp	452.667	346.877	567.009
	**16 genes, ML**								
**CIR**	Cyamer & Chocri	1089.37	926.805	1331.98	MIN: 1047	Cyamer & Chocri	989.912	842.626	1155.24
	Graliv & Chocri	638.132	564.623	725.578	MIN: 609	Graliv & Chocri	573.385	424.342	715.036
	Euaaff & Zygsp	528.962	394.568	634.544	MIN: 350	Euaaff & Zygsp	522.403	388.753	639.001
	Marpol & Zeamay	462.13	446.79	485.517	MIN: 480	Marpol & Zeamay	457.201	443.505	475.595
	Isoteg & Zeamay	421.659	419.2	423.097	423-419	Isoteg & Zeamay	590.487	524.401	664.066
	Ctesub & Zeamay	403.746	398.094	408.529	MIN: 385	Ctesub & Zeamay	403.695	398.007	408.752
	Ginbil & Zeamay	324.261	322.683	328.086	330-323	Ginbil & Zeamay	204.302	186.266	226.654
	Ambtri & Zeamay	128.264	126.077	129.176	129-125	Ambtri & Zeamay	254.984	239.868	270.003
	Sargla & Zeamay	100.456	94.4046	105.953	MIN: 125	Sargla & Zeamay	98.3489	91.5966	104.651
	Aratha & Zeamay	85.0202	78.2047	91.5794	MIN: 113	Aratha & Zeamay	81.6565	73.7152	89.495
	Botbra & Botter	356.996	356	358.002	358-356	Botbra & Botter	298.188	180.292	390.907
	Ulvmut & Volcar	979.384	945.431	1042.6	1056-948	Ulvmut & Volcar	859.319	776.244	927.312
	Acrsp & Plasp	464.129	458.029	469.997	470-458	Acrsp & Plasp	406.872	260.033	593.036
	Codfra & Bryhyp	543.778	483.383	610.362	MIN: 541	Codfra & Bryhyp	441.682	317.82	571.291
**LN**	Cyamer & Chocri	1132.71	975.461	1342.13	MIN: 1047	Cyamer & Chocri	971.816	837.749	1099.43
	Graliv & Chocri	658.921	565.28	753.681	MIN: 609	Graliv & Chocri	503.586	335.449	669.27
	Euaaff & Zygsp	564.817	484.914	679.523	MIN: 350	Euaaff & Zygsp	558.352	458.83	669.394
	Marpol & Zeamay	474.18	456.795	497.951	MIN: 480	Marpol & Zeamay	468.401	453.504	491.809
	Isoteg & Zeamay	421.717	419.253	423.099	423-419	Isoteg & Zeamay	625.495	566.528	703.812
	Ctesub & Zeamay	401.124	395.906	406.074	MIN: 385	Ctesub & Zeamay	401.115	395.693	406.085
	Ginbil & Zeamay	324.168	322.631	327.833	330-323	Ginbil & Zeamay	205.272	188.546	224.597
	Ambtri & Zeamay	128.146	125.79	129.17	129-125	Ambtri & Zeamay	248.362	230.477	264.355
	Sargla & Zeamay	95.2551	88.2655	101.596	MIN: 125	Sargla & Zeamay	92.7671	85.5522	99.4067
	Aratha & Zeamay	78.1337	69.989	85.6711	MIN: 113	Aratha & Zeamay	72.9788	64.4727	81.8903
	Botbra & Botter	356.997	356.002	357.999	358-356	Botbra & Botter	388.573	217.667	528.077
	Ulvmut & Volcar	987.507	946.663	1051.59	1056-948	Ulvmut & Volcar	902.349	797.241	998.431
	Acrsp & Plasp	463.893	457.984	469.933	470-458	Acrsp & Plasp	370.745	244.189	531.717
	Codfra & Bryhyp	541.256	484.161	594.573	MIN: 541	Codfra & Bryhyp	408.864	257.098	549.319
**UGAM**	Cyamer & Chocri	1216.33	1039.04	1454.19	MIN: 1047	Cyamer & Chocri	1378.52	986.724	1653.23

	Graliv & Chocri	644.591	535.02	795.853	MIN: 609	Graliv & Chocri	323.765	212.879	490.754
	Euaaff & Zygsp	493.738	352.502	670.363	MIN: 350	Euaaff & Zygsp	473.07	318.36	626.502
	Marpol & Zeamay	493.29	451.394	566.974	MIN: 480	Marpol & Zeamay	475.967	445.515	539.582
	Isoteg & Zeamay	421.104	419.01	423.011	423-419	Isoteg & Zeamay	473.74	416.111	563.914
	Ctesub & Zeamay	389.661	372.755	404.186	MIN: 385	Ctesub & Zeamay	388.554	368.138	404.518
	Ginbil & Zeamay	325.966	322.914	329.886	330-323	Ginbil & Zeamay	226.707	175.341	297.569
	Ambtri & Zeamay	128.303	126.191	129.205	129-125	Ambtri & Zeamay	270.904	251.517	287.466
	Sargla & Zeamay	111.5	106.023	116.242	MIN: 125	Sargla & Zeamay	109.806	103.557	115.465
	Aratha & Zeamay	96.14	88.873	102.674	MIN: 113	Aratha & Zeamay	92.7664	83.6386	100.453
	Botbra & Botter	356.993	355.993	357.998	358-356	Botbra & Botter	155.735	77.0947	311.346
	Ulvmut & Volcar	969.125	943.643	1027.45	1056-948	Ulvmut & Volcar	813.06	716.458	933.745
	Acrsp & Plasp	463.725	457.968	469.959	470-458	Acrsp & Plasp	326.084	220.526	468.915
	Codfra & Bryhyp	541.841	448.686	631.963	MIN: 541	Codfra & Bryhyp	267.527	152.147	443.702
**WN**	Cyamer & Chocri	1519.33	1316.87	1740.33	MIN: 1047	Cyamer & Chocri	1595.8	1392.45	1831.59
	Graliv & Chocri	594.154	508.986	673.558	MIN: 609	Graliv & Chocri	462.711	332.36	587.992
	Euaaff & Zygsp	572.617	478.274	664.017	MIN: 350	Euaaff & Zygsp	561.812	465.023	663.093
	Marpol & Zeamay	515.665	463.195	583.763	MIN: 480	Marpol & Zeamay	510.779	449.871	591.586
	Isoteg & Zeamay	421.141	419.021	423.021	423-419	Isoteg & Zeamay	545.804	459.988	630.03
	Ctesub & Zeamay	393.051	367.433	415.226	MIN: 385	Ctesub & Zeamay	389.817	350.065	415.948
	Ginbil & Zeamay	326.119	322.946	329.947	330-323	Ginbil & Zeamay	258.834	195.142	317.925
	Ambtri & Zeamay	127.937	125.464	129.134	129-125	Ambtri & Zeamay	294.655	260.913	321.338
	Sargla & Zeamay	122.427	115.997	126.904	MIN: 125	Sargla & Zeamay	120.337	112.596	125.855
	Aratha & Zeamay	114.027	104.275	122.02	MIN: 113	Aratha & Zeamay	110.833	93.3744	121.757
	Botbra & Botter	356.975	355.999	357.997	358-356	Botbra & Botter	259.756	161.679	347.313
	Ulvmut & Volcar	985.031	946.161	1047.2	1056-948	Ulvmut & Volcar	940.791	840.629	1054.37
	Acrsp & Plasp	463.996	458.005	469.996	470-458	Acrsp & Plasp	471.61	367.18	584.546
	Codfra & Bryhyp	516.452	422.254	589.645	MIN: 541	Codfra & Bryhyp	371.292	282.308	489.936
	**Normal run - Removed fossils**					**Fossil cross validation run - Removed fossils**			
	**Node of common ancestor**	**Mn. date**	**Lower 95% HPDI**	**Upper 95% HPDI**	**Fossil age calibration (MY)**	**Mn. date**	**Lower 95% HPDI**	**Upper 95% HPDI**	
**8 genes, CB**									
**CIR**	Caulen & Caucyl	413.881	308.162	504.154	MIN: 505	Caulen & Caucyl	198.698	113.535	327.891
	Oedcar & Oedfov	385.033	381.654	392.024	393-382	Oedcar & Oedfov	66.6015	41.4781	103.728
	Stihel & Aphrep	108.34	103.081	110.649	110-97	Stihel & Aphrep	498.913	378.012	611.5
**LN**	Caulen & Caucyl	491.272	385.452	558.602	MIN: 505	Caulen & Caucyl	239.259	142.897	384.288
	Oedcar & Oedfov	386.737	381.882	392.93	393-382	Oedcar & Oedfov	74.3321	44.7977	114.596
	Stihel & Aphrep	106.8	98.5285	110.57	110-97	Stihel & Aphrep	568.632	471.829	637.825
**UGAM**	Caulen & Caucyl	515.11	417.461	616.733	MIN: 505	Caulen & Caucyl	161.687	94.4988	308.344
	Oedcar & Oedfov	387.195	381.974	392.948	393-382	Oedcar & Oedfov	76.138	38.1205	127.213
	Stihel & Aphrep	103.702	97.0804	110.08	110-97				

**WN**	Caulen & Caucyl	408.765	291.14	511.338	MIN: 505	Caulen & Caucyl	225.594	124.611	328.976
	Oedcar & Oedfov	386.445	381.798	392.692	393-382	Oedcar & Oedfov	104.102	48.6765	163.416
	Stihel & Aphrep	104.059	97.0082	110.209	110-97	Stihel & Aphrep	327.781	214.543	429.635
**8 genes, ML**									
**CIR**	Caulen & Caucyl	396.869	285.636	494.854	MIN: 505	Caulen & Caucyl	209.669	113.736	336.908
	Oedcar & Oedfov	385.104	381.628	392.145	393-382	Oedcar & Oedfov	71.5391	42.6155	108.683
	Stihel & Aphrep	108.35	103.242	110.721	110-97	Stihel & Aphrep	493.622	382.817	598.593
**LN**	Caulen & Caucyl	474.614	358.82	552.137	MIN: 505	Caulen & Caucyl	251.32	156.537	364.929
	Oedcar & Oedfov	386.483	381.894	392.825	393-382	Oedcar & Oedfov	81.9049	47.9545	130.953
	Stihel & Aphrep	106.482	97.7906	110.605	110-97	Stihel & Aphrep	575.897	473.179	645.887
**UGAM**	Caulen & Caucyl	509.189	405.478	606.142	MIN: 505	Caulen & Caucyl	162.786	97.114	298.064
	Oedcar & Oedfov	387.037	381.898	392.979	393-382	Oedcar & Oedfov	80.1214	41.3946	142.513
	Stihel & Aphrep	104.066	97.1068	110.18	110-97	Stihel & Aphrep	235.176	97.5333	466.721
**WN**	Caulen & Caucyl	399.055	283.965	508.146	MIN: 505	Caulen & Caucyl	222.868	126.284	326.133
	Oedcar & Oedfov	386.383	381.899	392.867	393-382	Oedcar & Oedfov	109.803	52.3767	173.521
	Stihel & Aphrep	104.222	97.1346	110.144	110-97	Stihel & Aphrep	338.573	229.812	451.493
**16 genes, CB**									
**CIR**	Caulen & Caucyl	375.665	260.471	461.974	MIN: 505	Caulen & Caucyl	198.044	113.029	315.26
	Oedcar & Oedfov	384.894	381.678	391.754	393-382	Oedcar & Oedfov	57.4257	36.1327	89.4941
	Stihel & Aphrep	109.03	105.665	110.978	110-97	Stihel & Aphrep	592.925	463.292	681.582
**LN**	Caulen & Caucyl	479.946	359.26	550.935	MIN: 505	Caulen & Caucyl	235.615	148.737	349.956
	Oedcar & Oedfov	386.689	381.959	392.919	393-382	Oedcar & Oedfov	68.6022	41.6347	110.18
	Stihel & Aphrep	107.229	99.3214	110.53	110-97	Stihel & Aphrep	603.341	527.012	669.248
**UGAM**	Caulen & Caucyl	514.466	418.231	605.673	MIN: 505	Caulen & Caucyl	137.436	80.3688	273.924
	Oedcar & Oedfov	387.147	382.004	392.962	393-382	Oedcar & Oedfov	69.8496	33.043	122.83
	Stihel & Aphrep	104.09	97.2736	110.17	110-97	Stihel & Aphrep	261.747	104.58	467.918
**WN**	Caulen & Caucyl	373.529	272.235	485.025	MIN: 505	Caulen & Caucyl	198.56	110.714	301.72
	Oedcar & Oedfov	386.374	381.862	392.721	393-382	Oedcar & Oedfov	104.334	47.8335	169.647
	Stihel & Aphrep	104.44	97.4831	110.114	110-97	Stihel & Aphrep	364.232	258.511	479.844
**16 genes, ML**									
**CIR**	Caulen & Caucyl	351.177	250.985	443.95	MIN: 505	Caulen & Caucyl	200.863	117.999	300.811
	Oedcar & Oedfov	384.866	381.449	391.807	393-382	Oedcar & Oedfov	58.9204	37.6046	89.453
	Stihel & Aphrep	109.065	105.314	111.08	110-97	Stihel & Aphrep	613.969	505.909	701.03
**LN**	Caulen & Caucyl	466.975	346.922	547.145	MIN: 505	Caulen & Caucyl	251.32	156.537	364.929
	Oedcar & Oedfov	386.772	381.898	392.851	393-382	Oedcar & Oedfov	81.9049	47.9545	130.953
	Stihel & Aphrep	107.443	99.9763	110.577	110-97	Stihel & Aphrep	575.897	473.179	645.887
**UGAM**	Caulen & Caucyl	507.449	403.101	597.306	MIN: 505	Caulen & Caucyl	162.786	97.114	298.064
	Oedcar & Oedfov	387.269	381.987	393.057	393-382	Oedcar & Oedfov	80.1214	41.3946	142.513
	Stihel & Aphrep	103.958	96.9547	110.266	110-97	Stihel & Aphrep	235.176	97.5333	466.721
**WN**	Caulen & Caucyl	354.671	257.32	474.437	MIN: 505	Caulen & Caucyl	222.868	126.284	326.133

	Oedcar & Oedfov	386.31	381.678	392.697	393-382	Oedcar & Oedfov	109.803	52.3767	173.521
	Stihel & Aphrep	104.45	97.1732	110.187	110-97	Stihel & Aphrep	338.573	229.812	451.493

Table S3. ITS pairwise sequence comparisons via NCBI BLAST between *Tetrabaena* and *Volvox* strains for genes used in the present study.

Gene Alignment	Taxa	Perc. Identity
ITS	T. socialis NIES-571 & NIES-691	92.47
	P. starrii NIES-1362 & NIES-1363	91.25
	V. carteri f. nagariensis NIES-865 & HK10	98.06

Table S4. Pairwise sequence comparisons between *Tetrabaena*, *Pleodorina*, and *Volvox* strains for genes used in the present study.

Gene Alignment	Taxa	Perc. Identity	Perc. Similarity
OG0004441	T. socialis NIES-571 & NIES-691	97.08	97.92
	P. starrii NIES-1362 & NIES-1363	100	100
	V. carteri f. nagariensis NIES-865 & HK10	100	100
OG0004570	T. socialis NIES-571 & NIES-691	95.86	96.55
	P. starrii NIES-1362 & NIES-1363	100	100
	V. carteri f. nagariensis NIES-865 & HK10	100	100
OG0004572	T. socialis NIES-571 & NIES-691	90.72	98.84
	P. starrii NIES-1362 & NIES-1363	N/A	N/A
	V. carteri f. nagariensis NIES-865 & HK10	100	100
OG0004581	T. socialis NIES-571 & NIES-691	N/A	N/A
	P. starrii NIES-1362 & NIES-1363	100	100
	V. carteri f. nagariensis NIES-865 & HK10	100	100
OG0004602	T. socialis NIES-571 & NIES-691	99	99.5
	P. starrii NIES-1362 & NIES-1363	100	100
	V. carteri f. nagariensis NIES-865 & HK10	100	100
OG0004910	T. socialis NIES-571 & NIES-691	N/A	N/A
	P. starrii NIES-1362 & NIES-1363	100	100
	V. carteri f. nagariensis NIES-865 & HK10	100	100
OG0004914	T. socialis NIES-571 & NIES-691	97.79	99.12
	P. starrii NIES-1362 & NIES-1363	100	100
	V. carteri f. nagariensis NIES-865 & HK10	100	100
OG0004916	T. socialis NIES-571 & NIES-691	N/A	N/A
	P. starrii NIES-1362 & NIES-1363	100	100
	V. carteri f. nagariensis NIES-865 & HK10	100	100
OG0005090	T. socialis NIES-571 & NIES-691	N/A	N/A
	P. starrii NIES-1362 & NIES-1363	100	100
	V. carteri f. nagariensis NIES-865 & HK10	100	100
OG0005111	T. socialis NIES-571 & NIES-691	97.79	98.69
	P. starrii NIES-1362 & NIES-1363	100	100
	V. carteri f. nagariensis NIES-865 & HK10	100	100
OG0005154	T. socialis NIES-571 & NIES-691	N/A	N/A
	P. starrii NIES-1362 & NIES-1363	N/A	N/A
	V. carteri f. nagariensis NIES-865 & HK10	100	100
OG0005180	T. socialis NIES-571 & NIES-691	97.43	98.2
	P. starrii NIES-1362 & NIES-1363	100	100
	V. carteri f. nagariensis NIES-865 & HK10	100	100
OG0005188	T. socialis NIES-571 & NIES-691	97.69	98.85
	P. starrii NIES-1362 & NIES-1363	100	100

	V. carteri f. nagariensis NIES-865 & HK10	100	100
OG0005211	T. socialis NIES-571 & NIES-691	88.68	92.45
	P. starrii NIES-1362 & NIES-1363	100	100
	V. carteri f. nagariensis NIES-865 & HK10	100	100
OG0005213	T. socialis NIES-571 & NIES-691	N/A	N/A
	P. starrii NIES-1362 & NIES-1363	100	100
	V. carteri f. nagariensis NIES-865 & HK10	100	100
OG0005213	T. socialis NIES-571 & NIES-691	99.27	99.27
	P. starrii NIES-1362 & NIES-1363	99.65	99.65
	V. carteri f. nagariensis NIES-865 & HK10	100	100

Table S5. Taxa chosen from Phytozome database to infer putative list of single-copy genes present across Rhodophyta, Streptophyta, and Chlorophyta.

Taxon	Clade	File type
Porphyra umbilicus	Rhodophyta	Longest primary transcript
Marchantia polymorpha	Streptophyta	Longest primary transcript
Selaginella moellendorffii	Streptophyta	Longest primary transcript
Amorella trichopoda	Streptophyta	Longest primary transcript
Arabidopsis thaliana	Streptophyta	Longest primary transcript
Oryza sativa	Streptophyta	Longest primary transcript
Micromonas pusilla	Chlorophyta	Longest primary transcript
Coccomyxa subellipsoidea	Chlorophyta	Longest primary transcript
Chlamydomonas reinhardtii	Chlorophyta	Longest primary transcript
Volvox carteri	Chlorophyta	Longest primary transcript

Table S6. Best evolutionary model as predicted by ProtTest under the Akaike Information Criterion for 263 gene dataset.

Evolutionary model	Gene ID(s)
LG+I+G	OG0004735, OG0005210, OG0004727, OG0004711, OG0005234, OG0004916, OG0005037, OG0004500, OG0004463, OG0005025, OG0004471, OG0005146, OG0004571, OG0005113, OG0005013, OG0005170, OG0005001, OG0005101, OG0004994, OG0005162, OG0004379, OG0005044, OG0005139, OG0005039, OG0005127, OG0004557, OG0005060, OG0004996, OG0004445, OG0004791, OG0004853, OG0004953, OG0004629, OG0004762, OG0004814, OG0004770, OG0004818, OG0005193, OG0004613, OG0004695, OG0005168, OG0005064, OG0004787, OG0004565, OG0005152, OG0004577, OG0004569, OG0005089, OG0005243, OG0005197, OG0004617, OG0004973, OG0004873, OG0004910, OG0004778, OG0004678, OG0004945, OG0004750, OG0004650, OG0004857, OG0004934, OG0004607, OG0004912, OG0005199, OG0004871, OG0004615, OG0004594, OG0004640, OG0004855, OG0005206, OG0004731, OG0004959, OG0005218, OG0004990, OG0005178, OG0005066, OG0005166, OG0004371, OG0005117, OG0005009, OG0005142, OG0004579, OG0004999, OG0005071, OG0004546, OG0004792, OG0004692, OG0004378, OG0005063, OG0004680, OG0005000, OG0005128, OG0004401, OG0004570, OG0005024, OG0004513, OG0005159, OG0004966, OG0005244, OG0004978, OG0005256, OG0004602, OG0005203, OG0004638, OG0004645, OG0004833, OG0004815, OG0005180, OG0004700, OG0005080, OG0004763, OG0005254, OG0004876, OG0004976, OG0005246, OG0005213, OG0004831, OG0004952, OG0004852, OG0004747, OG0004581, OG0004655, OG0004755, OG0004435, OG0004782, OG0004790, OG0005173, OG0005026, OG0004572, OG0005134, OG0005149, OG0005049, OG0004632, OG0004927, OG0004944, OG0004848, OG0004720, OG0004956, OG0004489, OG0004903, OG0005188, OG0004779, OG0004811, OG0004911, OG0004667, OG0005030, OG0005153, OG0004694, OG0005114, OG0004540, OG0004893, OG0005106, OG0004517, OG0004466, OG0005151, OG0005032, OG0005175, OG0004688, OG0004696, OG0004641, OG0004495, OG0004825, OG0004487, OG0005207, OG0005198, OG0005186, OG0005231, OG0005240, OG0004677
LG+G+F	OG0005091, OG0004894, OG0004455, OG0004534, OG0004965, OG0004799, OG0004516, OG0004780, OG0004726, OG0005205, OG0004839, OG0005165
LG+I+G+F	OG0005226, OG0005154, OG0004447, OG0005070, OG0005062, OG0004528, OG0005156, OG0005160, OG0005111, OG0004658, OG0004737, OG0004977, OG0004914, OG0004441, OG0004522, OG0005107, OG0004418, OG0004802, OG0004705, OG0005185, OG0004949, OG0004719, OG0004619, OG0004963, OG0004955, OG0005121, OG0005033, OG0005050, OG0004429, OG0004554, OG0005090, OG0005227, OG0004702, OG0005235, OG0005211, OG0004600, OG0004807, OG0004919, OG0005201, OG0004940, OG0004985, OG0005110, OG0004411, OG0005157, OG0004485, OG0004585, OG0004743, OG0005221, OG0004972, OG0004519, OG0005022, OG0005041, OG0004881, OG0004993, OG0004405, OG0005004, OG0004784, OG0004796, OG0004958, OG0004765, OG0004777

LG+G	OG0005083, OG0004808, OG0005144, OG0005236, OG0004845, OG0005099, OG0004764, OG0004847, OG0004685, OG0005171, OG0004968, OG0005145, OG0004948, OG0004991
WAG+I+G	OG0004428, OG0005010
WAG+I+G+F	OG0005074
JTT+I+G+F	OG0004909, OG0005102
JTT+G	OG0005126
WAG+G	OG0004383

Table S7. Sortadate results for ML and CB analyses.

ML			
**Name**	**Root-to-tip_var**	**Treelength**	**Bipartition**
OG0005111	0.006282	10.7575	0.567901
OG0004581	0.031017	21.0253	0.530864
OG0005180	0.009017	18.8066	0.512346
OG0005188	0.012531	15.8956	0.506173
OG0004572	0.006266	14.5625	0.493827
OG0004602	0.009665	16.0539	0.493827
OG0004441	0.069715	16.7154	0.493827
OG0004914	0.011281	17.4935	0.487654
OG0004570	0.032717	16.7553	0.487654
OG0005211	0.014826	17.5303	0.481481
OG0005213	0.013159	13.5909	0.469136
OG0005254	0.02567	22.882	0.469136
OG0005090	0.02657	14.4124	0.469136
OG0004916	0.019781	13.0302	0.462963
OG0005154	0.011618	25.0841	0.45679
OG0004910	0.020995	18.4409	0.45679
**CB**			
**Name**	**Root-to-tip_var**	**Treelength**	**Bipartition**
OG0005111	0.006282	10.7575	0.561728
OG0005180	0.009017	18.8066	0.518519
OG0004581	0.031017	21.0253	0.518519
OG0004572	0.006266	14.5625	0.506173
OG0004441	0.069715	16.7154	0.506173
OG0005188	0.012531	15.8956	0.493827
OG0005211	0.014826	17.5303	0.493827
OG0004602	0.009665	16.0539	0.487654
OG0005254	0.02567	22.882	0.481481
OG0004570	0.032717	16.7553	0.481481
OG0004914	0.011281	17.4935	0.475309
OG0005213	0.013159	13.5909	0.475309
OG0005039	0.030911	15.5965	0.475309
OG0005090	0.02657	14.4124	0.469136
OG0004916	0.019781	13.0302	0.45679
OG0005013	0.010171	15.3026	0.450617
