# Supplementary material for: Fossil-calibrated molecular clock data enable reconstruction of steps leading to differentiated multicellularity and anisogamy in the Volvocine algae
Source: BMC Biol. 2024 Apr 10;22:79. doi: 10.1186/s12915-024-01878-1 (PMC11007952; doi:10.1186/s12915-024-01878-1)
Supplement: Supplementary file 3 — Additional File 3: Supplementary Methods. [file 12915_2024_1878_MOESM3_ESM.pdf]

Additional File 3: Supplementary Methods

Fossil selections and node calibrations make possible molecular-clock analyses and the construction of an evolutionary time tree. Multiple sources of information can be used to calibrate a phylogenetic tree when performing a molecular dating analysis. These forms of information fall into three distinct categories [113]: verifiable terrestrial geological events [114], secondary calibrations (those inferred in a separate study under another molecular dataset and calibration point) [115], and/or fossil taxa. While no calibration source is without its limitations, well-preserved fossils with distinguishing features that are either assignable to or that establish a relationship with extant taxa are highly favored. The fossilized remains of a taxon usually represent when a particular organism rose to abundance rather than when it emerged, thereby providing a minimum age constraint on the node to which it has been assigned [116].

Since no reliable fossils exist for the volvocine algae, we selected 14 fossil taxa across the Archaeplastida where reliable fossils are abundant (**Figure 2** and **Table 1**). For each primary fossil calibration used in this study, each calibration point was constrained to a range rather than a fixed-point estimate, acknowledging the inherent uncertainty in fossil ages. With each date range, we specified a soft bound where 2.5% of the total probability mass is positioned outside of the specified lower and/or upper bounds [117].

The root age of our tree, the divergence between red and green algae, was maximally constrained to 2000 MYA. This maximum constraint was informed by geological evidence indicating a rise in atmospheric oxygen ~2400 MYA [118]; we reason that it is unlikely that a common ancestor of red and green algae existed prior to elevated oxygen levels in the atmosphere and at the ocean surface. Since this date is most likely removed from the true root

age of the Archaeplastida by hundreds of millions of years, this maximum is fitting for an upper constraint.

Two fossil calibrations were assigned within the Rhodophyta (red algae). The oldest divergence within the rhodophyte lineage was constrained to a minimum age of 1047 MYA [26] based on *Bangiomorpha pubscens* [119], the oldest red algal fossil reliably identified as such. Florideophyte divergence (620-600 MYA) was established by the age of unambiguous florideophytes in the Doushantuo Formation, China, and that node was calibrated using a minimum age of 609 MYA [120].

Within Streptophyta, the clade composed of charophyte green algae and land plants, 8 nodes were calibrated using the fossil record. The oldest divergence within the Zygnemataceae was constrained to a minimum age of 350 MYA based on the oldest fossils assigned to this clade [121]. The dawn of land plants was informed by newly discovered fossil spores from the Ordovician [122] (MIN:480 MYA). *Cooksonia* with tracheids [123] (423-419 MYA) aided in estimating the emergence of vascular plants (i.e. tracheophytes), while the oldest known seeds [100] (MIN: 385 MYA) were used to calibrate the fern-seed plant split. The gymnosperm-angiosperm bifurcation was constrained to 330-323 MYA, based on the oldest *Cordaite,* a sister to coifiers [101], whereas the age of the *Amborella* and Nymplaeles divergence from other angiosperms was informed by Barremian flowers and pollen [102] (129-125 MYA). Finally, the split between chloroanthaceae/magnoliid and monocots + eudicots was calibrated using the oldest known chloranthaceous fossils [103] (MIN: 125 MYA), while the monocot-eudicot split was constrained to a minimum age of 113 MYA based on *Liliacidites* monocot pollen [99].

Among the Chlorophyta, a major Archaeplastida clade consisting of the unicellular and multicellular green algae, 4 nodes were calibrated. Within Trebouxiophyceae, the oldest

Botryococcus divergence was informed by the earliest known fossils assigned to that genus (358-356 MYA) [121]. *Proterocladus*, a cladophoracean fossil, was used to constrain the divergence between the Ulvophyceae and Bryopsidales + Chlorophyceae to 1056-948 MYA [25]. Within the Ulvophyceae, the earliest Ulotrichale divergence was constrained to 470-458 MYA based on *Vermiporella* [104]. Lastly, *Protocodium* was used to constrain the divergence between *Codium* and *Bryopsis* to a minimum age of 541 MYA [105].

Three fossil taxa were discarded from our analysis because they yielded either highly variable estimates when fossil cross validation tests were performed. These validation tests involve a "leave-one-out" approach where a node's calibration is removed and the resulting inferred mean date and 95% HPD interval is compared to the fossil date. The oldest split within the Caulerpaceae was intended to be informed by *Margerita dorus* [106] (MIN: 505), the deepest divergence in the Caulerpaceae was estimated to have a mean date of ~200 MYA when this fossil taxa was removed (**Supplemental Table 2**). This fossil's phylogenetic placement is reliable, but nodes ages inferred using our *Caulerpa* molecular data do not accord with the age of the *Margerita dorus* fossil. An earlier inferred date compared to the fossil age suggests that this fossil was formed during a later period; therefore, we also deemed this fossil to be uninformative. The *Oedogonium* chlorophyte clade was originally going to be constrained to 393-382 MYA, based on *Paleoodogonium* [107]; however, fossil validation tests estimated ~58 MYA as the deepest divergence for the genus *Oedogonium* (**Supplemental Table 2**). Based on their morphology [107, 108] and similarities in degradation [108] to extant *Oedogonium*, *Paleoodogoinum* fossils appear to be a reliable ancestor of *Oedogonium*. Our inferred validation dates indicate that *Paleoodogoinum* may be even more distantly related to *Oedogonium* than previously thought, thus we removed this fossil from consideration as a calibration point. The

earliest known divergence within the Chaetophorales was intended to be informed by *Electrophycus astroplethus* (110-97 MYA) [109], however, the oldest divergence within the Chaetophorales was estimated to be much older at ~613 MYA (**Supplemental Table 2**).

Phylogenies of the Archaeplastida inferred by different analytical methods are largely congruent. Within Rhodophyta, our analyses indicate *Galdieria* + *Cyanidiococcus* + *Cyanidioschyzon* as sister to the Bangiophyceae + Florideophyceae with all branches having high support (ML bootstraps (MLBS) = 100, Bayesian posterior probabilities (BPP) and Coalescent posterior probabilities >= 0.97). Within the Streptophyta, all three analyses inferred an identical branching order, which in "newick" tree format [110] consists of (Klebsormidiales,(Characeae,(Coleochaetophyceae,(Zygnemataceae, Embryophyta)))). Regarding the position occupied by the Coleochaetophyceae, our ML + BI and CB trees indicate this clade to be sister to Zygnemataceae + Embryophyta with high support (MLBS = 100, BPP = 1.0, CPP = 1.0). Thus, our results show that the filamentous charophyte Zygnemataceae is the sister group to land plants, consistent with other published works [111, 112, 124]. Among the chlorophyte green algae, our analyses also show that *Tetraselmis* is sister to the three main Chlorophyta families: Trebouxiophyceae, Ulvophyceae, and Chlorophyceae. And lastly, our ML + BI and CB analyses do not accord in the position of the ulvophycean Bryopsidales. All three analyses indicate that the Ulvophyceae are paraphyletic, as noted in recent studies [75, 125] based on dense ulvophyte taxonomic sampling. ML and BI analyses indicate that the Bryopsidales are sister to the Chlorophyceae (MLBS = 93, BPP = 1.0), as previously reported [75, 125]. Our CB tree, however, suggests a branching order of Bryopsidales sister to Ulvophyceae I + Chlorophyceae (CPP = 1.0). Due to these findings, we suggest that the

Ulvophyceae are indeed paraphyletic, with the Bryopsidales likely a sister group to the Chlorophyceaen green algae.

Key Archaeplastida divergence times are largely congruent with previous studies. Beginning at the root, red and green algae diverged from their last common ancestor in the Early to Middle Mesoproterozoic (**Figure 2**), most likely ~1385 MYA (**Figure 2**), consistent with a Middle Mesoproterozoic estimate by Lang et al. [126] The red-green algal divergence has also been inferred to have occurred as early as the Late Paleoproterozoic [97, 127, 128], in the Early Mesoproterozoic [96, 129, 130], or as late at the Early Neoproterozoic [97]. Our 95% HPD intervals for the red green algal divergence (1634-1187 MYA) complement these earlier reports. Branching from the root, we find that two crown groups, Rhodophyta and Chlorophyta, emerge in the Late Mesoproterozoic, and Streptophyta, a crown group containing land plants, originated in the Early Neoproterozoic (**Figure 2**), as reported in Sánchez-Baracaldo et al. [127]. When considering the 95% HPD interval for each of the crown groups, our estimates encompass those of other studies. For example, Rhodophyta has a 95% HPD interval between 1331 (upper bound postdates the first fossil appearance of red algae by ~100 MY) and 926 MYA (**Figure 3B**), and this overlaps with estimates from a study exclusively sampling taxa within the Archaeplastida [131] as well as another that samples across all 3 domains of life [129]. The 95% HPD Confidence intervals for Chlorophyta and Streptophyta are between 1308 and 1102 MYA and 1038 and 858 MYA, respectively, and comfortably overlap with those from previous reports [96, 98, 132].

Key divergences within two crown groups overlap with inferred age estimates elsewhere. The Florideophyceae, the largest and most diverse clade of rhodophytes, were estimated to have

arisen sometime between 724 to 564 MYA (**Table 1**). Yang et al. [131], who performed dense taxonomic sampling of Rhodophyta, inferred that the Florideophyceae emerged in the Neoproterozoic, 879-681 MYA. Among the Streptophytes, land plants appear to originate either in the Late Cambrian or Late Ordovician, according to a 95% HPD interval spanning 485-446 MYA (**Table 1**). These estimates support claims by Strother and Foster [122] who hypothesized that embryophytes may have emerged earlier than the Ordovician following their discovery of a land plant fossil dating to the Early Ordovician. Additionally, our inferred 95% HPD estimates overlap those for Embryophyta reported by Morris et al. [98] Within Chlorophyta, the divergence between the Ulvophyceae I clade and Ulvophyceae II + other chlorophytes is estimated to have occurred sometime between 1042 and 945 MYA. The reason our 95% HPD intervals encompass the Early Neoproterozoic, and are outside those reported in other studies indicating a Mid Neoproterozoic divergence [17, 133], is because we imposed a fossil age constraint on *Proterocladus* of 1056-948 MYA.

Quality control of RNA-Seq reads. Using Trimmomatic v.0.39 [134], a 4-base sliding window approach was used to trim reads once average quality fell below a Phred score of 15; reads below a minimum length of 36 bases were discarded (LEADING: 3 TRAILING:3 SLIDINGWINDOW:4:15 MINLEN:36). To trim adapter content, the ILLUMINACLIP:2:30:10 option was used with the "adapters.fa" file provided in the BBMap suite due to its comprehensive list of adapter sequences. ILLUMINACLIP option allows for 2 "seed" mismatches where the seed is a short segment of the adapter that is being aligned in every section of the read. If >2 mismatches occurred, no trimming of the read occurred. Additionally,

there had to be at least 30 matched bases in the paired- end palindrome read alignment and at least 10 matched bases between an adapter sequence and read.

Annotation of de novo transcripts. Each assembled transcript had its longest open reading frame predicted using TransDecoder v5.5.0 (TransDecoder.LongOrfs -t <path to transcript file>). After the longest open reading frames were predicted for each transcript, the "longest_orfs.pep" file created by TransDecoder was subsequently used in a BLASTP search conducted by Diamond v2.0.11.149 [135] (diamond blastp -p <number of threads> -q longest_orfs.pep -d uniref90 -k 1 -e 0.00001 -o <.tsv outfile> --very-sensitive) against the Uniref90 database. Additional Diamond BLASTP options "k", "e", and "very-sensitive" correspond to max number of target sequences, e-value, and the sensitivity of Diamond when aligning longer sequences, respectively. Protein domain identification was accomplished by using HMMER v3.2.1 [136] (hmmscan -cpu <number of threads> --domtblout <outfile> <path to protein database> longest_orfs.pep) to conduct Pfam searches against the Pfam-A database using the "longest_orfs.pep" file as our input. The most likely coding region from each open read frame was predicted using TransDecoder v5.5.0 (TransDecoder.Predict -t <path to transcript file> --retain_pfam_hits <path to domtblout file> --retain_blastp_hits <path to BLASTP outfile>) by incorporating the results from our Diamond BLASTP and HMMER homology searches. As a result, nucleotide CDS and amino acid PEP files were created.

Orthologous gene identification infers 284 single-copy genes shared among red and green algae. Thes 10 taxa encompassing Rhodophyta, Streptophyta, and Chlorophyta were chosen based upon the availability of a longest primary transcript file, their respective clade in

Archaeplastida, and genome assembly quality. A primary transcript file was available on Phytozome for each chosen organism, and this first criterion is perhaps the most important due to alternative splice variants being accounted for in each genome. This limited our ability, however, to choose more than one rhodophyte. Nevertheless, we were still able to sample from all three major clades of Archaeplastida to infer a putative list of single-copy genes. Lastly, 4 out of the 10 genomes sampled have chromosome-level assembly and annotation, and a 5th, *Micromonas pusilla*, is in the targeted finish state with a 4 chromosome-level assembly with no gaps.

Ruling out putative organellar sequences from inferred single-copy gene dataset. All sequences identified as single-copy from our 10 genome training set were BLASTed against the NCBI non-redundant (nr) protein database (diamond makedb --in nr.gz --taxonmap prot.accession2taxid.FULL --taxonnodes nodes.dmp --taxonnames names.dmp -d nr) to ensure protein sequences identified as single-copy were also of nuclear origin using Diamond v2.0.11.149 [135] (diamond blastp -p 8 -q <query_file> -d nr -k 10 --outfmt 6 qseqid sseqid stitle staxids sscinames sskingdoms pident length mismatch gapopen qstart qend sstart send evalue bitscore -o <outfile> --very-sensitive --header). In total, 174 sequences contained at least one of the following terms identifying them as possible organellar sequences: “chloroplastic”, “chloroplast”, “mitochondrial” and/or “mitochondria”.

To address whether these sequences were of nuclear or organellar origin, GenPept files were fetched from NCBI using the protein accessions from the BLAST outfiles. For taxa, such as *Arabidopsis thaliana* and *Oryza sativa*, which have chromosome-level genome assemblies, all putative organellar proteins were shown to originate from the nuclear genome. An additional step of BLASTing these questionable sequences against the complete chloroplast genome found in

the NCBI chloroplast database from either the species or genus of the 10 taxa chosen for orthologous, single-copy gene identification. For those sequences, all BLAST results resulted in poor matches, high e-values, and low bit scores, indicating that these sequences are not originating from the plastid genome. For the putative mitochondrial sequences in our dataset, complete mitochondrial genomes for all 10 taxa could not be located. For those which could be found in the NCBI mitochondrial genome database, those sequences were BLASTed against its organism's mitochondrial genome. For all others, a broad BLAST search was done against all mitochondrial genomes. Again, all BLAST results indicated poor matches including high e-values and low bit scores for these sequences.

Gene sequence alignments. During manual alignment inspection, poorly aligned sequences that included bacterial, fungal, and non-algal sequences were removed according to the taxon ID assigned to a particular sequence's top BLAST result. Additionally, poorly aligned sequences that BLASTed to a similar sequence but different protein than the other well-aligned sequences were removed from each alignment to ensure that the same protein product was being compared and analyzed across taxa.

Parameters for molecular clock analysis. For all clock analyses, concatenated datasets of 8 and 16 genes were used to offset high computational cost. Each reduced molecular dataset was formed based on SortaDate [137] results of our 263 gene dataset where each single-gene phylogeny is scored and ranked on similarity to a given species tree (bipartition score), least amount of root-to-tip variance (clock-likeness), and overall tree length (calculable amount of molecular evolution). We sorted genes based upon their bipartition score.

Ancestral state reconstructions of discrete volvocine characters. In Phytools [138], stochastic character mapping ("simmap”) was applied to estimate probabilities of ancestral character states. For each analysis, the equal rates ("ER”) and all-rates-different ("ARD”) models of character evolution were compared using the R “anova” function. When used, this function outputs Akaike Information Criterion (AIC) values and Akaike weights for each model, and a higher Akaike weight indicates the favored model [139]. The highest weighted model was used for each “simmap” analysis where a total of 1000 stochastic simulations (nsim=1000) were conducted under an empirical Bayes method (Q=”empirical”). To conclude each run, the 1000 stochastic simulations were summarized into a single result, or tree, with calculated posterior probabilities as a pie chart at each internal node.

To further analyze ancestral character states in the volvocine algae, the MBASR toolkit [140], an R package, was used to estimate the previously mentioned discrete characters. The MBASR toolkit aims to circumvent the abstruse nature of ASR programs by automating the ASR workflow for discrete traits in Mr. Bayes [141]. For each ancestral state reconstruction, a Markov chain Monte Carlo (MCMC) simulation was conducted for 1,000,000 generations (MBASR n.samples=10,000) with a sample taken every 100 generations. Burn-in is automatically set by MBASR to discard samples whose log likelihoods score below the upper 25% threshold. All runs were conducted as “unordered” unless specified.
