# Supplementary material for: Fossil-calibrated molecular clock data enable reconstruction of steps leading to differentiated multicellularity and anisogamy in the Volvocine algae
Source: BMC Biol. 2024 Apr 10;22:79. doi: 10.1186/s12915-024-01878-1 (PMC11007952; doi:10.1186/s12915-024-01878-1)
Supplement: Supplementary file 1 — Additional File 1: Figures S1-S46. [file 12915_2024_1878_MOESM1_ESM.pdf]

Additional File 1: Supplementary Figures

Legends

Figure S1. Molecular phylogeny of the Archaeplastida (Rhodophyta, Streptophyta, and Chlorophyta) inferred through the maximum-likelihood method. The phylogenetic tree shown is based on a 263 multi-gene dataset of single-copy, protein-coding nuclear genes. Numbers on branches represent bootstrap values. Branch lengths correspond to genetic divergence, as indicated by the scale bar.

Figure S2. Phylogeny of the Archaeplastida (Rhodophyta, Streptophyta, and Chlorophyta) inferred via the coalescent-based method using 263 single-tree phylogenies. Numbers on branches represent posterior probabilities.

Figure S3. Molecular phylogeny of the Archaeplastida (Rhodophyta, Streptophyta, and Chlorophyta) inferred through Bayesian inference. The phylogenetic tree shown is based on a 263 multi-gene dataset of single-copy, protein-coding nuclear genes. Numbers on branches represent Bayesian posterior probabilities. Branch lengths correspond to genetic divergence, as indicated by the scale bar.

Figure S4. Scatter plot of mean dates for key nodes across Archaeplastida inferred under different clock models. The y-axis represents time in MYA (millions-of-years ago), and the x-axis corresponds to specific nodes labeled in table. The four clock models represented in each key node include: LN (blue), CIR (orange), WN (gray), and UGAM (yellow).

Figure S5. Ancestral state reconstruction of cellularity in the volvocine algae. Pie charts at nodes indicate the marginal likelihood from a continuous-time Markov Model implemented in MrBayes. Branch lengths, corresponding to time, were inferred under the CIR relaxed clock model using 16 most clock-like genes as determined by Sortadate, and branching order was determined via maximum-likelihood.

Figure S6. Ancestral state reconstruction of cellularity in the volvocine algae. Pie charts at nodes indicate the posterior probabilities from stochastic character mapping in Phytools. Branch lengths, corresponding to time, were inferred under the CIR relaxed clock model using 16 most clock-like genes as determined by Sortadate, and branching order was determined via maximum-likelihood.

Figure S7. Ancestral state reconstruction of cytokinesis in the volvocine algae. Pie charts at nodes indicate the marginal likelihood from a continuous-time Markov Model implemented in MrBayes. Branch lengths, corresponding to time, were inferred under the CIR relaxed clock model using 16 most clock-like genes as determined by Sortadate, and branching order was determined via maximum-likelihood.

Figure S8. Ancestral state reconstruction of cytokinesis in the volvocine algae. Pie charts at nodes indicate the posterior probabilities from stochastic character mapping in Phytools. Branch lengths, corresponding to time, were inferred under the CIR relaxed clock model using 16 most

clock-like genes as determined by Sortadate, and branching order was determined via maximum-likelihood.

Figure S9. Ancestral state reconstruction of basal body rotation in the volvocine algae. Pie charts at nodes indicate the marginal likelihood from a continuous-time Markov Model implemented in MrBayes. Branch lengths, corresponding to time, were inferred under the CIR relaxed clock model using 16 most clock-like genes as determined by Sortadate, and branching order was determined via maximum-likelihood.

Figure S10. Ancestral state reconstruction of basal body rotation in the volvocine algae. Pie charts at nodes indicate the posterior probabilities from stochastic character mapping in Phytools. Branch lengths, corresponding to time, were inferred under the CIR relaxed clock model using 16 most clock-like genes as determined by Sortadate, and branching order was determined via maximum-likelihood.

Figure S11. Ancestral state reconstruction of transformation of cell wall to extracellular matrix in the volvocine algae. Pie charts at nodes indicate the marginal likelihood from a continuous-time Markov Model implemented in MrBayes. Branch lengths, corresponding to time, were inferred under the CIR relaxed clock model using 16 most clock-like genes as determined by Sortadate, and branching order was determined via maximum-likelihood.

Figure S12. Ancestral state reconstruction of transformation of cell wall to extracellular matrix in the volvocine algae. Pie charts at nodes indicate the posterior probabilities from stochastic character mapping in Phytools. Branch lengths, corresponding to time, were inferred under the CIR relaxed clock model using 16 most clock-like genes as determined by Sortadate, and branching order was determined via maximum-likelihood.

Figure S13. Ancestral state reconstruction of genetic control of cell number in the volvocine algae. Pie charts at nodes indicate the marginal likelihood from a continuous-time Markov Model implemented in MrBayes. Branch lengths, corresponding to time, were inferred under the CIR relaxed clock model using 16 most clock-like genes as determined by Sortadate, and branching order was determined via maximum-likelihood.

Figure S14. Ancestral state reconstruction of genetic control of cell number in the volvocine algae. Pie charts at nodes indicate the posterior probabilities from stochastic character mapping in Phytools. Branch lengths, corresponding to time, were inferred under the CIR relaxed clock model using 16 most clock-like genes as determined by Sortadate, and branching order was determined via maximum-likelihood.

Figure S15. Ancestral state reconstruction of polarity in the volvocine algae. Pie charts at nodes indicate the marginal likelihood from a continuous-time Markov Model implemented in MrBayes. Branch lengths, corresponding to time, were inferred under the CIR relaxed clock model using 16 most clock-like genes as determined by Sortadate, and branching order was determined via maximum-likelihood.

Figure S16. Ancestral state reconstruction of polarity in the volvocine algae. Pie charts at nodes indicate the posterior probabilities from stochastic character mapping in Phytools. Branch lengths, corresponding to time, were inferred under the CIR relaxed clock model using 16 most clock-like genes as determined by Sortadate, and branching order was determined via maximum-likelihood.

Figure S17. Ancestral state reconstruction of spheroidal body structure in the volvocine algae. Pie charts at nodes indicate the marginal likelihood from a continuous-time Markov Model implemented in MrBayes. Branch lengths, corresponding to time, were inferred under the CIR relaxed clock model using 16 most clock-like genes as determined by Sortadate, and branching order was determined via maximum-likelihood.

Figure S18. Ancestral state reconstruction of spheroidal body structure in the volvocine algae. Pie charts at nodes indicate the posterior probabilities from stochastic character mapping in Phytools. Branch lengths, corresponding to time, were inferred under the CIR relaxed clock model using 16 most clock-like genes as determined by Sortadate, and branching order was determined via maximum-likelihood.

Figure S19. Ancestral state reconstruction of inversion in the volvocine algae as an unordered, 3-state problem. Pie charts at nodes indicate the marginal likelihood from a continuous-time Markov Model implemented in MrBayes. Branch lengths, corresponding to time, were inferred under the CIR relaxed clock model using 16 most clock-like genes as determined by Sortadate, and branching order was determined via maximum-likelihood.

Figure S20. Ancestral state reconstruction of inversion in the volvocine algae as an unordered, 3-state problem. Pie charts at nodes indicate the posterior probabilities from stochastic character mapping in Phytools. Branch lengths, corresponding to time, were inferred under the CIR relaxed clock model using 16 most clock-like genes as determined by Sortadate, and branching order was determined via maximum-likelihood.

Figure S21. Ancestral state reconstruction of inversion in the volvocine algae as an ordered, 3-state problem. . Pie charts at nodes indicate the marginal likelihood from a continuous-time Markov Model implemented in MrBayes. Branch lengths, corresponding to time, were inferred under the CIR relaxed clock model using 16 most clock-like genes as determined by Sortadate, and branching order was determined via maximum-likelihood.

Figure S22. Ancestral state reconstruction of inversion in the volvocine algae as an unordered, 2-state problem. . Pie charts at nodes indicate the marginal likelihood from a continuous-time Markov Model implemented in MrBayes. Branch lengths, corresponding to time, were inferred under the CIR relaxed clock model using 16 most clock-like genes as determined by Sortadate, and branching order was determined via maximum-likelihood.

Figure S23. Ancestral state reconstruction of inversion in the volvocine algae as an unordered, 2-state problem. Pie charts at nodes indicate the posterior probabilities from stochastic character mapping in Phytools. Branch lengths, corresponding to time, were inferred under the CIR

relaxed clock model using 16 most clock-like genes as determined by Sortadate, and branching order was determined via maximum-likelihood.

Figure S24. Ancestral state reconstruction of differentiation in the volvocine algae. . Pie charts at nodes indicate the marginal likelihood from a continuous-time Markov Model implemented in MrBayes. Branch lengths, corresponding to time, were inferred under the CIR relaxed clock model using 16 most clock-like genes as determined by Sortadate, and branching order was determined via maximum-likelihood.

Figure S25. Ancestral state reconstruction of differentiation in the volvocine algae. Pie charts at nodes indicate the posterior probabilities from stochastic character mapping in Phytools. Branch lengths, corresponding to time, were inferred under the CIR relaxed clock model using 16 most clock-like genes as determined by Sortadate, and branching order was determined via maximum-likelihood.

Figure S26. Ancestral state reconstruction of asymmetric division in the volvocine algae. Pie charts at nodes indicate the marginal likelihood from a continuous-time Markov Model implemented in MrBayes. Branch lengths, corresponding to time, were inferred under the CIR relaxed clock model using 16 most clock-like genes as determined by Sortadate, and branching order was determined via maximum-likelihood.

Figure S27. Ancestral state reconstruction of asymmetric division in the volvocine algae. Pie charts at nodes indicate the posterior probabilities from stochastic character mapping in Phytools. Branch lengths, corresponding to time, were inferred under the CIR relaxed clock model using 16 most clock-like genes as determined by Sortadate, and branching order was determined via maximum-likelihood.

Figure S28. Ancestral state reconstruction of a bifurcated cell division program in the volvocine algae. . Pie charts at nodes indicate the marginal likelihood from a continuous-time Markov Model implemented in MrBayes. Branch lengths, corresponding to time, were inferred under the CIR relaxed clock model using 16 most clock-like genes as determined by Sortadate, and branching order was determined via maximum-likelihood.

Figure S29. Ancestral state reconstruction of a bifurcated cell division program in the volvocine algae. Pie charts at nodes indicate the posterior probabilities from stochastic character mapping in Phytools. Branch lengths, corresponding to time, were inferred under the CIR relaxed clock model using 16 most clock-like genes as determined by Sortadate, and branching order was determined via maximum-likelihood.

Figure S30. Ancestral state reconstruction of meiotic product in the volvocine algae. Pie charts at nodes indicate the marginal likelihood from a continuous-time Markov Model implemented in MrBayes. Branch lengths, corresponding to time, were inferred under the CIR relaxed clock model using 16 most clock-like genes as determined by Sortadate, and branching order was determined via maximum-likelihood.

Figure S31. Ancestral state reconstruction of meiotic product in the volvocine algae. Pie charts at nodes indicate the posterior probabilities from stochastic character mapping in Phytools. Branch lengths, corresponding to time, were inferred under the CIR relaxed clock model using 16 most clock-like genes as determined by Sortadate, and branching order was determined via maximum-likelihood.

Figure S32. Ancestral state reconstruction of gametic differentiation in the volvocine algae as an unordered, 3-state problem. Pie charts at nodes indicate the marginal likelihood from a continuous-time Markov Model implemented in MrBayes. Branch lengths, corresponding to time, were inferred under the CIR relaxed clock model using 16 most clock-like genes as determined by Sortadate, and branching order was determined via maximum-likelihood.

Figure S33. Ancestral state reconstruction of gametic differentiation in the volvocine algae as an unordered, 3-state problem. Pie charts at nodes indicate the posterior probabilities from stochastic character mapping in Phytools. Branch lengths, corresponding to time, were inferred under the CIR relaxed clock model using 16 most clock-like genes as determined by Sortadate, and branching order was determined via maximum-likelihood.

Figure S34. Ancestral state reconstruction of gametic differentiation in the volvocine algae as an unordered, 2-state problem. Pie charts at nodes indicate the marginal likelihood from a continuous-time Markov Model implemented in MrBayes. Branch lengths, corresponding to time, were inferred under the CIR relaxed clock model using 16 most clock-like genes as determined by Sortadate, and branching order was determined via maximum-likelihood.

Figure S35. Ancestral state reconstruction of gametic differentiation in the volvocine algae as an unordered, 2-state problem. Pie charts at nodes indicate the posterior probabilities from stochastic character mapping in Phytools. Branch lengths, corresponding to time, were inferred under the CIR relaxed clock model using 16 most clock-like genes as determined by Sortadate, and branching order was determined via maximum-likelihood.

Figure S36. Ancestral state reconstruction of fertilization in the volvocine algae. Pie charts at nodes indicate the marginal likelihood from a continuous-time Markov Model implemented in MrBayes. Branch lengths, corresponding to time, were inferred under the CIR relaxed clock model using 16 most clock-like genes as determined by Sortadate, and branching order was determined via maximum-likelihood.

Figure S37. Ancestral state reconstruction of fertilization in the volvocine algae. Pie charts at nodes indicate the posterior probabilities from stochastic character mapping in Phytools. Branch lengths, corresponding to time, were inferred under the CIR relaxed clock model using 16 most clock-like genes as determined by Sortadate, and branching order was determined via maximum-likelihood.

Figure S38. Ancestral state reconstruction of monoecy vs. dioecy in the volvocine algae. Pie charts at nodes indicate the marginal likelihood from a continuous-time Markov Model implemented in MrBayes. Branch lengths, corresponding to time, were inferred under the CIR

relaxed clock model using 16 most clock-like genes as determined by Sortadate, and branching order was determined via maximum-likelihood.

Figure S39. Ancestral state reconstruction of monoecy vs. dioecy in the volvocine algae. Pie charts at nodes indicate the posterior probabilities from stochastic character mapping in Phytools. Branch lengths, corresponding to time, were inferred under the CIR relaxed clock model using 16 most clock-like genes as determined by Sortadate, and branching order was determined via maximum-likelihood.

Figure S40. Ancestral state reconstruction of selfing in the volvocine algae. Pie charts at nodes indicate the marginal likelihood from a continuous-time Markov Model implemented in MrBayes. Branch lengths, corresponding to time, were inferred under the CIR relaxed clock model using 16 most clock-like genes as determined by Sortadate, and branching order was determined via maximum-likelihood.

Figure S41. Ancestral state reconstruction of selfing in the volvocine algae. Pie charts at nodes indicate the posterior probabilities from stochastic character mapping in Phytools. Branch lengths, corresponding to time, were inferred under the CIR relaxed clock model using 16 most clock-like genes as determined by Sortadate, and branching order was determined via maximum-likelihood.

Figure S42. Ancestral state reconstruction of extrafertile females in the volvocine algae. Pie charts at nodes indicate the marginal likelihood from a continuous-time Markov Model implemented in MrBayes. Branch lengths, corresponding to time, were inferred under the CIR relaxed clock model using 16 most clock-like genes as determined by Sortadate, and branching order was determined via maximum-likelihood.

Figure S43. Ancestral state reconstruction of extrafertile females in the volvocine algae. Pie charts at nodes indicate the posterior probabilities from stochastic character mapping in Phytools. Branch lengths, corresponding to time, were inferred under the CIR relaxed clock model using 16 most clock-like genes as determined by Sortadate, and branching order was determined via maximum-likelihood.

Figure S44. Ancestral state reconstruction of dwarf males in the volvocine algae Pie charts at nodes indicate the marginal likelihood from a continuous-time Markov Model implemented in MrBayes. Branch lengths, corresponding to time, were inferred under the CIR relaxed clock model using 16 most clock-like genes as determined by Sortadate, and branching order was determined via maximum-likelihood.

Figure S45. Ancestral state reconstruction of dwarf males in the volvocine algae. Pie charts at nodes indicate the posterior probabilities from stochastic character mapping in Phytools. Branch lengths, corresponding to time, were inferred under the CIR relaxed clock model using 16 most clock-like genes as determined by Sortadate, and branching order was determined via maximum-likelihood.

Figure S46. Ancestral state reconstruction of dwarf males in the volvocine algae. Pie charts at nodes indicate the posterior probabilities from stochastic character mapping in Phytools inferred under the ER model. Branch lengths, corresponding to time, were inferred under the CIR relaxed clock model using 16 most clock-like genes as determined by Sortadate, and branching order was determined via maximum-likelihood.

Fig. S1

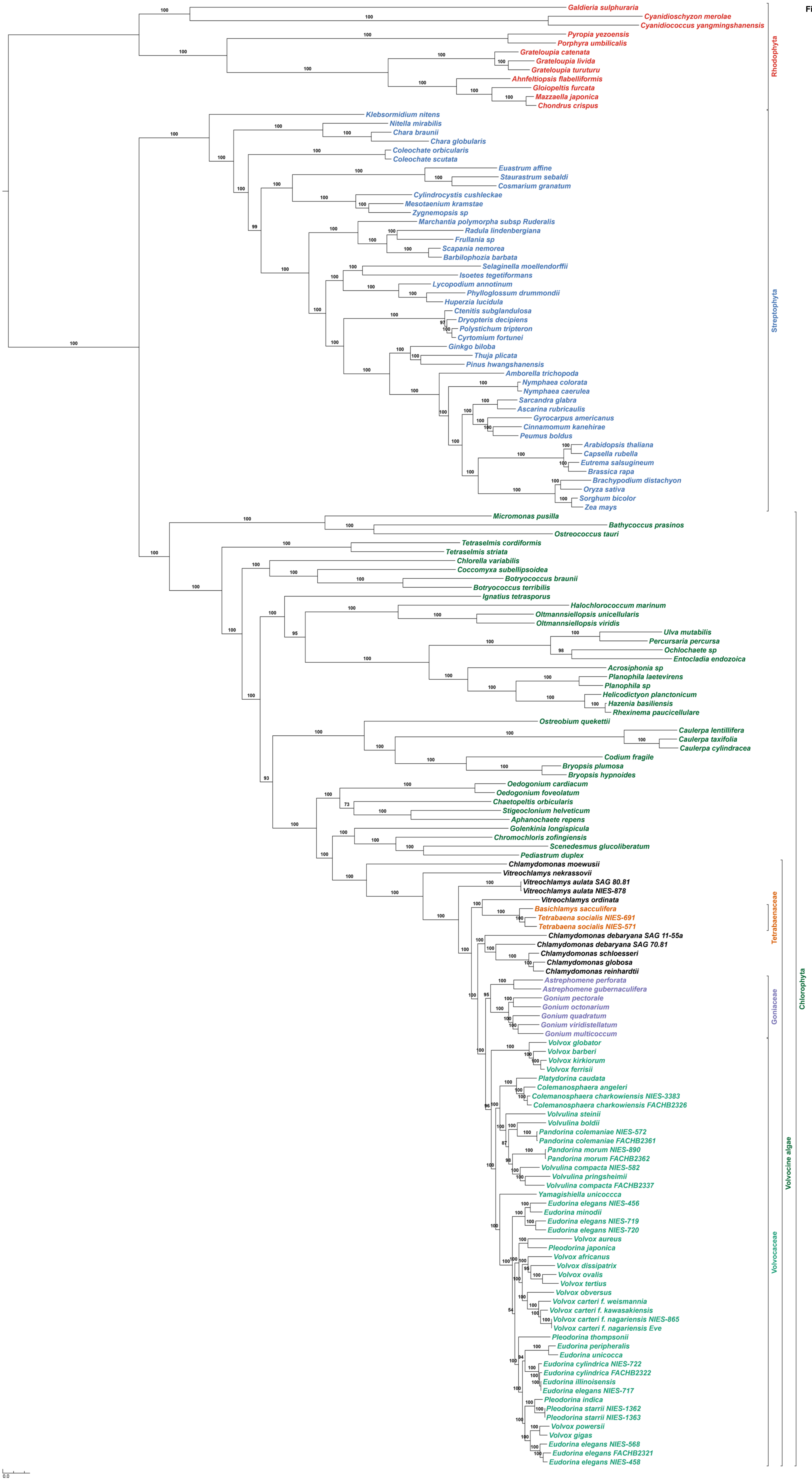
Galdieria sulphuraria
Cyanidioschyzon merolae
Cyanidiococcus yangmingshanensis
Pyropia yezoensis
Porphyra umbilicalis
Grateloupia catenata
Grateloupia livida
Grateloupia turuturu
Ahnfeltiopsis flabelliformis
Gloiopeltis furcata
Mazzaella japonica
Chondrus crispus
Rhodophyta
Klebsormidium nitens
Nitella mirabilis
Chara braunii
Chara globularis
Coleochate orbicularis
Coleochate scutata
Euastrum affine
Staurastrum sebaldi
Cosmarium granatum
Cylindrocystis cushleckae
Mesotaenium kramstae
Zygnemopsis sp
Marchantia polymorpha subsp Ruderalis
Radula lindenbergiana
Frullania sp
Scapania nemorea
Barbilophozia barbata
Selaginella moellendorffii
Isoetes tegetiformans
Lycopodium annotinum
Phylloglossum drummondii
Huperzia lucidula
Ctenitis subglandulosa
Dryopteris decipiens
Polystichum tripteron
Cyrtomium fortunei
Ginkgo biloba
Thuja plicata
Pinus hwangshanensis
Amborella trichopoda
Nymphaea colorata
Nymphaea caerulea
Sarcandra glabra
Ascarina rubricaulis
Gyrocarpus americanus
Cinnamomum kanehirae
Peumus boldus
Arabidopsis thaliana
Capsella rubella
Eutrema salsugineum
Brassica rapa
Brachypodium distachyon
Oryza sativa
Sorghum bicolor
Zea mays
Streptophyta
Micromonas pusilla
Bathycoccus prasinos
Ostreococcus tauri
Tetraselmis cordiformis
Tetraselmis striata
Chlorella variabilis
Coccomyxa subellipsoidea
Botryococcus braunii
Botryococcus terribilis
Ignatius tetrasporus
Halochlorococcum marinum
Oltmannsiellopsis unicellularis
Oltmannsiellopsis viridis
Ulva mutabilis
Percursaria percursa
Ochlochaete sp
Entocladia endozoica
Acrosiphonia sp
Planophila laetevirens
Planophila sp
Helicodictyon planctonicum
Hazenia basiliensis
Rhexinema paucicellulare
Ostreobium quekettii
Caulerpa lentillifera
Caulerpa taxifolia
Caulerpa cylindracea
Codium fragile
Bryopsis plumosa
Bryopsis hypnoides
Oedogonium cardiacum
Oedogonium foveolatum
Chaetopeltis orbicularis
Stigeoclonium helveticum
Aphanochaete repens
Golenkinia longispicula
Chromochloris zofingiensis
Scenedesmus glucoliberatum
Pediastrum duplex
Chlamydomonas moewusii
Vitreochlamys nekrassovii
Vitreochlamys aulata SAG 80.81
Vitreochlamys aulata NIES-878
Vitreochlamys ordinata
Basichlamys sacculifera
Tetrabaena socialis NIES-691
Tetrabaena socialis NIES-571
Tetrabaenaceae
Chlamydomonas debaryana SAG 11-55a
Chlamydomonas debaryana SAG 70.81
Chlamydomonas schloesseri
Chlamydomonas globosa
Chlamydomonas reinhardtii
Astrephomene perforata
Astrephomene gubernaculifera
Gonium pectorale
Gonium octonarium
Gonium quadratum
Gonium viridistellatum
Gonium multicoccum
Goniaceae
Volvox globator
Volvox barberi
Volvox kirkiorum
Volvox ferrisii
Platydorina caudata
Colemanosphaera angeleri
Colemanosphaera charkowiensis NIES-3383
Colemanosphaera charkowiensis FACHB2326
Volvulina steinii
Volvulina boldii
Pandorina colemaniae NIES-572
Pandorina colemaniae FACHB2361
Pandorina morum NIES-890
Pandorina morum FACHB2362
Volvulina compacta NIES-582
Volvulina pringsheimii
Volvulina compacta FACHB2337
Yamagishiella unicoccca
Eudorina elegans NIES-456
Eudorina minodii
Eudorina elegans NIES-719
Eudorina elegans NIES-720
Volvox aureus
Pleodorina japonica
Volvox africanus
Volvox dissipatrix
Volvox ovalis
Volvox tertius
Volvox obversus
Volvox carteri f. weismannia
Volvox carteri f. kawasakiensis
Volvox carteri f. nagariensis NIES-865
Volvox carteri f. nagariensis Eve
Pleodorina thompsonii
Eudorina peripheralis
Eudorina unicocca
Eudorina cylindrica NIES-722
Eudorina cylindrica FACHB2322
Eudorina illinoisensis
Eudorina elegans NIES-717
Pleodorina indica
Pleodorina starrii NIES-1362
Pleodorina starrii NIES-1363
Volvox powersii
Volvox gigas
Eudorina elegans NIES-568
Eudorina elegans FACHB2321
Eudorina elegans NIES-458
Volvocaceae
Volvocine algae
Chlorophyta
0.0

Fig. S2

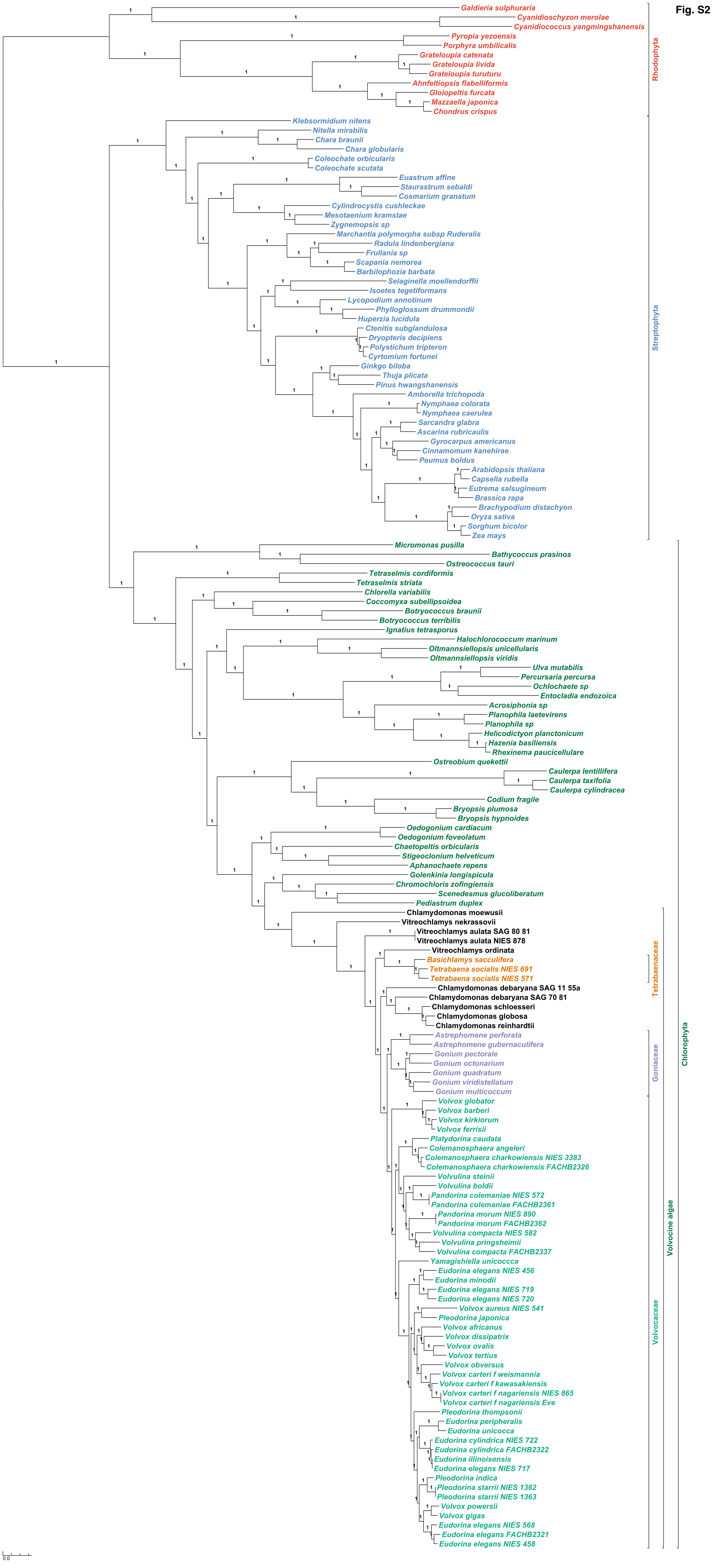
Galdieria sulphuraria
Cyanidioschyzon merolae
Cyanidiococcus yangmingshanensis
Pyropia yezoensis
Porphyra umbilicalis
Grateloupia catenata
Grateloupia livida
Grateloupia turuturu
Ahnfeltiopsis flabelliformis
Gloiopeltis furcata
Mazzaella japonica
Chondrus crispus
Rhodophyta
Klebsormidium nitens
Nitella mirabilis
Chara braunii
Chara globularis
Coleochate orbicularis
Coleochate scutata
Euastrum affine
Staurastrum sebaldi
Cosmarium granatum
Cylindrocystis cushleckae
Mesotaenium kramstae
Zygnemopsis sp
Marchantia polymorpha subsp Ruderalis
Radula lindenbergiana
Frullania sp
Scapania nemorea
Barbilophozia barbata
Selaginella moellendorffii
Isoetes tegetiformans
Lycopodium annotinum
Phylloglossum drummondii
Huperzia lucidula
Ctenitis subglandulosa
Dryopteris decipiens
Polystichum tripteron
Cyrtomium fortunei
Ginkgo biloba
Thuja plicata
Pinus hwangshanensis
Amborella trichopoda
Nymphaea colorata
Nymphaea caerulea
Sarcandra glabra
Ascarina rubricaulis
Gyrocarpus americanus
Cinnamomum kanehirae
Peumus boldus
Arabidopsis thaliana
Capsella rubella
Eutrema salsugineum
Brassica rapa
Brachypodium distachyon
Oryza sativa
Sorghum bicolor
Zea mays
Streptophyta
Micromonas pusilla
Bathycoccus prasinos
Ostreococcus tauri
Tetraselmis cordiformis
Tetraselmis striata
Chlorella variabilis
Coccomyxa subellipsoidea
Botryococcus braunii
Botryococcus terribilis
Ignatius tetrasporus
Halochlorococcum marinum
Oltmannsiellopsis unicellularis
Oltmannsiellopsis viridis
Ulva mutabilis
Percursaria percursa
Ochlochaete sp
Entocladia endozoica
Acrosiphonia sp
Planophila laetevirens
Planophila sp
Helicodictyon planctonicum
Hazenia basiliensis
Rhexinema paucicellulare
Ostreobium quekettii
Caulerpa lentillifera
Caulerpa taxifolia
Caulerpa cylindracea
Codium fragile
Bryopsis plumosa
Bryopsis hypnoides
Oedogonium cardiacum
Oedogonium foveolatum
Chaetopeltis orbicularis
Stigeoclonium helveticum
Aphanochaete repens
Golenkinia longispicula
Chromochloris zofingiensis
Scenedesmus glucoliberatum
Pediastrum duplex
Chlamydomonas moewusii
Vitreochlamys nekrassovii
Vitreochlamys aulata SAG 80 81
Vitreochlamys aulata NIES 878
Vitreochlamys ordinata
Basichlamys sacculifera
Tetrabaena socialis NIES 691
Tetrabaena socialis NIES 571
Tetrabaenaceae
Chlamydomonas debaryana SAG 11 55a
Chlamydomonas debaryana SAG 70 81
Chlamydomonas schloesseri
Chlamydomonas globosa
Chlamydomonas reinhardtii
Astrephomene perforata
Astrephomene gubernaculifera
Gonium pectorale
Gonium octonarium
Gonium quadratum
Gonium viridistellatum
Gonium multicoccum
Goniaceae
Volvox globator
Volvox barberi
Volvox kirkiorum
Volvox ferrisii
Platydorina caudata
Colemanosphaera angeleri
Colemanosphaera charkowiensis NIES 3383
Colemanosphaera charkowiensis FACHB2326
Volvulina steinii
Volvulina boldii
Pandorina colemaniae NIES 572
Pandorina colemaniae FACHB2361
Pandorina morum NIES 890
Pandorina morum FACHB2362
Volvulina compacta NIES 582
Volvulina pringsheimii
Volvulina compacta FACHB2337
Yamagishiella unicocca
Eudorina elegans NIES 456
Eudorina minodii
Eudorina elegans NIES 719
Eudorina elegans NIES 720
Volvox aureus NIES 541
Pleodorina japonica
Volvox africanus
Volvox dissipatrix
Volvox ovalis
Volvox tertius
Volvox obversus
Volvox carteri f weismannia
Volvox carteri f kawasakiensis
Volvox carteri f nagariensis NIES 865
Volvox carteri f nagariensis Eve
Pleodorina thompsonii
Eudorina peripheralis
Eudorina unicocca
Eudorina cylindrica NIES 722
Eudorina cylindrica FACHB2322
Eudorina illinoisensis
Eudorina elegans NIES 717
Pleodorina indica
Pleodorina starrii NIES 1362
Pleodorina starrii NIES 1363
Volvox powersii
Volvox gigas
Eudorina elegans NIES 568
Eudorina elegans FACHB2321
Eudorina elegans NIES 458
Volvocaceae
Volvocine algae
Chlorophyta
0.0

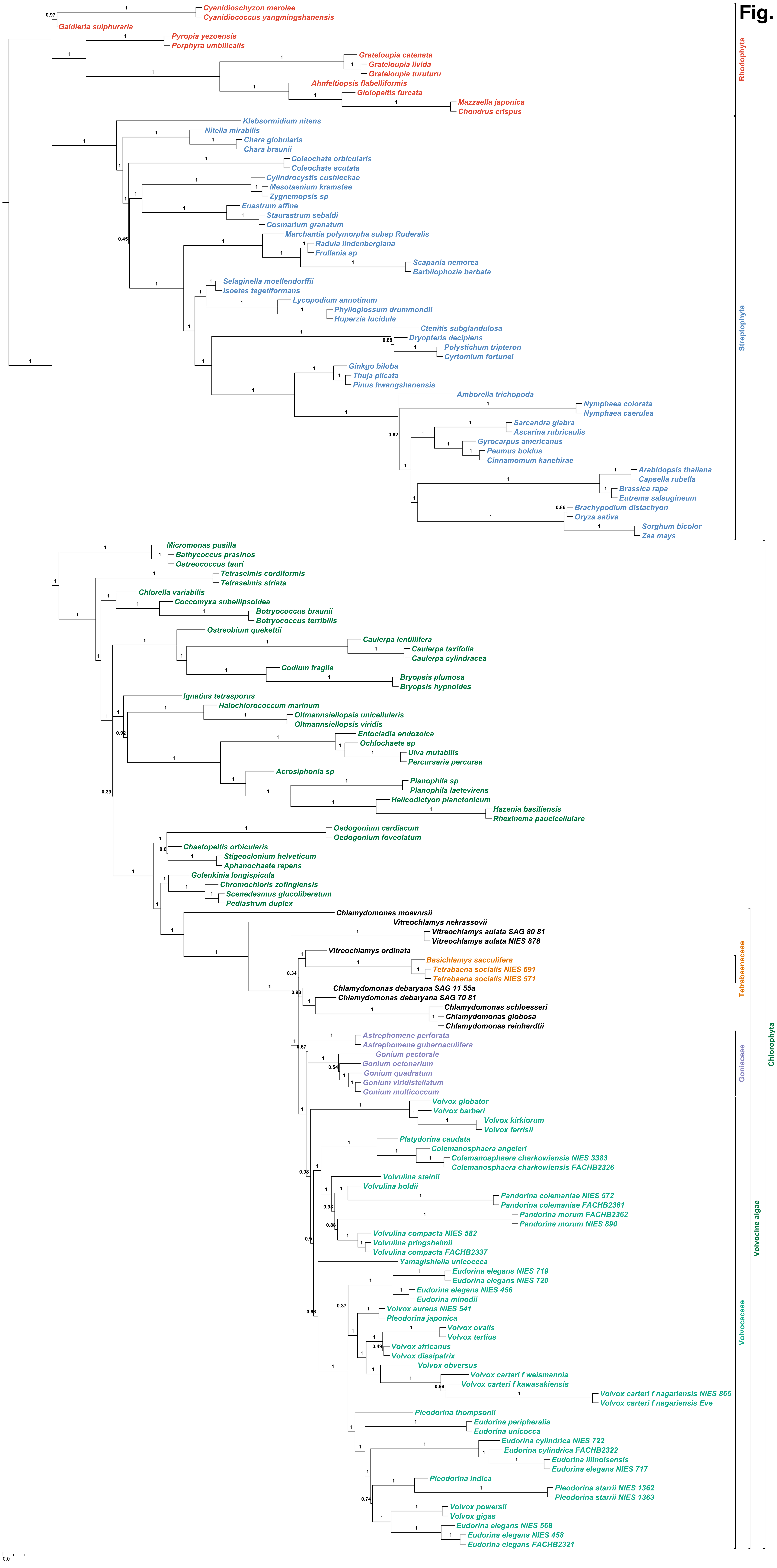
Cyanidioschyzon merolae
Cyanidiococcus yangmingshanensis
Galdieria sulphuraria
Pyropia yezoensis
Porphyra umbilicalis
Grateloupia catenata
Grateloupia livida
Grateloupia turuturu
Ahnfeltiopsis flabelliformis
Gloiopeltis furcata
Mazzaella japonica
Chondrus crispus
Rhodophyta
Klebsormidium nitens
Nitella mirabilis
Chara globularis
Chara braunii
Coleochate orbicularis
Coleochate scutata
Cylindrocystis cushleckae
Mesotaenium kramstae
Zygnemopsis sp
Euastrum affine
Staurastrum sebaldi
Cosmarium granatum
Marchantia polymorpha subsp Ruderalis
Radula lindenbergiana
Frullania sp
Scapania nemorea
Barbilophozia barbata
Selaginella moellendorffii
Isoetes tegetiformans
Lycopodium annotinum
Phylloglossum drummondii
Huperzia lucidula
Ctenitis subglandulosa
Dryopteris decipiens
Polystichum tripteron
Cyrtomium fortunei
Ginkgo biloba
Thuja plicata
Pinus hwangshanensis
Amborella trichopoda
Nymphaea colorata
Nymphaea caerulea
Sarcandra glabra
Ascarina rubricaulis
Gyrocarpus americanus
Peumus boldus
Cinnamomum kanehirae
Arabidopsis thaliana
Capsella rubella
Brassica rapa
Eutrema salsugineum
Brachypodium distachyon
Oryza sativa
Sorghum bicolor
Zea mays
Streptophyta
Micromonas pusilla
Bathycoccus prasinos
Ostreococcus tauri
Tetraselmis cordiformis
Tetraselmis striata
Chlorella variabilis
Coccomyxa subellipsoidea
Botryococcus braunii
Botryococcus terribilis
Ostreobium quekettii
Caulerpa lentillifera
Caulerpa taxifolia
Caulerpa cylindracea
Codium fragile
Bryopsis plumosa
Bryopsis hypnoides
Ignatius tetrasporus
Halochlorococcum marinum
Oltmannsiellopsis unicellularis
Oltmannsiellopsis viridis
Entocladia endozoica
Ochlochaete sp
Ulva mutabilis
Percursaria percursa
Acrosiphonia sp
Planophila sp
Planophila laetevirens
Helicodictyon planctonicum
Hazenia basiliensis
Rhexinema paucicellulare
Oedogonium cardiacum
Oedogonium foveolatum
Chaetopeltis orbicularis
Stigeoclonium helveticum
Aphanochaete repens
Golenkinia longispicula
Chromochloris zofingiensis
Scenedesmus glucoliberatum
Pediastrum duplex
Chlamydomonas moewusii
Vitreochlamys nekrassovii
Vitreochlamys aulata SAG 80 81
Vitreochlamys aulata NIES 878
Vitreochlamys ordinata
Basichlamys sacculifera
Tetrabaena socialis NIES 691
Tetrabaena socialis NIES 571
Chlamydomonas debaryana SAG 11 55a
Chlamydomonas debaryana SAG 70 81
Chlamydomonas schloesseri
Chlamydomonas globosa
Chlamydomonas reinhardtii
Astrephomene perforata
Astrephomene gubernaculifera
Gonium pectorale
Gonium octonarium
Gonium quadratum
Gonium viridistellatum
Gonium multicoccum
Volvox globator
Volvox barberi
Volvox kirkiorum
Volvox ferrisii
Platydorina caudata
Colemanosphaera angeleri
Colemanosphaera charkowiensis NIES 3383
Colemanosphaera charkowiensis FACHB2326
Volvulina steinii
Volvulina boldii
Pandorina colemaniae NIES 572
Pandorina colemaniae FACHB2361
Pandorina morum FACHB2362
Pandorina morum NIES 890
Volvulina compacta NIES 582
Volvulina pringsheimii
Volvulina compacta FACHB2337
Yamagishiella unicoccca
Eudorina elegans NIES 719
Eudorina elegans NIES 720
Eudorina elegans NIES 456
Eudorina minodii
Volvox aureus NIES 541
Pleodorina japonica
Volvox ovalis
Volvox tertius
Volvox africanus
Volvox dissipatrix
Volvox obversus
Volvox carteri f weismannia
Volvox carteri f kawasakiensis
Volvox carteri f nagariensis NIES 865
Volvox carteri f nagariensis Eve
Pleodorina thompsonii
Eudorina peripheralis
Eudorina unicocca
Eudorina cylindrica NIES 722
Eudorina cylindrica FACHB2322
Eudorina illinoisensis
Eudorina elegans NIES 717
Pleodorina indica
Pleodorina starrii NIES 1362
Pleodorina starrii NIES 1363
Volvox powersii
Volvox gigas
Eudorina elegans NIES 568
Eudorina elegans NIES 458
Eudorina elegans FACHB2321
Tetrabaenaceae
Goniaceae
Volvocaceae
Volvocine algae
Chlorophyta
0.0

Fig. S3

A.

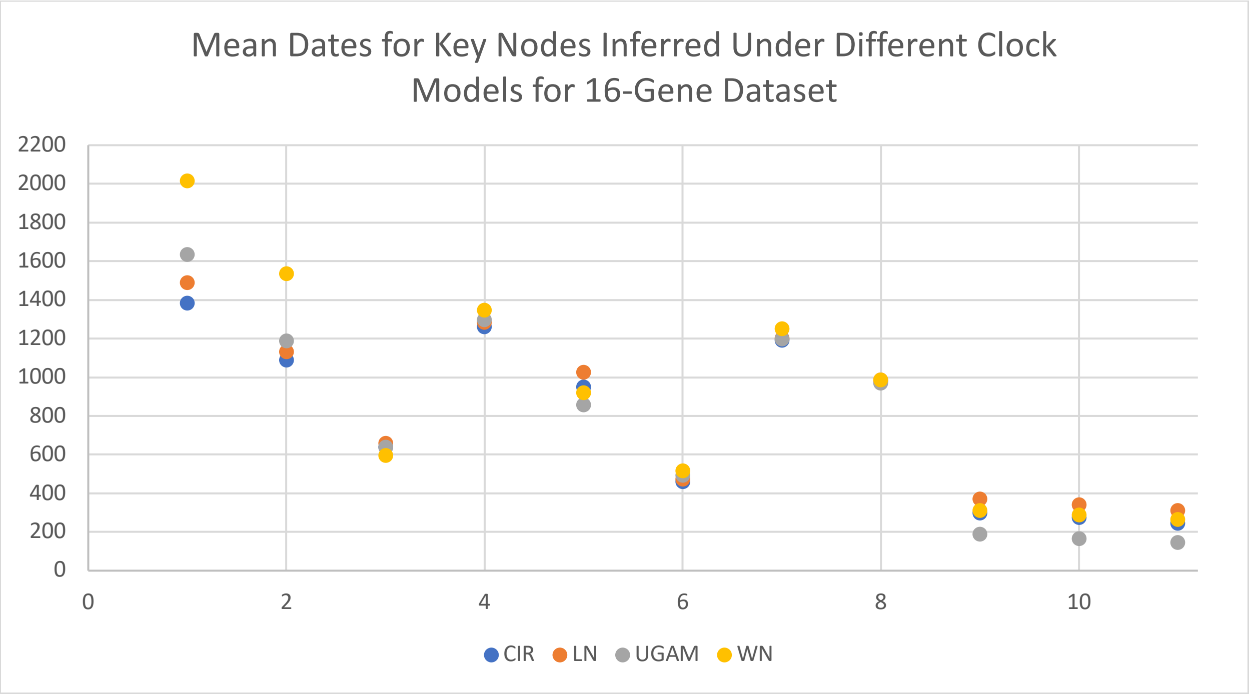

B.

	Node	Mean Date (MYA)	Lower 95% HPD (MYA)	Upper 95% HPD (MYA)
**Root**	**1**	**1385.09**	**1187.27**	**1634.25**
**Rhodophyta**	**2**	**1089.37**	**926.805**	**1331.98**
**Florideophyceae**	**3**	**638.132**	**564.623**	**725.578**
**Streptophyte-Chlorophyte Split**	**4**	**1263.2**	**1143.94**	**1415.51**
**Streptophyta**	**5**	**949.686**	**858.299**	**1038.22**
**Embryophyta (Land Plants)**	**6**	**462.13**	**446.79**	**485.517**
**Chlorophyta**	**7**	**1193.76**	**1102.16**	**1308.38**
**Ulvophyte I Split from Ulvophyte II + Chlorophyceae**	**8**	**979.384**	**945.431**	**1042.6**
**Tetrabaenaceae + V. ordinate Split from *Chlamydomonas* + *Volvox***	**9, A**	**298.995**	**237.332**	**349.105**
***Chlamydomonas* + *Volvox* divergence**	**10, B**	**274.199**	**214.672**	**323.722**
**Goniaceae + Volvocaceae Divergence**	**11, C**	**247.099**	**190.498**	**297.739**

Figure S4. Scatter plot of mean dates for key nodes across Archaeplastida inferred under different clock models. The y-axis represents time in MYA (millions-of-years ago), and the x-axis corresponds to specific nodes labeled in table. The four clock models represented in each key node include: LN (blue), CIR (orange), WN (gray), and UGAM (yellow).

Fig. S5

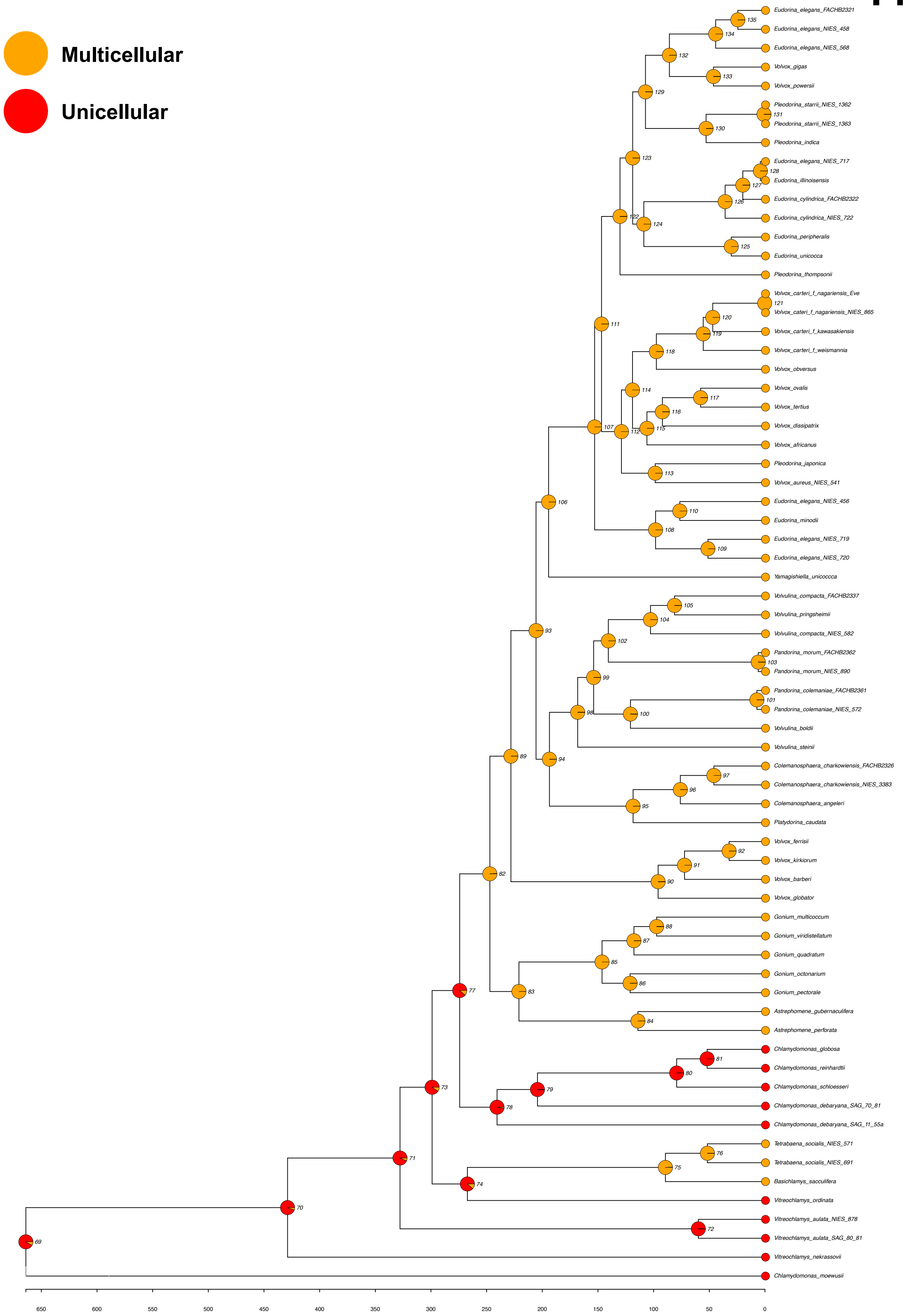
Multicellular
Unicellular
Eudorina_elegans_FACHB2321
Eudorina_elegans_NIES_458
Eudorina_elegans_NIES_568
Volvox_gigas
Volvox_powersii
Pleodorina_starrii_NIES_1362
Pleodorina_starrii_NIES_1363
Pleodorina_indica
Eudorina_elegans_NIES_717
Eudorina_illinoisensis
Eudorina_cylindrica_FACHB2322
Eudorina_cylindrica_NIES_722
Eudorina_peripheralis
Eudorina_unicocca
Pleodorina_thompsonii
Volvox_carteri_f_nagariensis_Eve
Volvox_cateri_f_nagariensis_NIES_865
Volvox_carteri_f_kawasakiensis
Volvox_carteri_f_weismannia
Volvox_obversus
Volvox_ovalis
Volvox_tertius
Volvox_dissipatrix
Volvox_africanus
Pleodorina_japonica
Volvox_aureus_NIES_541
Eudorina_elegans_NIES_456
Eudorina_minodii
Eudorina_elegans_NIES_719
Eudorina_elegans_NIES_720
Yamagishiella_unicoccca
Volvulina_compacta_FACHB2337
Volvulina_pringsheimii
Volvulina_compacta_NIES_582
Pandorina_morum_FACHB2362
Pandorina_morum_NIES_890
Pandorina_colemaniae_FACHB2361
Pandorina_colemaniae_NIES_572
Volvulina_boldii
Volvulina_steinii
Colemanosphaera_charkowiensis_FACHB2326
Colemanosphaera_charkowiensis_NIES_3383
Colemanosphaera_angeleri
Platydorina_caudata
Volvox_ferrisii
Volvox_kirkiorum
Volvox_barberi
Volvox_globator
Gonium_multicoccum
Gonium_viridistellatum
Gonium_quadratum
Gonium_octonarium
Gonium_pectorale
Astrephomene_gubernaculifera
Astrephomene_perforata
Chlamydomonas_globosa
Chlamydomonas_reinhardtii
Chlamydomonas_schloesseri
Chlamydomonas_debaryana_SAG_70_81
Chlamydomonas_debaryana_SAG_11_55a
Tetrabaena_socialis_NIES_571
Tetrabaena_socialis_NIES_691
Basichlamys_sacculifera
Vitreochlamys_ordinata
Vitreochlamys_aulata_NIES_878
Vitreochlamys_aulata_SAG_80_81
Vitreochlamys_nekrassovii
Chlamydomonas_moewusii
650
600
550
500
450
400
350
300
250
200
150
100
50
0

Fig. S6

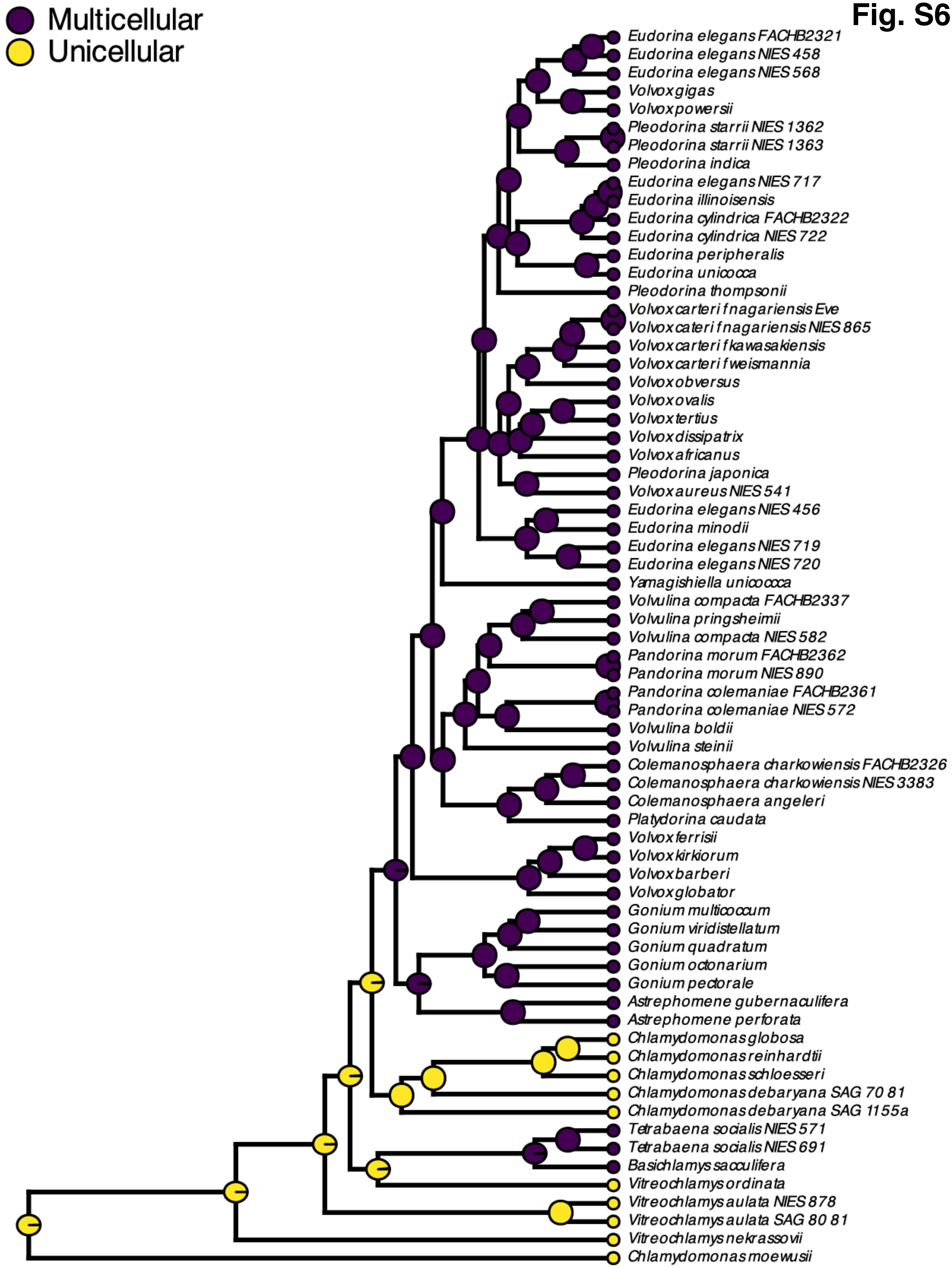

Multicellular
Unicellular
Eudorina elegans FACHB2321
Eudorina elegans NIES 458
Eudorina elegans NIES 568
Volvox gigas
Volvox powersii
Pleodorina starrii NIES 1362
Pleodorina starrii NIES 1363
Pleodorina indica
Eudorina elegans NIES 717
Eudorina illinoisensis
Eudorina cylindrica FACHB2322
Eudorina cylindrica NIES 722
Eudorina peripheralis
Eudorina unicocca
Pleodorina thompsonii
Volvox carteri f nagariensis Eve
Volvox cateri f nagariensis NIES 865
Volvox carteri f kawasakiensis
Volvox carteri f weismannia
Volvox obversus
Volvox ovalis
Volvox tertius
Volvox dissipatrix
Volvox africanus
Pleodorina japonica
Volvox aureus NIES 541
Eudorina elegans NIES 456
Eudorina minodii
Eudorina elegans NIES 719
Eudorina elegans NIES 720
Yamagishiella unicocca
Volvulina compacta FACHB2337
Volvulina pringsheimii
Volvulina compacta NIES 582
Pandorina morum FACHB2362
Pandorina morum NIES 890
Pandorina colemaniae FACHB2361
Pandorina colemaniae NIES 572
Volvulina boldii
Volvulina steinii
Colemanosphaera charkowiensis FACHB2326
Colemanosphaera charkowiensis NIES 3383
Colemanosphaera angeleri
Platydorina caudata
Volvox ferrisii
Volvox kirkiorum
Volvox barberi
Volvox globator
Gonium multicoccum
Gonium viridistellatum
Gonium quadratum
Gonium octonarium
Gonium pectorale
Astrephomene gubernaculifera
Astrephomene perforata
Chlamydomonas globosa
Chlamydomonas reinhardtii
Chlamydomonas schloesseri
Chlamydomonas debaryana SAG 70 81
Chlamydomonas debaryana SAG 1155a
Tetrabaena socialis NIES 571
Tetrabaena socialis NIES 691
Basichlamys sacculifera
Vitreochlamys ordinata
Vitreochlamys aulata NIES 878
Vitreochlamys aulata SAG 80 81
Vitreochlamys nekrassovii
Chlamydomonas moewusii

Fig. S7

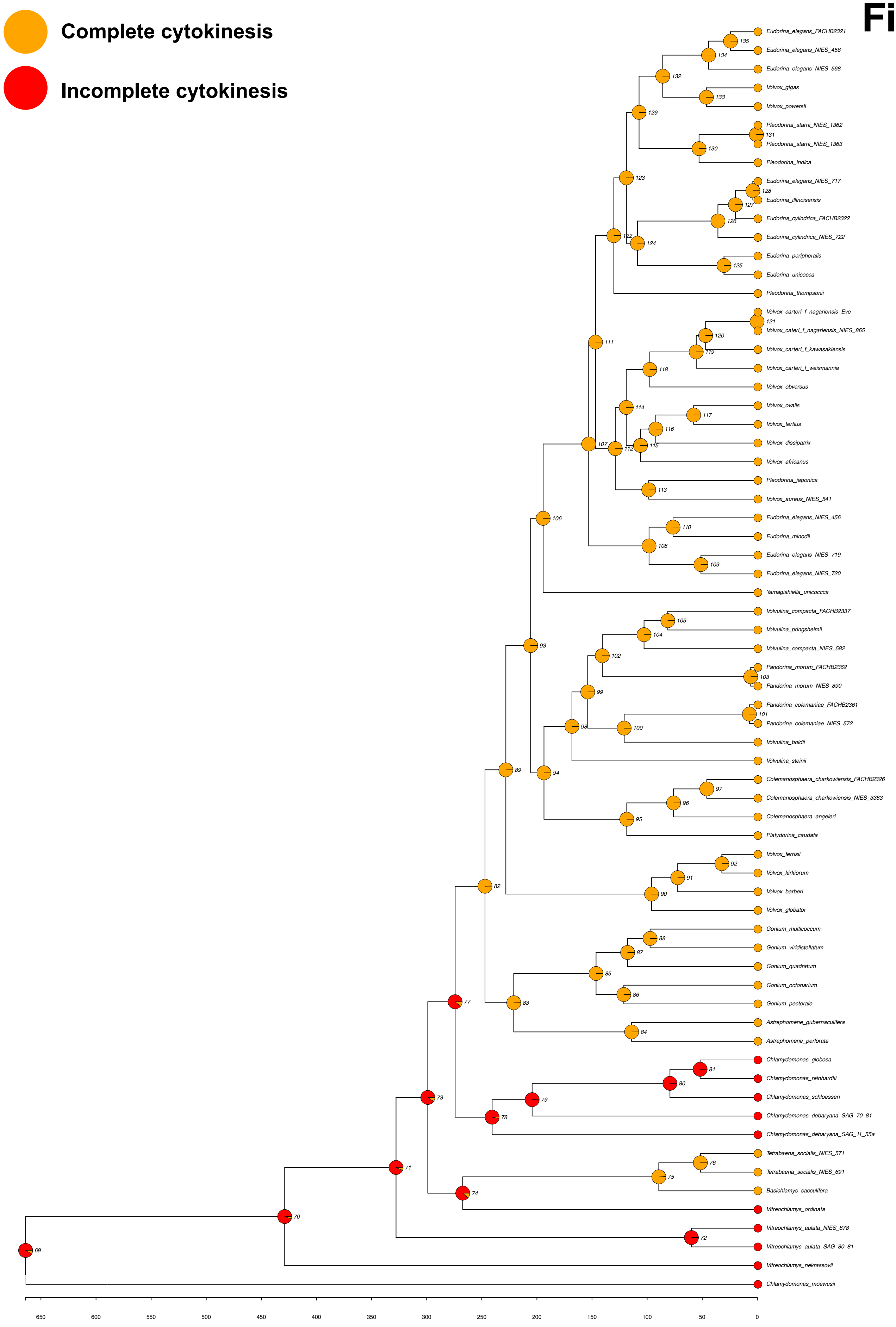
Complete cytokinesis
Incomplete cytokinesis
Eudorina_elegans_FACHB2321
Eudorina_elegans_NIES_458
Eudorina_elegans_NIES_568
Volvox_gigas
Volvox_powersii
Pleodorina_starrii_NIES_1362
Pleodorina_starrii_NIES_1363
Pleodorina_indica
Eudorina_elegans_NIES_717
Eudorina_illinoisensis
Eudorina_cylindrica_FACHB2322
Eudorina_cylindrica_NIES_722
Eudorina_peripheralis
Eudorina_unicocca
Pleodorina_thompsonii
Volvox_carteri_f_nagariensis_Eve
Volvox_cateri_f_nagariensis_NIES_865
Volvox_carteri_f_kawasakiensis
Volvox_carteri_f_weismannia
Volvox_obversus
Volvox_ovalis
Volvox_tertius
Volvox_dissipatrix
Volvox_africanus
Pleodorina_japonica
Volvox_aureus_NIES_541
Eudorina_elegans_NIES_456
Eudorina_minodii
Eudorina_elegans_NIES_719
Eudorina_elegans_NIES_720
Yamagishiella_unicoccca
Volvulina_compacta_FACHB2337
Volvulina_pringsheimii
Volvulina_compacta_NIES_582
Pandorina_morum_FACHB2362
Pandorina_morum_NIES_890
Pandorina_colemaniae_FACHB2361
Pandorina_colemaniae_NIES_572
Volvulina_boldii
Volvulina_steinii
Colemanosphaera_charkowiensis_FACHB2326
Colemanosphaera_charkowiensis_NIES_3383
Colemanosphaera_angeleri
Platydorina_caudata
Volvox_ferrisii
Volvox_kirkiorum
Volvox_barberi
Volvox_globator
Gonium_multicoccum
Gonium_viridistellatum
Gonium_quadratum
Gonium_octonarium
Gonium_pectorale
Astrephomene_gubernaculifera
Astrephomene_perforata
Chlamydomonas_globosa
Chlamydomonas_reinhardtii
Chlamydomonas_schloesseri
Chlamydomonas_debaryana_SAG_70_81
Chlamydomonas_debaryana_SAG_11_55a
Tetrabaena_socialis_NIES_571
Tetrabaena_socialis_NIES_691
Basichlamys_sacculifera
Vitreochlamys_ordinata
Vitreochlamys_aulata_NIES_878
Vitreochlamys_aulata_SAG_80_81
Vitreochlamys_nekrassovii
Chlamydomonas_moewusii
650
600
550
500
450
400
350
300
250
200
150
100
50
0

Fig. S8

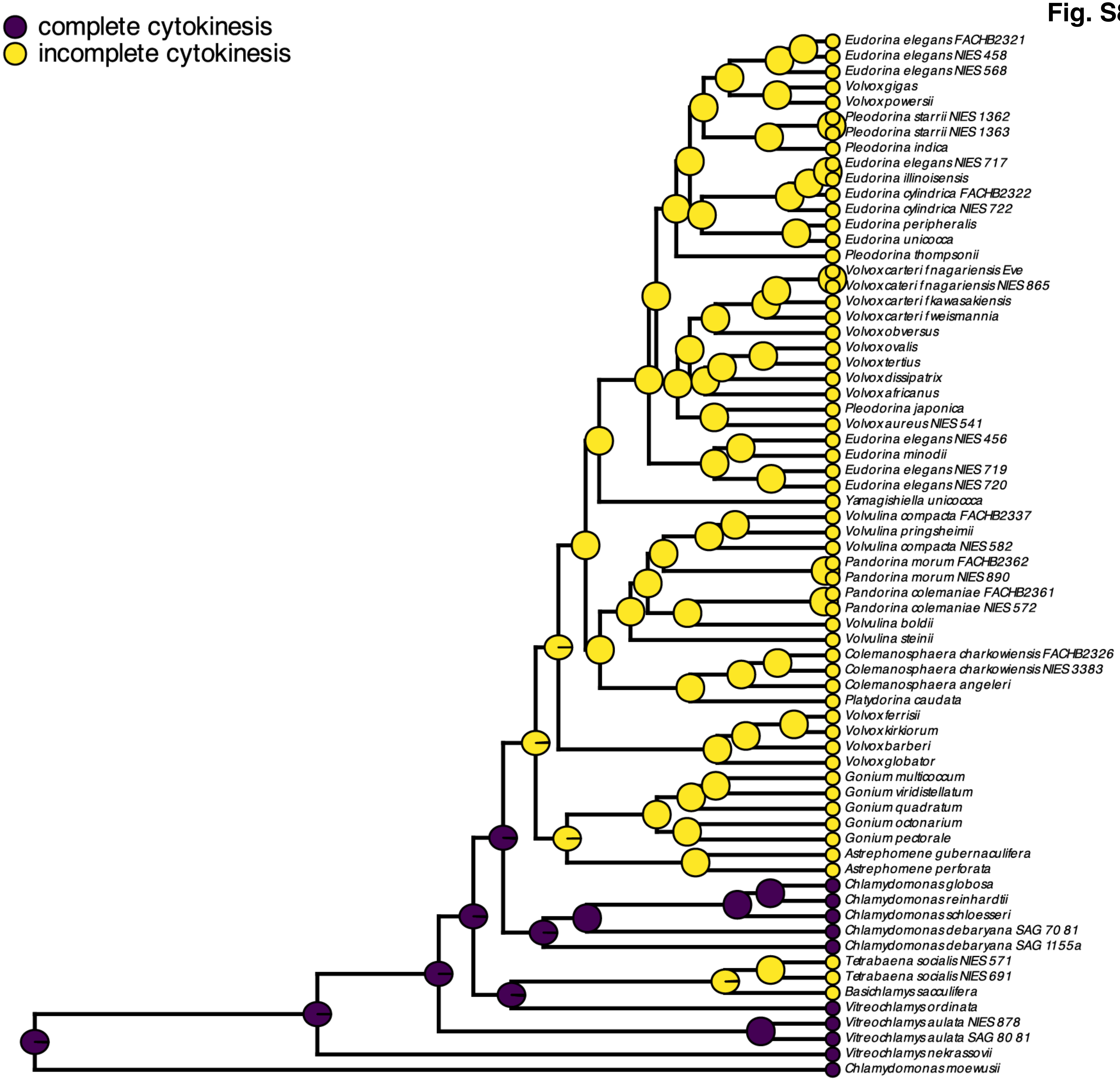
complete cytokinesis
incomplete cytokinesis
Eudorina elegans FACHB2321
Eudorina elegans NIES 458
Eudorina elegans NIES 568
Volvox gigas
Volvox powersii
Pleodorina starrii NIES 1362
Pleodorina starrii NIES 1363
Pleodorina indica
Eudorina elegans NIES 717
Eudorina illinoisensis
Eudorina cylindrica FACHB2322
Eudorina cylindrica NIES 722
Eudorina peripheralis
Eudorina unicocca
Pleodorina thompsonii
Volvox carteri f nagariensis Eve
Volvox cateri f nagariensis NIES 865
Volvox carteri f kawasakiensis
Volvox carteri f weismannia
Volvox obversus
Volvox ovalis
Volvox tertius
Volvox dissipatrix
Volvox africanus
Pleodorina japonica
Volvox aureus NIES 541
Eudorina elegans NIES 456
Eudorina minodii
Eudorina elegans NIES 719
Eudorina elegans NIES 720
Yamagishiella unicocca
Volvulina compacta FACHB2337
Volvulina pringsheimii
Volvulina compacta NIES 582
Pandorina morum FACHB2362
Pandorina morum NIES 890
Pandorina colemaniae FACHB2361
Pandorina colemaniae NIES 572
Volvulina boldii
Volvulina steinii
Colemanosphaera charkowiensis FACHB2326
Colemanosphaera charkowiensis NIES 3383
Colemanosphaera angeleri
Platydorina caudata
Volvox ferrisii
Volvox kirkiorum
Volvox barberi
Volvox globator
Gonium multicoccum
Gonium viridistellatum
Gonium quadratum
Gonium octonarium
Gonium pectorale
Astrephomene gubernaculifera
Astrephomene perforata
Chlamydomonas globosa
Chlamydomonas reinhardtii
Chlamydomonas schloesseri
Chlamydomonas debaryana SAG 70 81
Chlamydomonas debaryana SAG 1155a
Tetrabaena socialis NIES 571
Tetrabaena socialis NIES 691
Basichlamys sacculifera
Vitreochlamys ordinata
Vitreochlamys aulata NIES 878
Vitreochlamys aulata SAG 80 81
Vitreochlamys nekrassovii
Chlamydomonas moewusii

Fig. S9

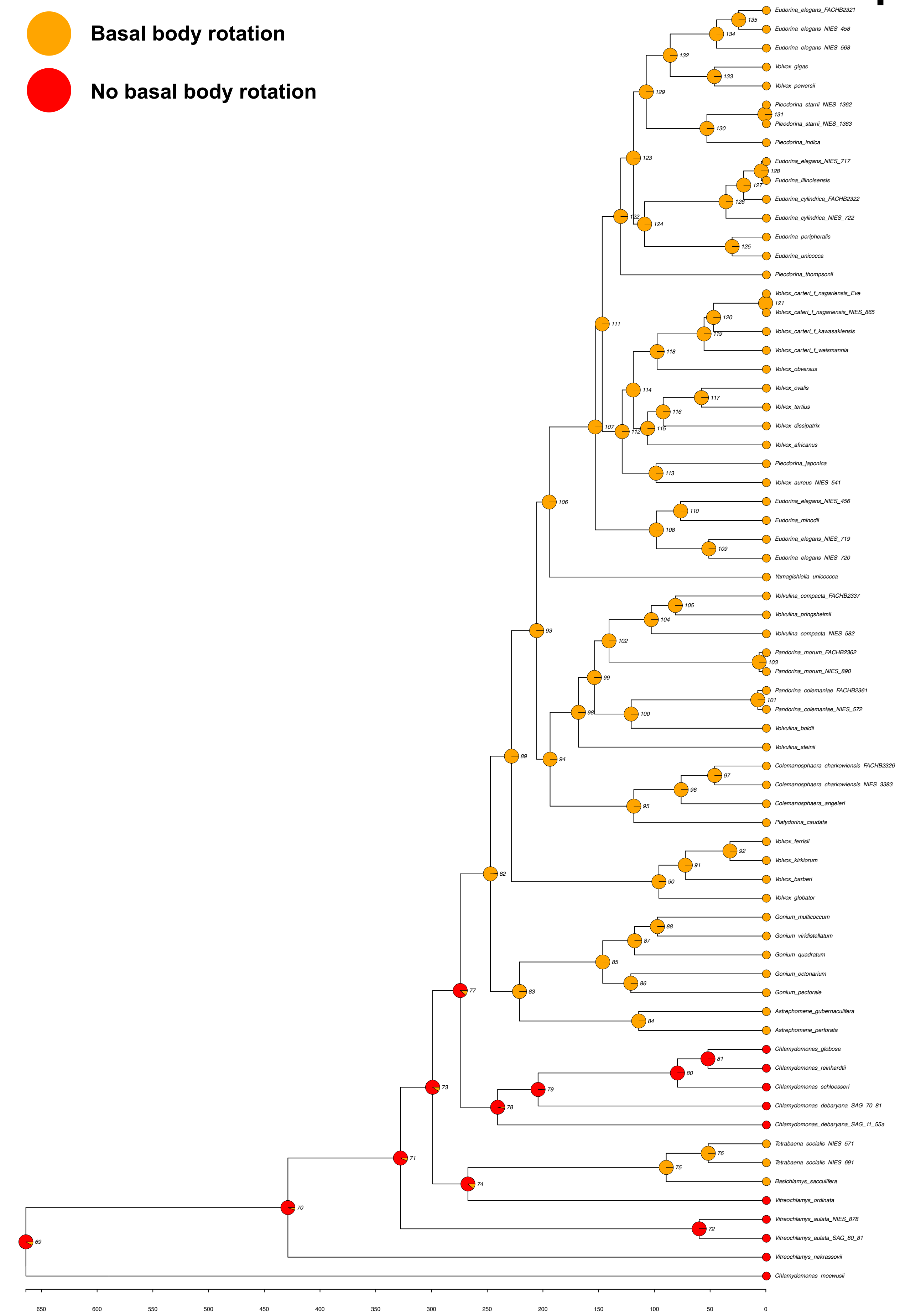

Basal body rotation
No basal body rotation
Eudorina_elegans_FACHB2321
Eudorina_elegans_NIES_458
Eudorina_elegans_NIES_568
Volvox_gigas
Volvox_powersii
Pleodorina_starrii_NIES_1362
Pleodorina_starrii_NIES_1363
Pleodorina_indica
Eudorina_elegans_NIES_717
Eudorina_illinoisensis
Eudorina_cylindrica_FACHB2322
Eudorina_cylindrica_NIES_722
Eudorina_peripheralis
Eudorina_unicocca
Pleodorina_thompsonii
Volvox_carteri_f_nagariensis_Eve
Volvox_cateri_f_nagariensis_NIES_865
Volvox_carteri_f_kawasakiensis
Volvox_carteri_f_weismannia
Volvox_obversus
Volvox_ovalis
Volvox_tertius
Volvox_dissipatrix
Volvox_africanus
Pleodorina_japonica
Volvox_aureus_NIES_541
Eudorina_elegans_NIES_456
Eudorina_minodii
Eudorina_elegans_NIES_719
Eudorina_elegans_NIES_720
Yamagishiella_unicoccca
Volvulina_compacta_FACHB2337
Volvulina_pringsheimii
Volvulina_compacta_NIES_582
Pandorina_morum_FACHB2362
Pandorina_morum_NIES_890
Pandorina_colemaniae_FACHB2361
Pandorina_colemaniae_NIES_572
Volvulina_boldii
Volvulina_steinii
Colemanosphaera_charkowiensis_FACHB2326
Colemanosphaera_charkowiensis_NIES_3383
Colemanosphaera_angeleri
Platydorina_caudata
Volvox_ferrisii
Volvox_kirkiorum
Volvox_barberi
Volvox_globator
Gonium_multicoccum
Gonium_viridistellatum
Gonium_quadratum
Gonium_octonarium
Gonium_pectorale
Astrephomene_gubernaculifera
Astrephomene_perforata
Chlamydomonas_globosa
Chlamydomonas_reinhardtii
Chlamydomonas_schloesseri
Chlamydomonas_debaryana_SAG_70_81
Chlamydomonas_debaryana_SAG_11_55a
Tetrabaena_socialis_NIES_571
Tetrabaena_socialis_NIES_691
Basichlamys_sacculifera
Vitreochlamys_ordinata
Vitreochlamys_aulata_NIES_878
Vitreochlamys_aulata_SAG_80_81
Vitreochlamys_nekrassovii
Chlamydomonas_moewusii
650 600 550 500 450 400 350 300 250 200 150 100 50 0

Fig. S10

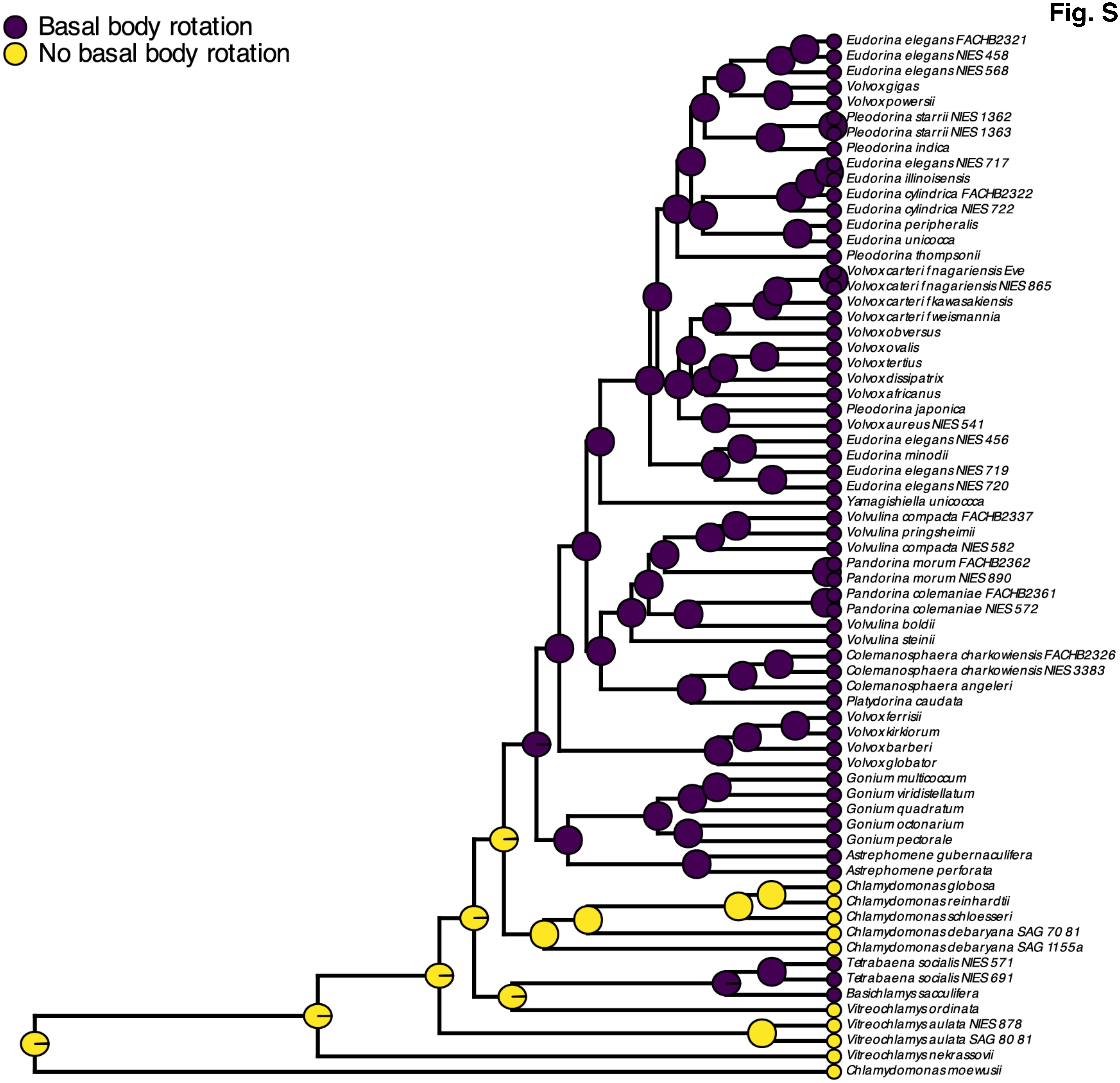

Basal body rotation
No basal body rotation
Eudorina elegans FACHB2321
Eudorina elegans NIES 458
Eudorina elegans NIES 568
Volvox gigas
Volvox powersii
Pleodorina starrii NIES 1362
Pleodorina starrii NIES 1363
Pleodorina indica
Eudorina elegans NIES 717
Eudorina illinoisensis
Eudorina cylindrica FACHB2322
Eudorina cylindrica NIES 722
Eudorina peripheralis
Eudorina unicocca
Pleodorina thompsonii
Volvox carteri f nagariensis Eve
Volvox cateri f nagariensis NIES 865
Volvox carteri f kawasakiensis
Volvox carteri f weismannia
Volvox obversus
Volvox ovalis
Volvox tertius
Volvox dissipatrix
Volvox africanus
Pleodorina japonica
Volvox aureus NIES 541
Eudorina elegans NIES 456
Eudorina minodii
Eudorina elegans NIES 719
Eudorina elegans NIES 720
Yamagishiella unicocca
Volvulina compacta FACHB2337
Volvulina pringsheimii
Volvulina compacta NIES 582
Pandorina morum FACHB2362
Pandorina morum NIES 890
Pandorina colemaniae FACHB2361
Pandorina colemaniae NIES 572
Volvulina boldii
Volvulina steinii
Colemanosphaera charkowiensis FACHB2326
Colemanosphaera charkowiensis NIES 3383
Colemanosphaera angeleri
Platydorina caudata
Volvox ferrisii
Volvox kirkiorum
Volvox barberi
Volvox globator
Gonium multicoccum
Gonium viridistellatum
Gonium quadratum
Gonium octonarium
Gonium pectorale
Astrephomene gubernaculifera
Astrephomene perforata
Chlamydomonas globosa
Chlamydomonas reinhardtii
Chlamydomonas schloesseri
Chlamydomonas debaryana SAG 70 81
Chlamydomonas debaryana SAG 1155a
Tetrabaena socialis NIES 571
Tetrabaena socialis NIES 691
Basichlamys sacculifera
Vitreochlamys ordinata
Vitreochlamys aulata NIES 878
Vitreochlamys aulata SAG 80 81
Vitreochlamys nekrassovii
Chlamydomonas moewusii

Fig. S11

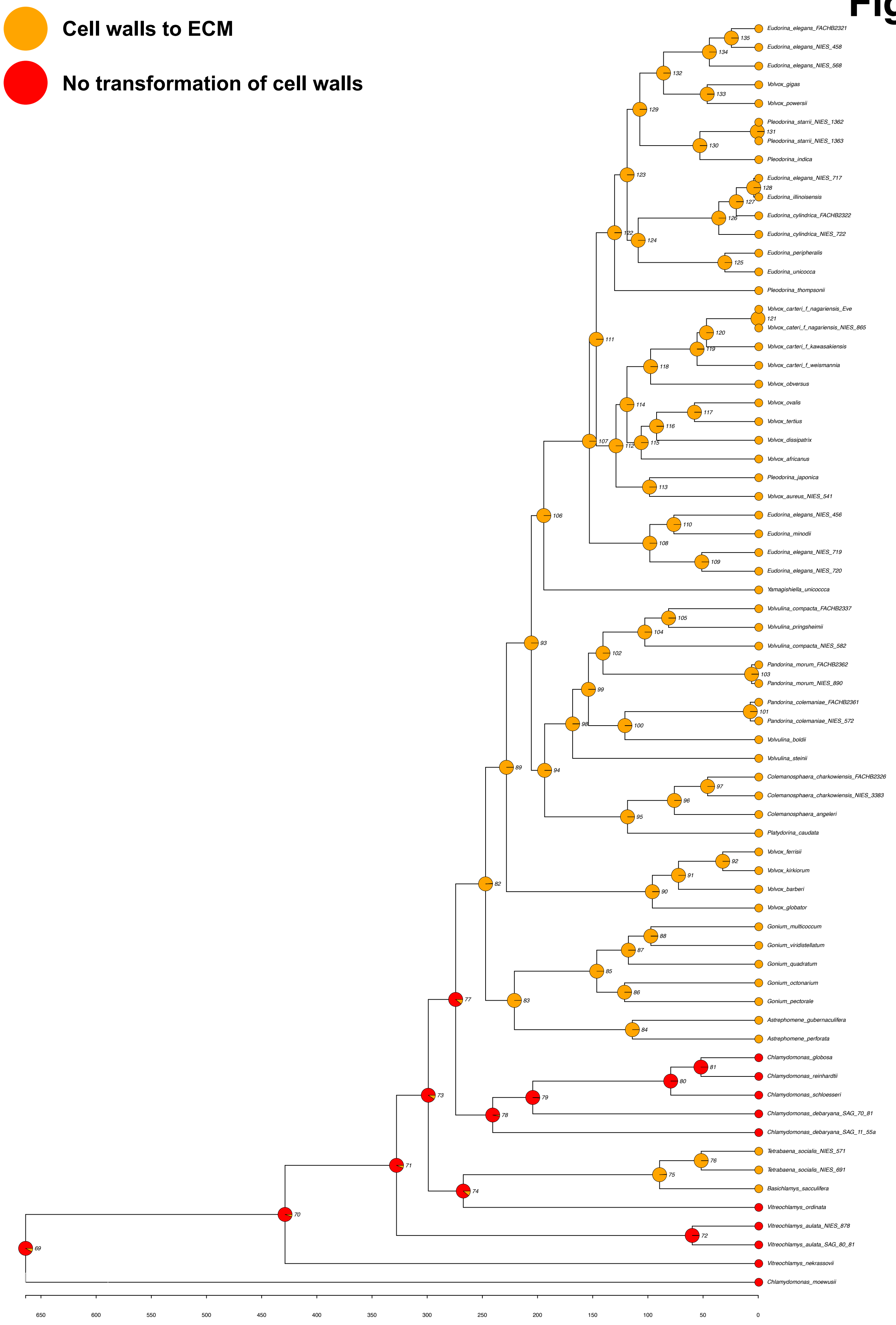

Cell walls to ECM
No transformation of cell walls
Eudorina_elegans_FACHB2321
Eudorina_elegans_NIES_458
Eudorina_elegans_NIES_568
Volvox_gigas
Volvox_powersii
Pleodorina_starrii_NIES_1362
Pleodorina_starrii_NIES_1363
Pleodorina_indica
Eudorina_elegans_NIES_717
Eudorina_illinoisensis
Eudorina_cylindrica_FACHB2322
Eudorina_cylindrica_NIES_722
Eudorina_peripheralis
Eudorina_unicocca
Pleodorina_thompsonii
Volvox_carteri_f_nagariensis_Eve
Volvox_cateri_f_nagariensis_NIES_865
Volvox_carteri_f_kawasakiensis
Volvox_carteri_f_weismannia
Volvox_obversus
Volvox_ovalis
Volvox_tertius
Volvox_dissipatrix
Volvox_africanus
Pleodorina_japonica
Volvox_aureus_NIES_541
Eudorina_elegans_NIES_456
Eudorina_minodii
Eudorina_elegans_NIES_719
Eudorina_elegans_NIES_720
Yamagishiella_unicoccca
Volvulina_compacta_FACHB2337
Volvulina_pringsheimii
Volvulina_compacta_NIES_582
Pandorina_morum_FACHB2362
Pandorina_morum_NIES_890
Pandorina_colemaniae_FACHB2361
Pandorina_colemaniae_NIES_572
Volvulina_boldii
Volvulina_steinii
Colemanosphaera_charkowiensis_FACHB2326
Colemanosphaera_charkowiensis_NIES_3383
Colemanosphaera_angeleri
Platydorina_caudata
Volvox_ferrisii
Volvox_kirkiorum
Volvox_barberi
Volvox_globator
Gonium_multicoccum
Gonium_viridistellatum
Gonium_quadratum
Gonium_octonarium
Gonium_pectorale
Astrephomene_gubernaculifera
Astrephomene_perforata
Chlamydomonas_globosa
Chlamydomonas_reinhardtii
Chlamydomonas_schloesseri
Chlamydomonas_debaryana_SAG_70_81
Chlamydomonas_debaryana_SAG_11_55a
Tetrabaena_socialis_NIES_571
Tetrabaena_socialis_NIES_691
Basichlamys_sacculifera
Vitreochlamys_ordinata
Vitreochlamys_aulata_NIES_878
Vitreochlamys_aulata_SAG_80_81
Vitreochlamys_nekrassovii
Chlamydomonas_moewusii
650
600
550
500
450
400
350
300
250
200
150
100
50
0

Fig. S12

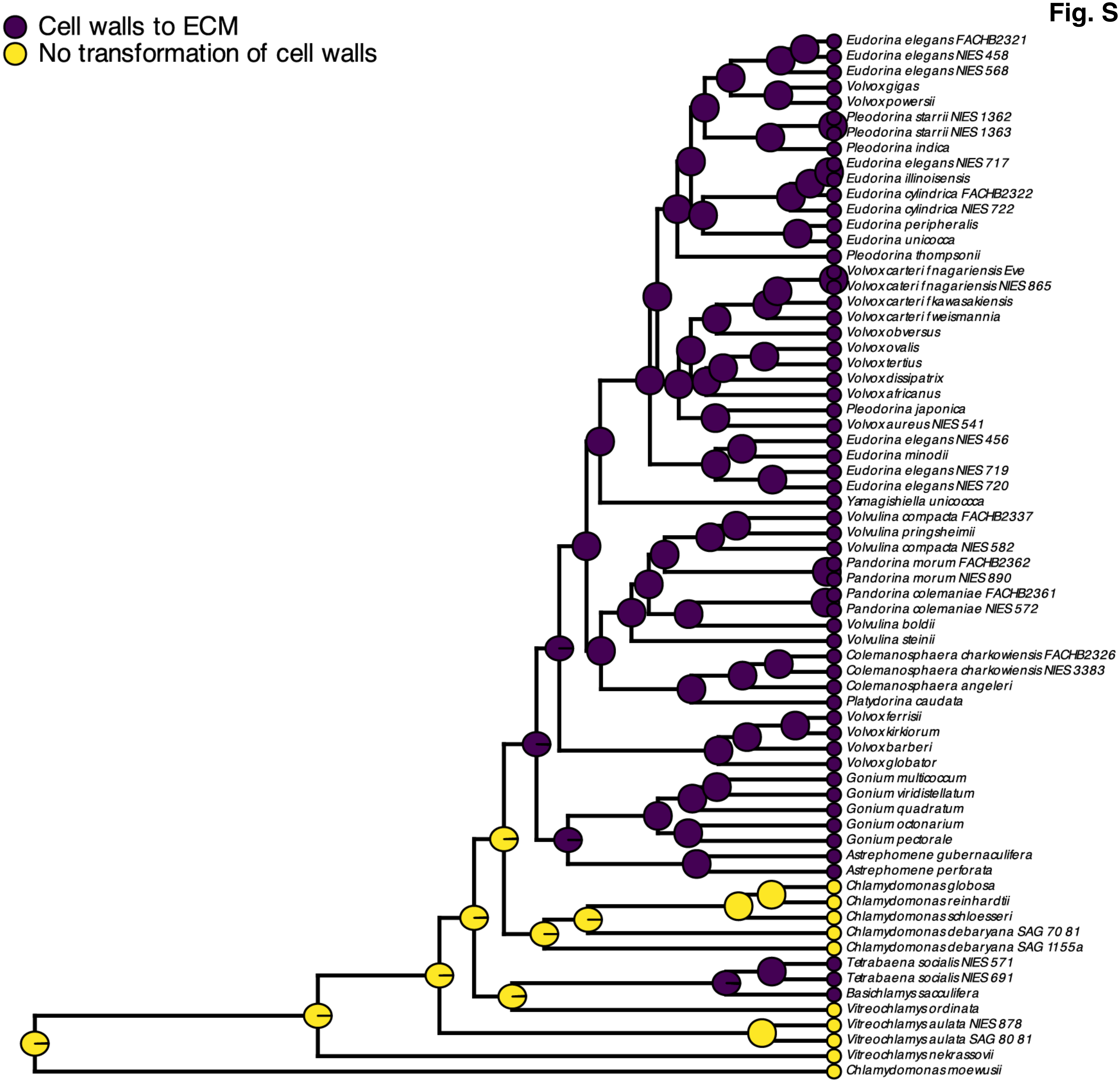
Cell walls to ECM
No transformation of cell walls
Eudorina elegans FACHB2321
Eudorina elegans NIES 458
Eudorina elegans NIES 568
Volvox gigas
Volvox powersii
Pleodorina starrii NIES 1362
Pleodorina starrii NIES 1363
Pleodorina indica
Eudorina elegans NIES 717
Eudorina illinoisensis
Eudorina cylindrica FACHB2322
Eudorina cylindrica NIES 722
Eudorina peripheralis
Eudorina unicocca
Pleodorina thompsonii
Volvox carteri f nagariensis Eve
Volvox cateri f nagariensis NIES 865
Volvox carteri f kawasakiensis
Volvox carteri f weismannia
Volvox obversus
Volvox ovalis
Volvox tertius
Volvox dissipatrix
Volvox africanus
Pleodorina japonica
Volvox aureus NIES 541
Eudorina elegans NIES 456
Eudorina minodii
Eudorina elegans NIES 719
Eudorina elegans NIES 720
Yamagishiella unicocca
Volvulina compacta FACHB2337
Volvulina pringsheimii
Volvulina compacta NIES 582
Pandorina morum FACHB2362
Pandorina morum NIES 890
Pandorina colemaniae FACHB2361
Pandorina colemaniae NIES 572
Volvulina boldii
Volvulina steinii
Colemanosphaera charkowiensis FACHB2326
Colemanosphaera charkowiensis NIES 3383
Colemanosphaera angeleri
Platydorina caudata
Volvox ferrisii
Volvox kirkiorum
Volvox barberi
Volvox globator
Gonium multicoccum
Gonium viridistellatum
Gonium quadratum
Gonium octonarium
Gonium pectorale
Astrephomene gubernaculifera
Astrephomene perforata
Chlamydomonas globosa
Chlamydomonas reinhardtii
Chlamydomonas schloesseri
Chlamydomonas debaryana SAG 70 81
Chlamydomonas debaryana SAG 1155a
Tetrabaena socialis NIES 571
Tetrabaena socialis NIES 691
Basichlamys sacculifera
Vitreochlamys ordinata
Vitreochlamys aulata NIES 878
Vitreochlamys aulata SAG 80 81
Vitreochlamys nekrassovii
Chlamydomonas moewusii

Fig. S13

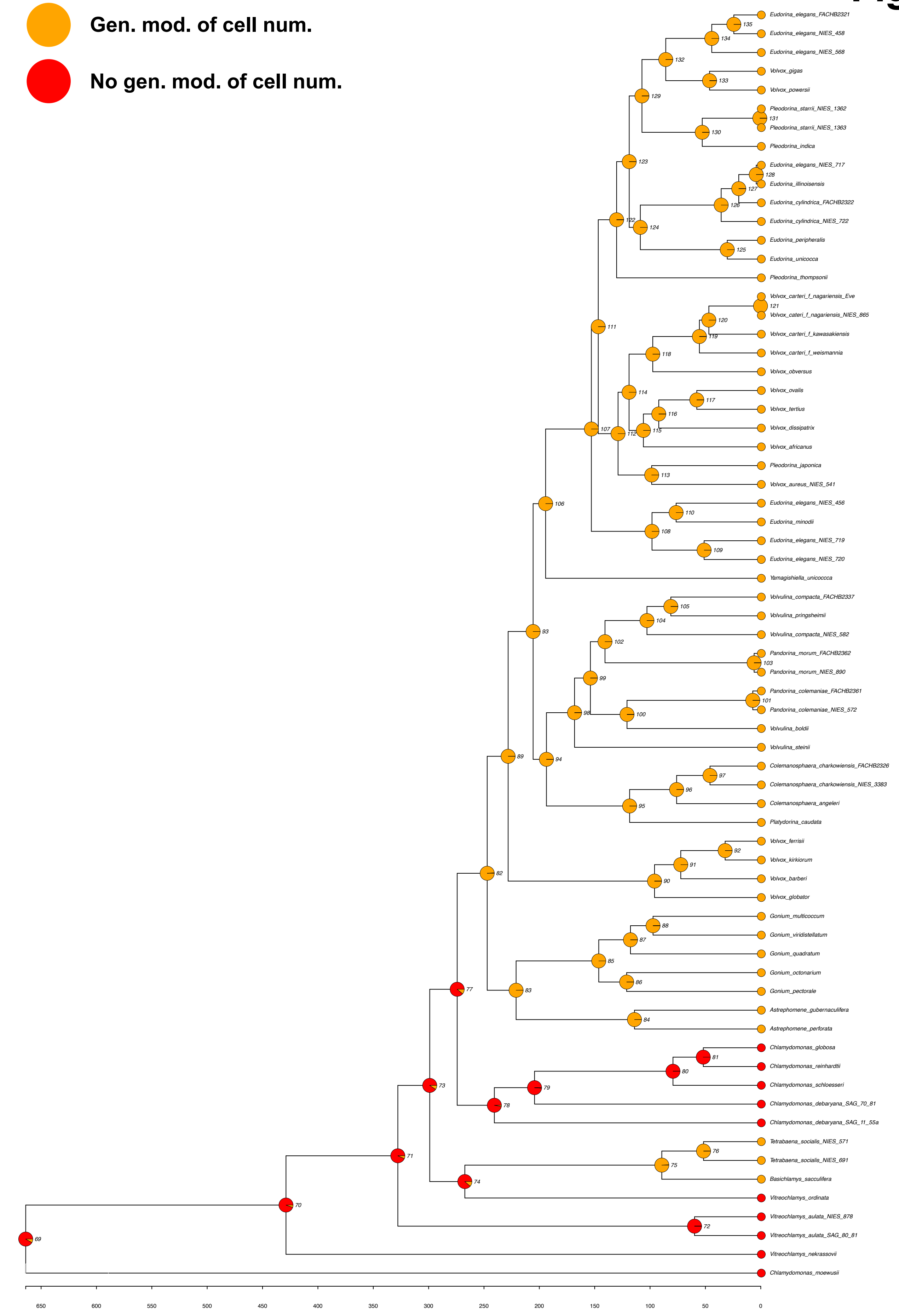
Gen. mod. of cell num.
No gen. mod. of cell num.
Eudorina_elegans_FACHB2321
Eudorina_elegans_NIES_458
Eudorina_elegans_NIES_568
Volvox_gigas
Volvox_powersii
Pleodorina_starrii_NIES_1362
Pleodorina_starrii_NIES_1363
Pleodorina_indica
Eudorina_elegans_NIES_717
Eudorina_illinoisensis
Eudorina_cylindrica_FACHB2322
Eudorina_cylindrica_NIES_722
Eudorina_peripheralis
Eudorina_unicocca
Pleodorina_thompsonii
Volvox_carteri_f_nagariensis_Eve
Volvox_cateri_f_nagariensis_NIES_865
Volvox_carteri_f_kawasakiensis
Volvox_carteri_f_weismannia
Volvox_obversus
Volvox_ovalis
Volvox_tertius
Volvox_dissipatrix
Volvox_africanus
Pleodorina_japonica
Volvox_aureus_NIES_541
Eudorina_elegans_NIES_456
Eudorina_minodii
Eudorina_elegans_NIES_719
Eudorina_elegans_NIES_720
Yamagishiella_unicoccca
Volvulina_compacta_FACHB2337
Volvulina_pringsheimii
Volvulina_compacta_NIES_582
Pandorina_morum_FACHB2362
Pandorina_morum_NIES_890
Pandorina_colemaniae_FACHB2361
Pandorina_colemaniae_NIES_572
Volvulina_boldii
Volvulina_steinii
Colemanosphaera_charkowiensis_FACHB2326
Colemanosphaera_charkowiensis_NIES_3383
Colemanosphaera_angeleri
Platydorina_caudata
Volvox_ferrisii
Volvox_kirkiorum
Volvox_barberi
Volvox_globator
Gonium_multicoccum
Gonium_viridistellatum
Gonium_quadratum
Gonium_octonarium
Gonium_pectorale
Astrephomene_gubernaculifera
Astrephomene_perforata
Chlamydomonas_globosa
Chlamydomonas_reinhardtii
Chlamydomonas_schloesseri
Chlamydomonas_debaryana_SAG_70_81
Chlamydomonas_debaryana_SAG_11_55a
Tetrabaena_socialis_NIES_571
Tetrabaena_socialis_NIES_691
Basichlamys_sacculifera
Vitreochlamys_ordinata
Vitreochlamys_aulata_NIES_878
Vitreochlamys_aulata_SAG_80_81
Vitreochlamys_nekrassovii
Chlamydomonas_moewusii
650
600
550
500
450
400
350
300
250
200
150
100
50
0

Fig. S14

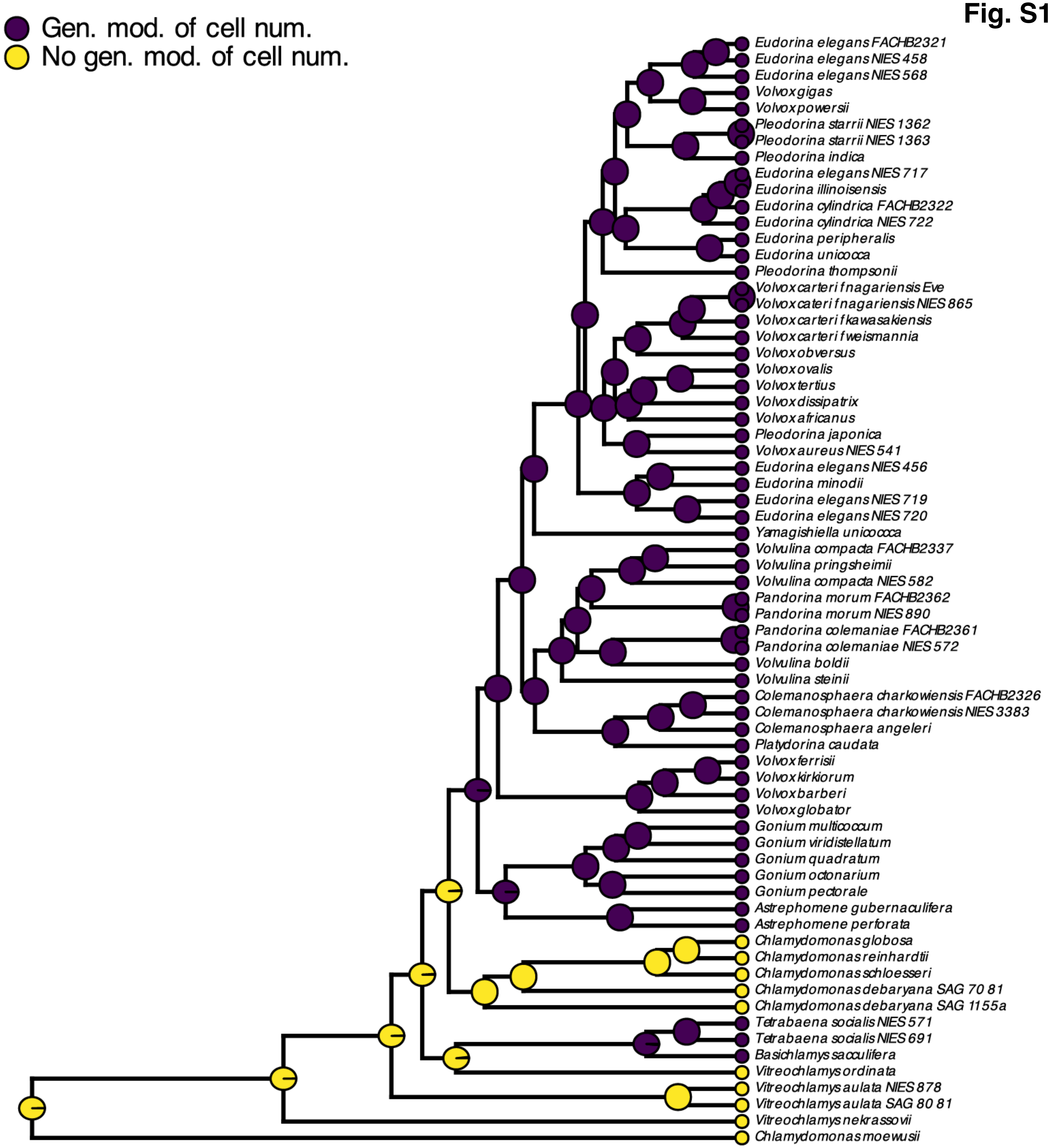

Gen. mod. of cell num.
No gen. mod. of cell num.
Eudorina elegans FACHB2321
Eudorina elegans NIES 458
Eudorina elegans NIES 568
Volvox gigas
Volvox powersii
Pleodorina starrii NIES 1362
Pleodorina starrii NIES 1363
Pleodorina indica
Eudorina elegans NIES 717
Eudorina illinoisensis
Eudorina cylindrica FACHB2322
Eudorina cylindrica NIES 722
Eudorina peripheralis
Eudorina unicocca
Pleodorina thompsonii
Volvox carteri f nagariensis Eve
Volvox cateri f nagariensis NIES 865
Volvox carteri f kawasakiensis
Volvox carteri f weismannia
Volvox obversus
Volvox ovalis
Volvox tertius
Volvox dissipatrix
Volvox africanus
Pleodorina japonica
Volvox aureus NIES 541
Eudorina elegans NIES 456
Eudorina minodii
Eudorina elegans NIES 719
Eudorina elegans NIES 720
Yamagishiella unicocca
Volvulina compacta FACHB2337
Volvulina pringsheimii
Volvulina compacta NIES 582
Pandorina morum FACHB2362
Pandorina morum NIES 890
Pandorina colemaniae FACHB2361
Pandorina colemaniae NIES 572
Volvulina boldii
Volvulina steinii
Colemanosphaera charkowiensis FACHB2326
Colemanosphaera charkowiensis NIES 3383
Colemanosphaera angeleri
Platydorina caudata
Volvox ferrisii
Volvox kirkiorum
Volvox barberi
Volvox globator
Gonium multicoccum
Gonium viridistellatum
Gonium quadratum
Gonium octonarium
Gonium pectorale
Astrephomene gubernaculifera
Astrephomene perforata
Chlamydomonas globosa
Chlamydomonas reinhardtii
Chlamydomonas schloesseri
Chlamydomonas debaryana SAG 70 81
Chlamydomonas debaryana SAG 1155a
Tetrabaena socialis NIES 571
Tetrabaena socialis NIES 691
Basichlamys sacculifera
Vitreochlamys ordinata
Vitreochlamys aulata NIES 878
Vitreochlamys aulata SAG 80 81
Vitreochlamys nekrassovii
Chlamydomonas moewusii

Fig. S15

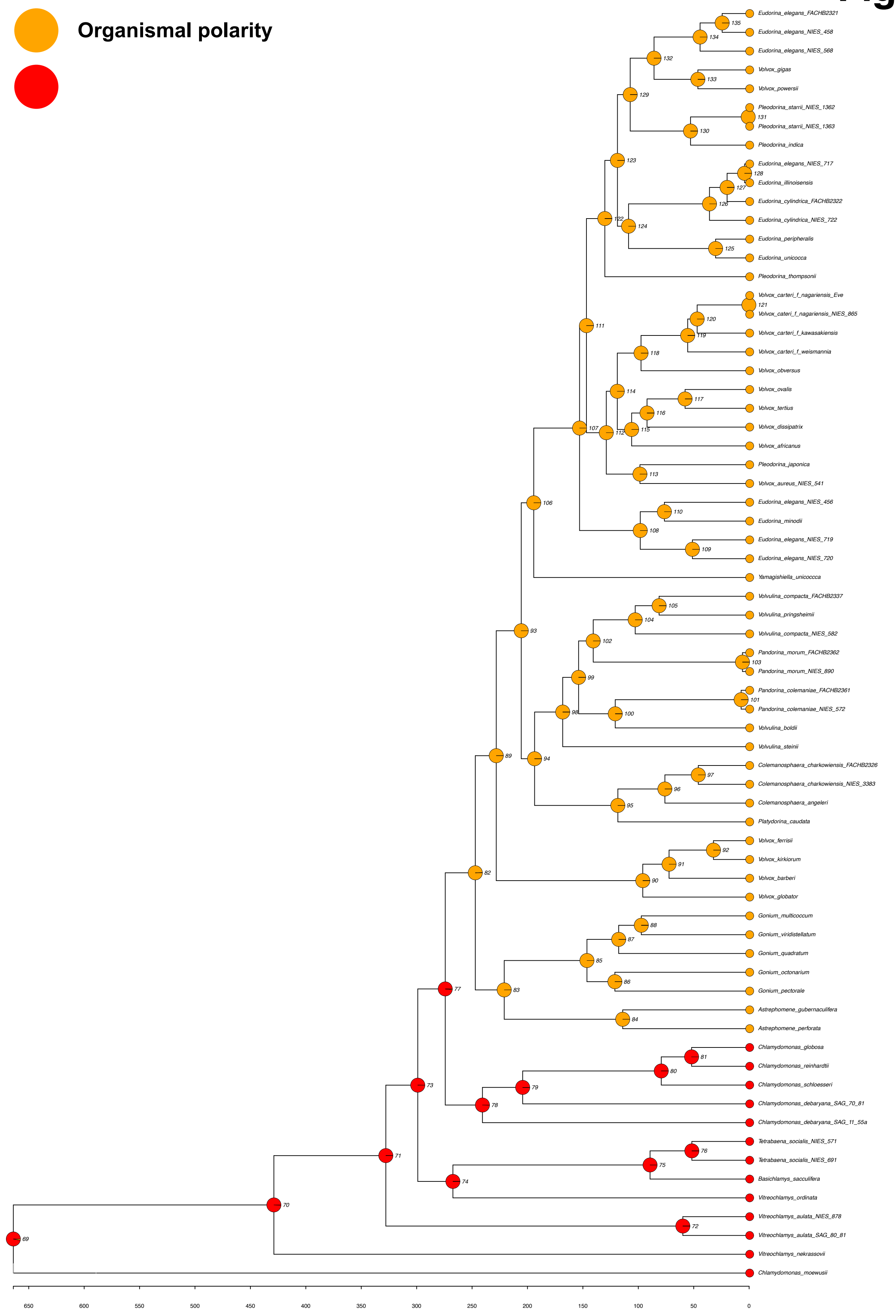

Organismal polarity
Eudorina_elegans_FACHB2321
Eudorina_elegans_NIES_458
Eudorina_elegans_NIES_568
Volvox_gigas
Volvox_powersii
Pleodorina_starrii_NIES_1362
Pleodorina_starrii_NIES_1363
Pleodorina_indica
Eudorina_elegans_NIES_717
Eudorina_illinoisensis
Eudorina_cylindrica_FACHB2322
Eudorina_cylindrica_NIES_722
Eudorina_peripheralis
Eudorina_unicocca
Pleodorina_thompsonii
Volvox_carteri_f_nagariensis_Eve
Volvox_cateri_f_nagariensis_NIES_865
Volvox_carteri_f_kawasakiensis
Volvox_carteri_f_weismannia
Volvox_obversus
Volvox_ovalis
Volvox_tertius
Volvox_dissipatrix
Volvox_africanus
Pleodorina_japonica
Volvox_aureus_NIES_541
Eudorina_elegans_NIES_456
Eudorina_minodii
Eudorina_elegans_NIES_719
Eudorina_elegans_NIES_720
Yamagishiella_unicoccca
Volvulina_compacta_FACHB2337
Volvulina_pringsheimii
Volvulina_compacta_NIES_582
Pandorina_morum_FACHB2362
Pandorina_morum_NIES_890
Pandorina_colemaniae_FACHB2361
Pandorina_colemaniae_NIES_572
Volvulina_boldii
Volvulina_steinii
Colemanosphaera_charkowiensis_FACHB2326
Colemanosphaera_charkowiensis_NIES_3383
Colemanosphaera_angeleri
Platydorina_caudata
Volvox_ferrisii
Volvox_kirkiorum
Volvox_barberi
Volvox_globator
Gonium_multicoccum
Gonium_viridistellatum
Gonium_quadratum
Gonium_octonarium
Gonium_pectorale
Astrephomene_gubernaculifera
Astrephomene_perforata
Chlamydomonas_globosa
Chlamydomonas_reinhardtii
Chlamydomonas_schloesseri
Chlamydomonas_debaryana_SAG_70_81
Chlamydomonas_debaryana_SAG_11_55a
Tetrabaena_socialis_NIES_571
Tetrabaena_socialis_NIES_691
Basichlamys_sacculifera
Vitreochlamys_ordinata
Vitreochlamys_aulata_NIES_878
Vitreochlamys_aulata_SAG_80_81
Vitreochlamys_nekrassovii
Chlamydomonas_moewusii
650
600
550
500
450
400
350
300
250
200
150
100
50
0

Fig. S16

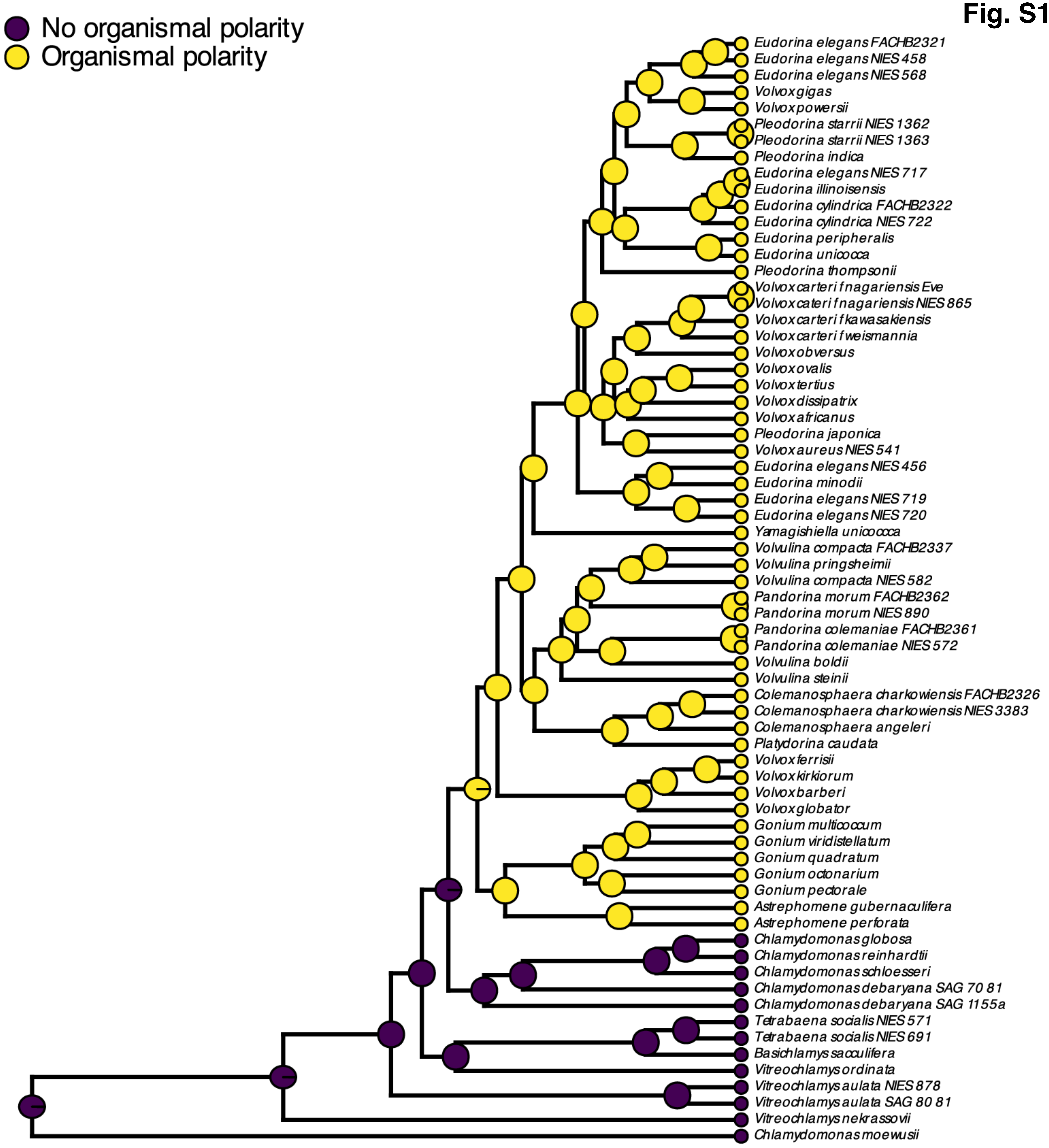

No organismal polarity
Organismal polarity
Eudorina elegans FACHB2321
Eudorina elegans NIES 458
Eudorina elegans NIES 568
Volvox gigas
Volvox powersii
Pleodorina starrii NIES 1362
Pleodorina starrii NIES 1363
Pleodorina indica
Eudorina elegans NIES 717
Eudorina illinoisensis
Eudorina cylindrica FACHB2322
Eudorina cylindrica NIES 722
Eudorina peripheralis
Eudorina unicocca
Pleodorina thompsonii
Volvox carteri f nagariensis Eve
Volvox cateri f nagariensis NIES 865
Volvox carteri f kawasakiensis
Volvox carteri f weismannia
Volvox obversus
Volvox ovalis
Volvox tertius
Volvox dissipatrix
Volvox africanus
Pleodorina japonica
Volvox aureus NIES 541
Eudorina elegans NIES 456
Eudorina minodii
Eudorina elegans NIES 719
Eudorina elegans NIES 720
Yamagishiella unicocca
Volvulina compacta FACHB2337
Volvulina pringsheimii
Volvulina compacta NIES 582
Pandorina morum FACHB2362
Pandorina morum NIES 890
Pandorina colemaniae FACHB2361
Pandorina colemaniae NIES 572
Volvulina boldii
Volvulina steinii
Colemanosphaera charkowiensis FACHB2326
Colemanosphaera charkowiensis NIES 3383
Colemanosphaera angeleri
Platydorina caudata
Volvox ferrisii
Volvox kirkiorum
Volvox barberi
Volvox globator
Gonium multicoccum
Gonium viridistellatum
Gonium quadratum
Gonium octonarium
Gonium pectorale
Astrephomene gubernaculifera
Astrephomene perforata
Chlamydomonas globosa
Chlamydomonas reinhardtii
Chlamydomonas schloesseri
Chlamydomonas debaryana SAG 70 81
Chlamydomonas debaryana SAG 1155a
Tetrabaena socialis NIES 571
Tetrabaena socialis NIES 691
Basichlamys sacculifera
Vitreochlamys ordinata
Vitreochlamys aulata NIES 878
Vitreochlamys aulata SAG 80 81
Vitreochlamys nekrassovii
Chlamydomonas moewusii

Fig. S17

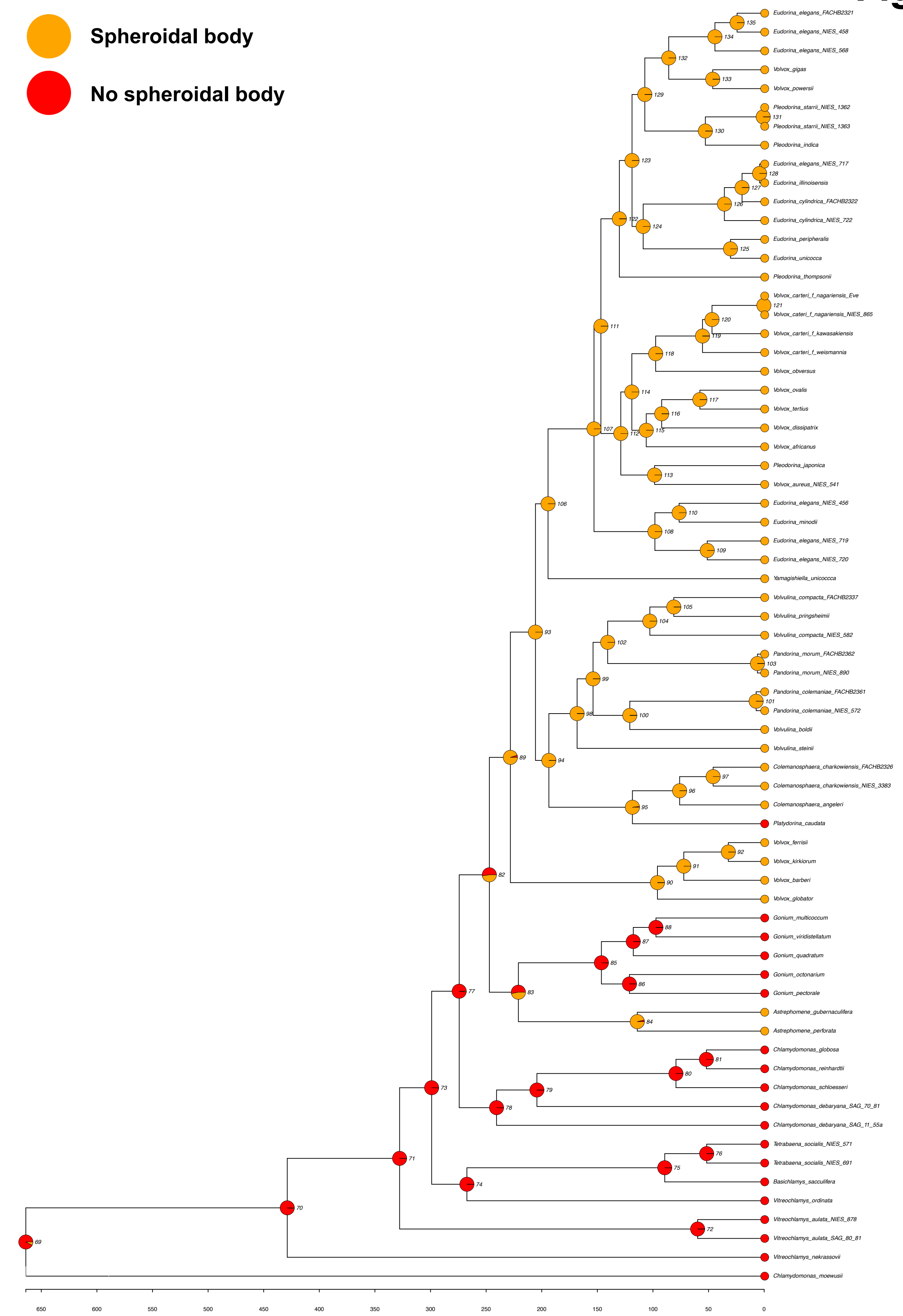
Spheroidal body
No spheroidal body
Eudorina_elegans_FACHB2321
Eudorina_elegans_NIES_458
Eudorina_elegans_NIES_568
Volvox_gigas
Volvox_powersii
Pleodorina_starrii_NIES_1362
Pleodorina_starrii_NIES_1363
Pleodorina_indica
Eudorina_elegans_NIES_717
Eudorina_illinoisensis
Eudorina_cylindrica_FACHB2322
Eudorina_cylindrica_NIES_722
Eudorina_peripheralis
Eudorina_unicocca
Pleodorina_thompsonii
Volvox_carteri_f_nagariensis_Eve
Volvox_cateri_f_nagariensis_NIES_865
Volvox_carteri_f_kawasakiensis
Volvox_carteri_f_weismannia
Volvox_obversus
Volvox_ovalis
Volvox_tertius
Volvox_dissipatrix
Volvox_africanus
Pleodorina_japonica
Volvox_aureus_NIES_541
Eudorina_elegans_NIES_456
Eudorina_minodii
Eudorina_elegans_NIES_719
Eudorina_elegans_NIES_720
Yamagishiella_unicoccca
Volvulina_compacta_FACHB2337
Volvulina_pringsheimii
Volvulina_compacta_NIES_582
Pandorina_morum_FACHB2362
Pandorina_morum_NIES_890
Pandorina_colemaniae_FACHB2361
Pandorina_colemaniae_NIES_572
Volvulina_boldii
Volvulina_steinii
Colemanosphaera_charkowiensis_FACHB2326
Colemanosphaera_charkowiensis_NIES_3383
Colemanosphaera_angeleri
Platydorina_caudata
Volvox_ferrisii
Volvox_kirkiorum
Volvox_barberi
Volvox_globator
Gonium_multicoccum
Gonium_viridistellatum
Gonium_quadratum
Gonium_octonarium
Gonium_pectorale
Astrephomene_gubernaculifera
Astrephomene_perforata
Chlamydomonas_globosa
Chlamydomonas_reinhardtii
Chlamydomonas_schloesseri
Chlamydomonas_debaryana_SAG_70_81
Chlamydomonas_debaryana_SAG_11_55a
Tetrabaena_socialis_NIES_571
Tetrabaena_socialis_NIES_691
Basichlamys_sacculifera
Vitreochlamys_ordinata
Vitreochlamys_aulata_NIES_878
Vitreochlamys_aulata_SAG_80_81
Vitreochlamys_nekrassovii
Chlamydomonas_moewusii
650
600
550
500
450
400
350
300
250
200
150
100
50
0

Fig. S18

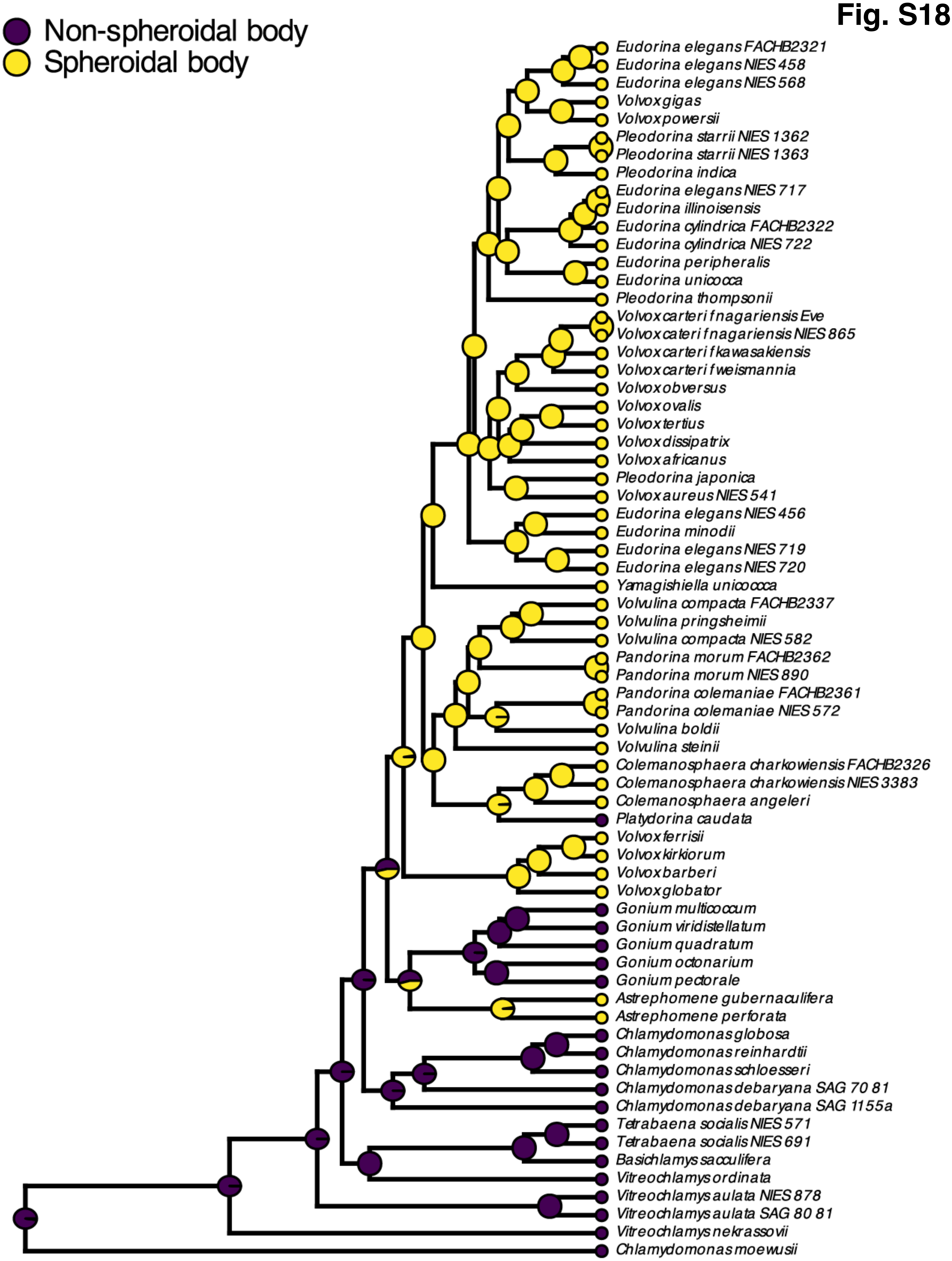
Non-spheroidal body
Spheroidal body
Eudorina elegans FACHB2321
Eudorina elegans NIES 458
Eudorina elegans NIES 568
Volvox gigas
Volvox powersii
Pleodorina starrii NIES 1362
Pleodorina starrii NIES 1363
Pleodorina indica
Eudorina elegans NIES 717
Eudorina illinoisensis
Eudorina cylindrica FACHB2322
Eudorina cylindrica NIES 722
Eudorina peripheralis
Eudorina unicocca
Pleodorina thompsonii
Volvox carteri f nagariensis Eve
Volvox cateri f nagariensis NIES 865
Volvox carteri f kawasakiensis
Volvox carteri f weismannia
Volvox obversus
Volvox ovalis
Volvox tertius
Volvox dissipatrix
Volvox africanus
Pleodorina japonica
Volvox aureus NIES 541
Eudorina elegans NIES 456
Eudorina minodii
Eudorina elegans NIES 719
Eudorina elegans NIES 720
Yamagishiella unicocca
Volvulina compacta FACHB2337
Volvulina pringsheimii
Volvulina compacta NIES 582
Pandorina morum FACHB2362
Pandorina morum NIES 890
Pandorina colemaniae FACHB2361
Pandorina colemaniae NIES 572
Volvulina boldii
Volvulina steinii
Colemanosphaera charkowiensis FACHB2326
Colemanosphaera charkowiensis NIES 3383
Colemanosphaera angeleri
Platydorina caudata
Volvox ferrisii
Volvox kirkiorum
Volvox barberi
Volvox globator
Gonium multicoccum
Gonium viridistellatum
Gonium quadratum
Gonium octonarium
Gonium pectorale
Astrephomene gubernaculifera
Astrephomene perforata
Chlamydomonas globosa
Chlamydomonas reinhardtii
Chlamydomonas schloesseri
Chlamydomonas debaryana SAG 70 81
Chlamydomonas debaryana SAG 1155a
Tetrabaena socialis NIES 571
Tetrabaena socialis NIES 691
Basichlamys sacculifera
Vitreochlamys ordinata
Vitreochlamys aulata NIES 878
Vitreochlamys aulata SAG 80 81
Vitreochlamys nekrassovii
Chlamydomonas moewusii

Fig. S19

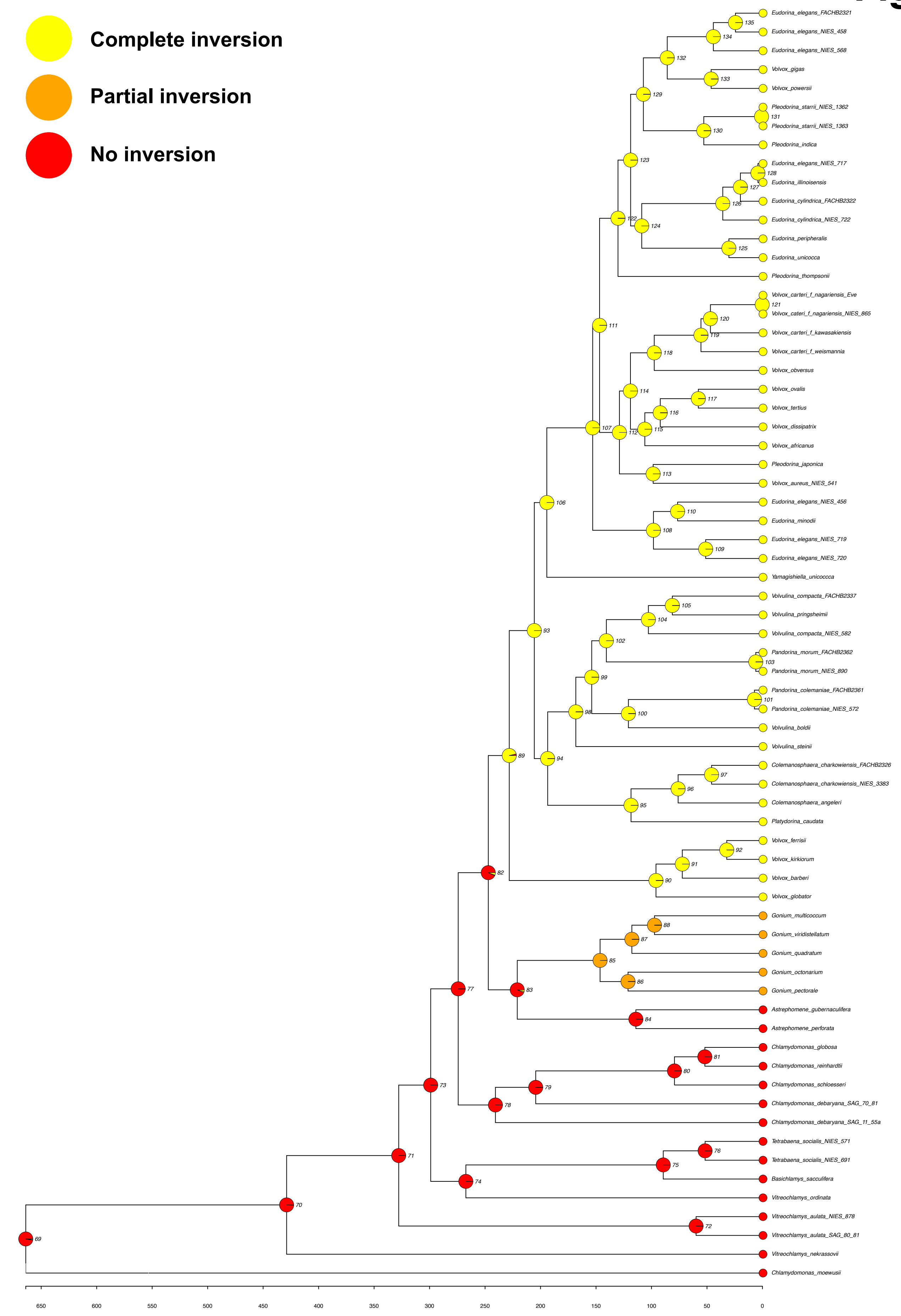

Complete inversion
Partial inversion
No inversion
Eudorina_elegans_FACHB2321
Eudorina_elegans_NIES_458
Eudorina_elegans_NIES_568
Volvox_gigas
Volvox_powersii
Pleodorina_starrii_NIES_1362
Pleodorina_starrii_NIES_1363
Pleodorina_indica
Eudorina_elegans_NIES_717
Eudorina_illinoisensis
Eudorina_cylindrica_FACHB2322
Eudorina_cylindrica_NIES_722
Eudorina_peripheralis
Eudorina_unicocca
Pleodorina_thompsonii
Volvox_carteri_f_nagariensis_Eve
Volvox_cateri_f_nagariensis_NIES_865
Volvox_carteri_f_kawasakiensis
Volvox_carteri_f_weismannia
Volvox_obversus
Volvox_ovalis
Volvox_tertius
Volvox_dissipatrix
Volvox_africanus
Pleodorina_japonica
Volvox_aureus_NIES_541
Eudorina_elegans_NIES_456
Eudorina_minodii
Eudorina_elegans_NIES_719
Eudorina_elegans_NIES_720
Yamagishiella_unicoccca
Volvulina_compacta_FACHB2337
Volvulina_pringsheimii
Volvulina_compacta_NIES_582
Pandorina_morum_FACHB2362
Pandorina_morum_NIES_890
Pandorina_colemaniae_FACHB2361
Pandorina_colemaniae_NIES_572
Volvulina_boldii
Volvulina_steinii
Colemanosphaera_charkowiensis_FACHB2326
Colemanosphaera_charkowiensis_NIES_3383
Colemanosphaera_angeleri
Platydorina_caudata
Volvox_ferrisii
Volvox_kirkiorum
Volvox_barberi
Volvox_globator
Gonium_multicoccum
Gonium_viridistellatum
Gonium_quadratum
Gonium_octonarium
Gonium_pectorale
Astrephomene_gubernaculifera
Astrephomene_perforata
Chlamydomonas_globosa
Chlamydomonas_reinhardtii
Chlamydomonas_schloesseri
Chlamydomonas_debaryana_SAG_70_81
Chlamydomonas_debaryana_SAG_11_55a
Tetrabaena_socialis_NIES_571
Tetrabaena_socialis_NIES_691
Basichlamys_sacculifera
Vitreochlamys_ordinata
Vitreochlamys_aulata_NIES_878
Vitreochlamys_aulata_SAG_80_81
Vitreochlamys_nekrassovii
Chlamydomonas_moewusii
650 600 550 500 450 400 350 300 250 200 150 100 50 0

Fig. S20

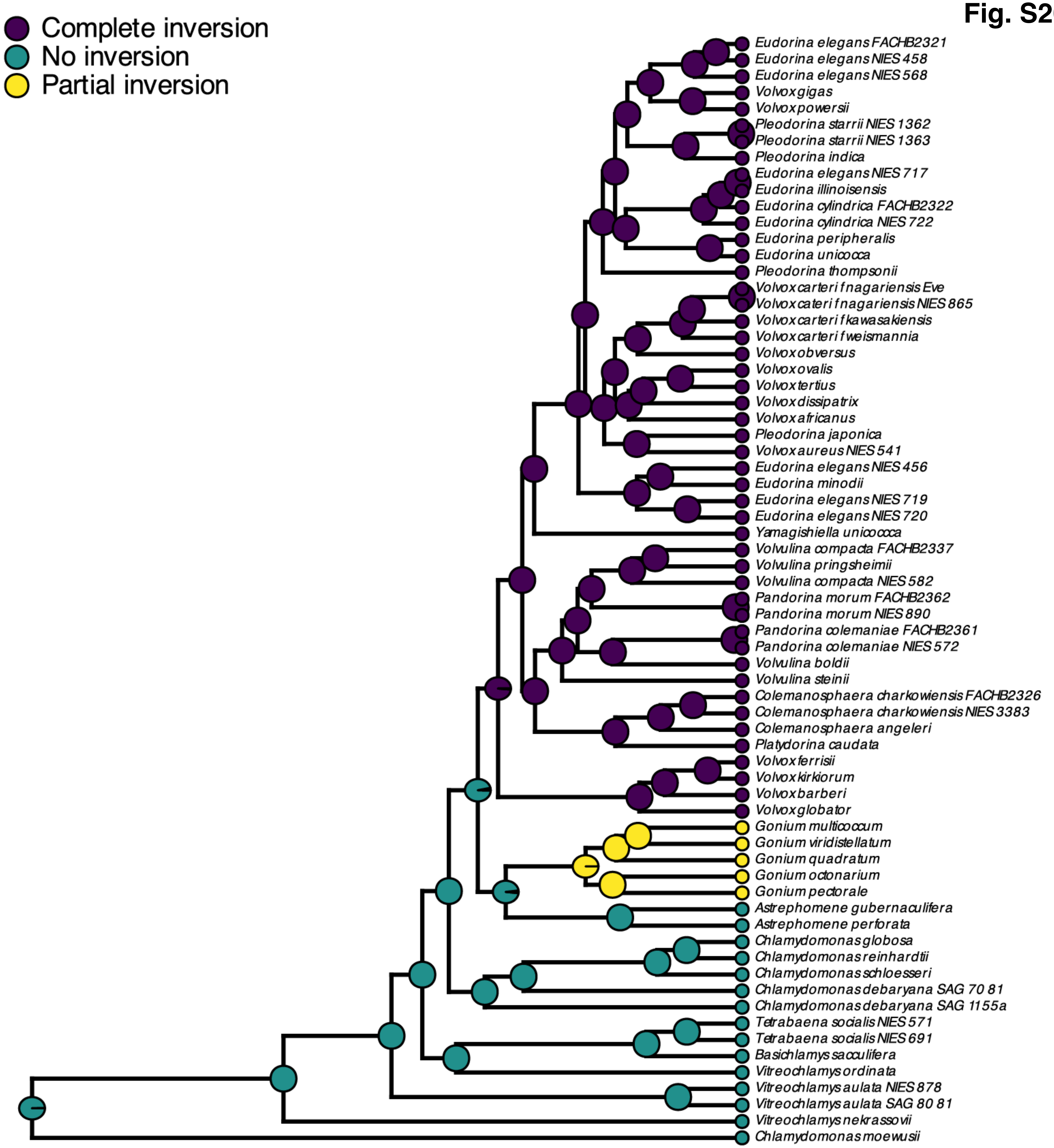
Complete inversion
No inversion
Partial inversion
Eudorina elegans FACHB2321
Eudorina elegans NIES 458
Eudorina elegans NIES 568
Volvox gigas
Volvox powersii
Pleodorina starrii NIES 1362
Pleodorina starrii NIES 1363
Pleodorina indica
Eudorina elegans NIES 717
Eudorina illinoisensis
Eudorina cylindrica FACHB2322
Eudorina cylindrica NIES 722
Eudorina peripheralis
Eudorina unicocca
Pleodorina thompsonii
Volvox carteri f nagariensis Eve
Volvox cateri f nagariensis NIES 865
Volvox carteri f kawasakiensis
Volvox carteri f weismannia
Volvox obversus
Volvox ovalis
Volvox tertius
Volvox dissipatrix
Volvox africanus
Pleodorina japonica
Volvox aureus NIES 541
Eudorina elegans NIES 456
Eudorina minodii
Eudorina elegans NIES 719
Eudorina elegans NIES 720
Yamagishiella unicocca
Volvulina compacta FACHB2337
Volvulina pringsheimii
Volvulina compacta NIES 582
Pandorina morum FACHB2362
Pandorina morum NIES 890
Pandorina colemaniae FACHB2361
Pandorina colemaniae NIES 572
Volvulina boldii
Volvulina steinii
Colemanosphaera charkowiensis FACHB2326
Colemanosphaera charkowiensis NIES 3383
Colemanosphaera angeleri
Platydorina caudata
Volvox ferrisii
Volvox kirkiorum
Volvox barberi
Volvox globator
Gonium multicoccum
Gonium viridistellatum
Gonium quadratum
Gonium octonarium
Gonium pectorale
Astrephomene gubernaculifera
Astrephomene perforata
Chlamydomonas globosa
Chlamydomonas reinhardtii
Chlamydomonas schloesseri
Chlamydomonas debaryana SAG 70 81
Chlamydomonas debaryana SAG 1155a
Tetrabaena socialis NIES 571
Tetrabaena socialis NIES 691
Basichlamys sacculifera
Vitreochlamys ordinata
Vitreochlamys aulata NIES 878
Vitreochlamys aulata SAG 80 81
Vitreochlamys nekrassovii
Chlamydomonas moewusii

Fig. S21

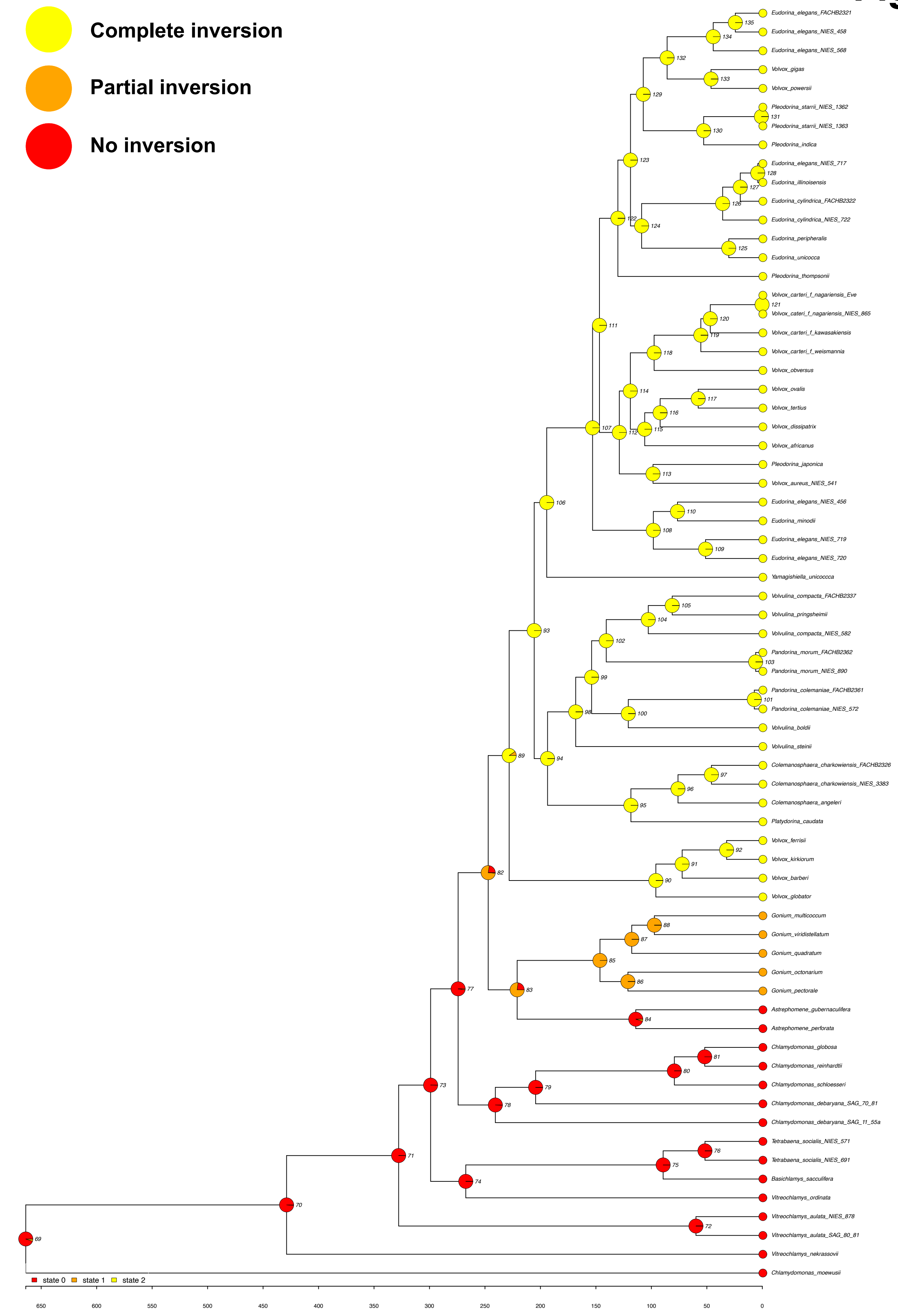

Complete inversion
Partial inversion
No inversion
Eudorina_elegans_FACHB2321
Eudorina_elegans_NIES_458
Eudorina_elegans_NIES_568
Volvox_gigas
Volvox_powersii
Pleodorina_starrii_NIES_1362
Pleodorina_starrii_NIES_1363
Pleodorina_indica
Eudorina_elegans_NIES_717
Eudorina_illinoisensis
Eudorina_cylindrica_FACHB2322
Eudorina_cylindrica_NIES_722
Eudorina_peripheralis
Eudorina_unicocca
Pleodorina_thompsonii
Volvox_carteri_f_nagariensis_Eve
Volvox_cateri_f_nagariensis_NIES_865
Volvox_carteri_f_kawasakiensis
Volvox_carteri_f_weismannia
Volvox_obversus
Volvox_ovalis
Volvox_tertius
Volvox_dissipatrix
Volvox_africanus
Pleodorina_japonica
Volvox_aureus_NIES_541
Eudorina_elegans_NIES_456
Eudorina_minodii
Eudorina_elegans_NIES_719
Eudorina_elegans_NIES_720
Yamagishiella_unicoccca
Volvulina_compacta_FACHB2337
Volvulina_pringsheimii
Volvulina_compacta_NIES_582
Pandorina_morum_FACHB2362
Pandorina_morum_NIES_890
Pandorina_colemaniae_FACHB2361
Pandorina_colemaniae_NIES_572
Volvulina_boldii
Volvulina_steinii
Colemanosphaera_charkowiensis_FACHB2326
Colemanosphaera_charkowiensis_NIES_3383
Colemanosphaera_angeleri
Platydorina_caudata
Volvox_ferrisii
Volvox_kirkiorum
Volvox_barberi
Volvox_globator
Gonium_multicoccum
Gonium_viridistellatum
Gonium_quadratum
Gonium_octonarium
Gonium_pectorale
Astrephomene_gubernaculifera
Astrephomene_perforata
Chlamydomonas_globosa
Chlamydomonas_reinhardtii
Chlamydomonas_schloesseri
Chlamydomonas_debaryana_SAG_70_81
Chlamydomonas_debaryana_SAG_11_55a
Tetrabaena_socialis_NIES_571
Tetrabaena_socialis_NIES_691
Basichlamys_sacculifera
Vitreochlamys_ordinata
Vitreochlamys_aulata_NIES_878
Vitreochlamys_aulata_SAG_80_81
Vitreochlamys_nekrassovii
Chlamydomonas_moewusii
state 0
state 1
state 2
650
600
550
500
450
400
350
300
250
200
150
100
50
0

Fig. S22

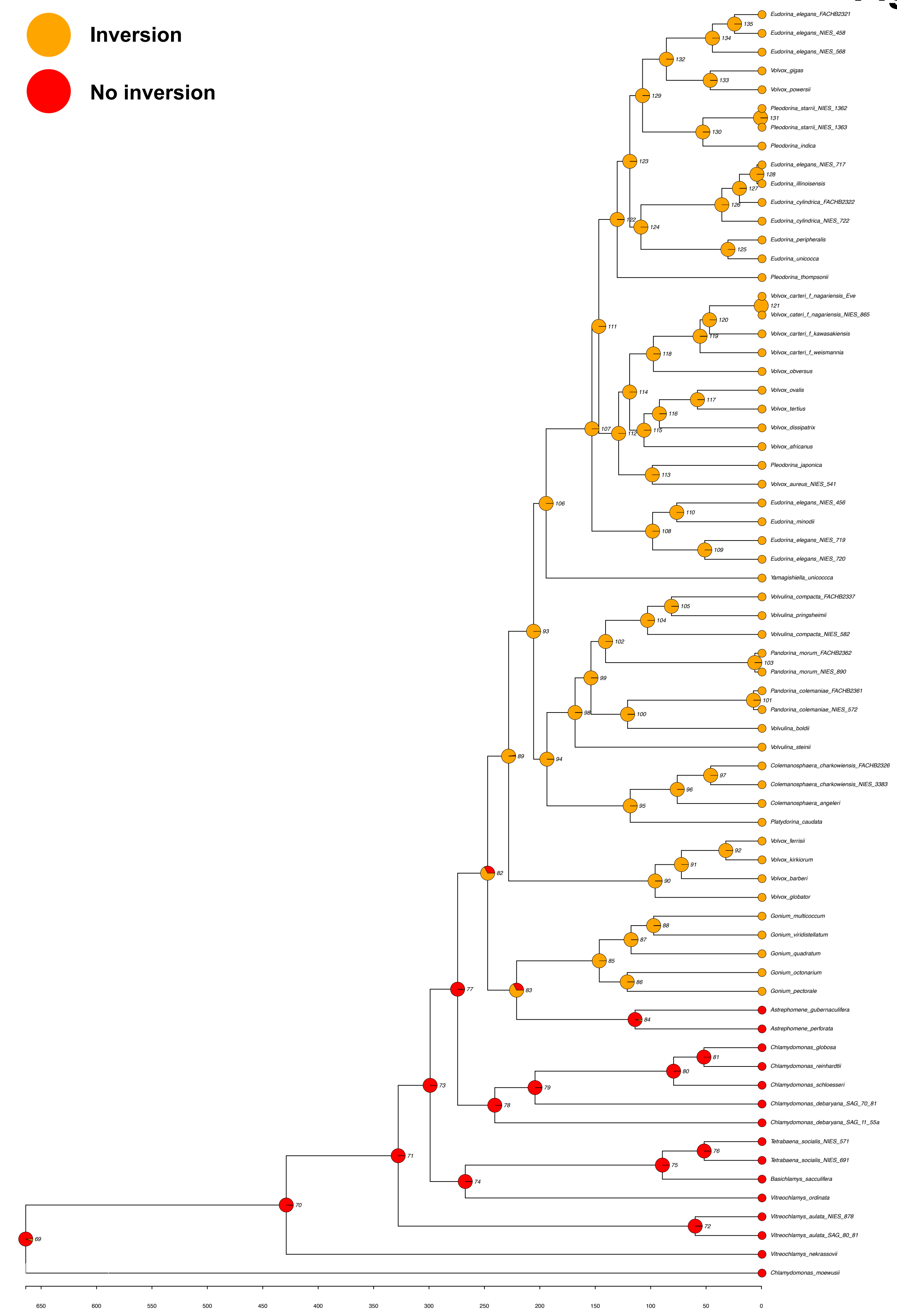
Inversion
No inversion
Eudorina_elegans_FACHB2321
Eudorina_elegans_NIES_458
Eudorina_elegans_NIES_568
Volvox_gigas
Volvox_powersii
Pleodorina_starrii_NIES_1362
Pleodorina_starrii_NIES_1363
Pleodorina_indica
Eudorina_elegans_NIES_717
Eudorina_illinoisensis
Eudorina_cylindrica_FACHB2322
Eudorina_cylindrica_NIES_722
Eudorina_peripheralis
Eudorina_unicocca
Pleodorina_thompsonii
Volvox_carteri_f_nagariensis_Eve
Volvox_cateri_f_nagariensis_NIES_865
Volvox_carteri_f_kawasakiensis
Volvox_carteri_f_weismannia
Volvox_obversus
Volvox_ovalis
Volvox_tertius
Volvox_dissipatrix
Volvox_africanus
Pleodorina_japonica
Volvox_aureus_NIES_541
Eudorina_elegans_NIES_456
Eudorina_minodii
Eudorina_elegans_NIES_719
Eudorina_elegans_NIES_720
Yamagishiella_unicoccca
Volvulina_compacta_FACHB2337
Volvulina_pringsheimii
Volvulina_compacta_NIES_582
Pandorina_morum_FACHB2362
Pandorina_morum_NIES_890
Pandorina_colemaniae_FACHB2361
Pandorina_colemaniae_NIES_572
Volvulina_boldii
Volvulina_steinii
Colemanosphaera_charkowiensis_FACHB2326
Colemanosphaera_charkowiensis_NIES_3383
Colemanosphaera_angeleri
Platydorina_caudata
Volvox_ferrisii
Volvox_kirkiorum
Volvox_barberi
Volvox_globator
Gonium_multicoccum
Gonium_viridistellatum
Gonium_quadratum
Gonium_octonarium
Gonium_pectorale
Astrephomene_gubernaculifera
Astrephomene_perforata
Chlamydomonas_globosa
Chlamydomonas_reinhardtii
Chlamydomonas_schloesseri
Chlamydomonas_debaryana_SAG_70_81
Chlamydomonas_debaryana_SAG_11_55a
Tetrabaena_socialis_NIES_571
Tetrabaena_socialis_NIES_691
Basichlamys_sacculifera
Vitreochlamys_ordinata
Vitreochlamys_aulata_NIES_878
Vitreochlamys_aulata_SAG_80_81
Vitreochlamys_nekrassovii
Chlamydomonas_moewusii
135
134
132
133
129
131
130
123
128
127
126
122
124
125
121
120
111
119
118
114
117
116
107
112
115
113
106
110
108
109
105
104
102
103
93
99
101
98
100
89
94
97
96
95
92
91
82
90
88
87
85
86
83
84
77
81
80
73
79
78
76
75
71
74
70
72
69
650
600
550
500
450
400
350
300
250
200
150
100
50
0

Fig. S23

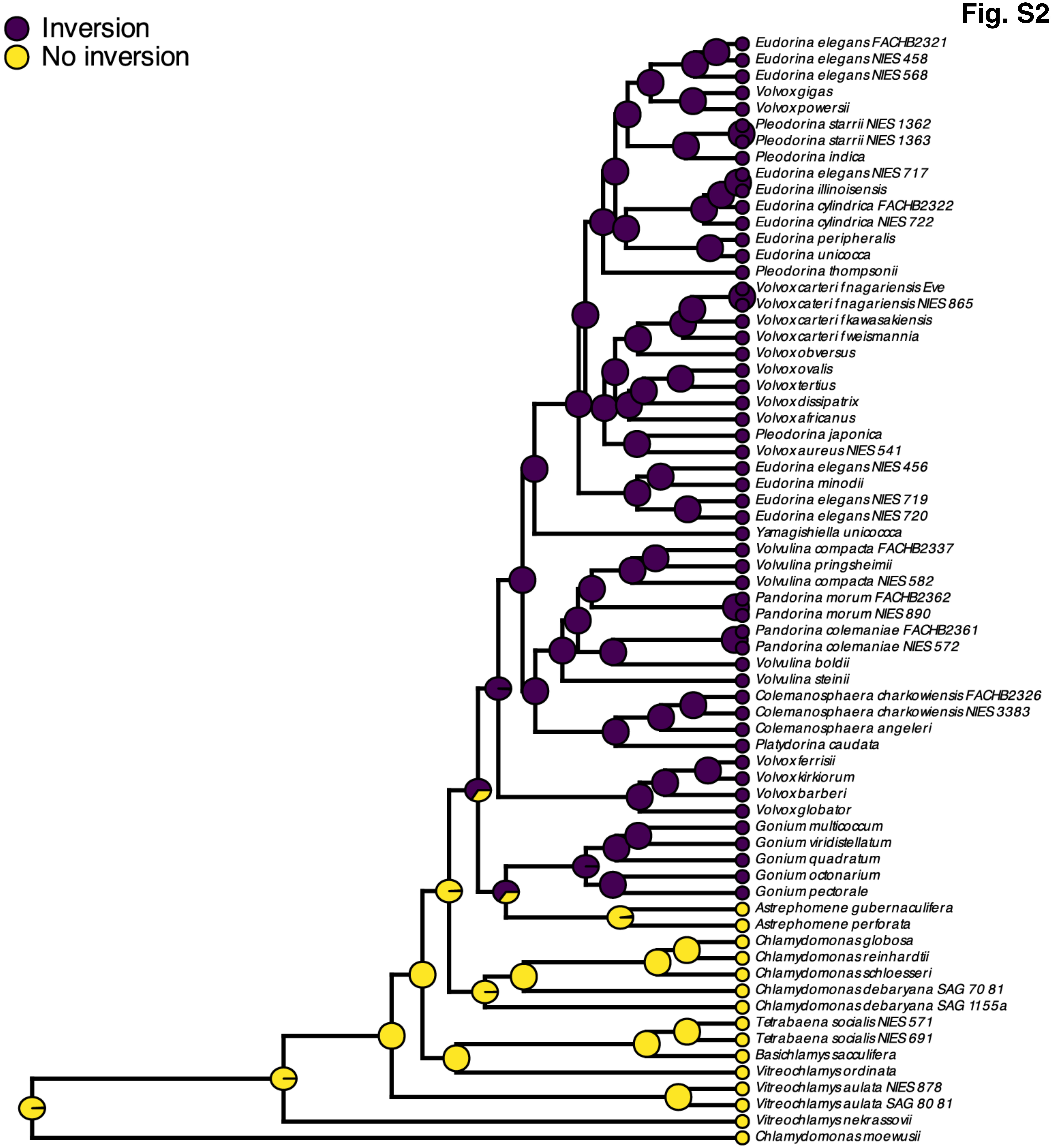
Inversion
No inversion
Eudorina elegans FACHB2321
Eudorina elegans NIES 458
Eudorina elegans NIES 568
Volvox gigas
Volvox powersii
Pleodorina starrii NIES 1362
Pleodorina starrii NIES 1363
Pleodorina indica
Eudorina elegans NIES 717
Eudorina illinoisensis
Eudorina cylindrica FACHB2322
Eudorina cylindrica NIES 722
Eudorina peripheralis
Eudorina unicocca
Pleodorina thompsonii
Volvox carteri f nagariensis Eve
Volvox cateri f nagariensis NIES 865
Volvox carteri f kawasakiensis
Volvox carteri f weismannia
Volvox obversus
Volvox ovalis
Volvox tertius
Volvox dissipatrix
Volvox africanus
Pleodorina japonica
Volvox aureus NIES 541
Eudorina elegans NIES 456
Eudorina minodii
Eudorina elegans NIES 719
Eudorina elegans NIES 720
Yamagishiella unicocca
Volvulina compacta FACHB2337
Volvulina pringsheimii
Volvulina compacta NIES 582
Pandorina morum FACHB2362
Pandorina morum NIES 890
Pandorina colemaniae FACHB2361
Pandorina colemaniae NIES 572
Volvulina boldii
Volvulina steinii
Colemanosphaera charkowiensis FACHB2326
Colemanosphaera charkowiensis NIES 3383
Colemanosphaera angeleri
Platydorina caudata
Volvox ferrisii
Volvox kirkiorum
Volvox barberi
Volvox globator
Gonium multicoccum
Gonium viridistellatum
Gonium quadratum
Gonium octonarium
Gonium pectorale
Astrephomene gubernaculifera
Astrephomene perforata
Chlamydomonas globosa
Chlamydomonas reinhardtii
Chlamydomonas schloesseri
Chlamydomonas debaryana SAG 70 81
Chlamydomonas debaryana SAG 1155a
Tetrabaena socialis NIES 571
Tetrabaena socialis NIES 691
Basichlamys sacculifera
Vitreochlamys ordinata
Vitreochlamys aulata NIES 878
Vitreochlamys aulata SAG 80 81
Vitreochlamys nekrassovii
Chlamydomonas moewusii

Fig. S24

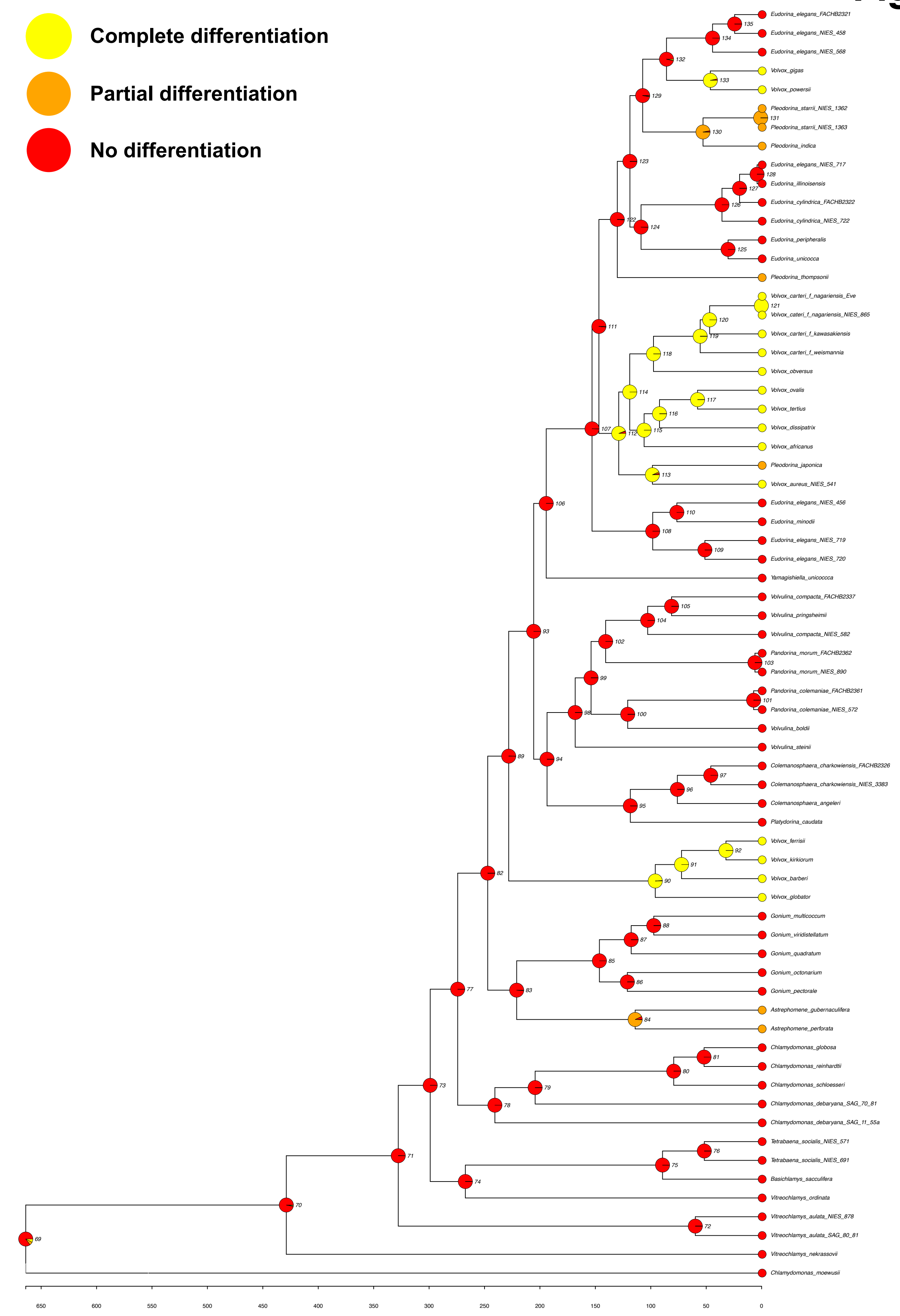

Complete differentiation
Partial differentiation
No differentiation
Eudorina_elegans_FACHB2321
Eudorina_elegans_NIES_458
Eudorina_elegans_NIES_568
Volvox_gigas
Volvox_powersii
Pleodorina_starrii_NIES_1362
Pleodorina_starrii_NIES_1363
Pleodorina_indica
Eudorina_elegans_NIES_717
Eudorina_illinoisensis
Eudorina_cylindrica_FACHB2322
Eudorina_cylindrica_NIES_722
Eudorina_peripheralis
Eudorina_unicocca
Pleodorina_thompsonii
Volvox_carteri_f_nagariensis_Eve
Volvox_cateri_f_nagariensis_NIES_865
Volvox_carteri_f_kawasakiensis
Volvox_carteri_f_weismannia
Volvox_obversus
Volvox_ovalis
Volvox_tertius
Volvox_dissipatrix
Volvox_africanus
Pleodorina_japonica
Volvox_aureus_NIES_541
Eudorina_elegans_NIES_456
Eudorina_minodii
Eudorina_elegans_NIES_719
Eudorina_elegans_NIES_720
Yamagishiella_unicoccca
Volvulina_compacta_FACHB2337
Volvulina_pringsheimii
Volvulina_compacta_NIES_582
Pandorina_morum_FACHB2362
Pandorina_morum_NIES_890
Pandorina_colemaniae_FACHB2361
Pandorina_colemaniae_NIES_572
Volvulina_boldii
Volvulina_steinii
Colemanosphaera_charkowiensis_FACHB2326
Colemanosphaera_charkowiensis_NIES_3383
Colemanosphaera_angeleri
Platydorina_caudata
Volvox_ferrisii
Volvox_kirkiorum
Volvox_barberi
Volvox_globator
Gonium_multicoccum
Gonium_viridistellatum
Gonium_quadratum
Gonium_octonarium
Gonium_pectorale
Astrephomene_gubernaculifera
Astrephomene_perforata
Chlamydomonas_globosa
Chlamydomonas_reinhardtii
Chlamydomonas_schloesseri
Chlamydomonas_debaryana_SAG_70_81
Chlamydomonas_debaryana_SAG_11_55a
Tetrabaena_socialis_NIES_571
Tetrabaena_socialis_NIES_691
Basichlamys_sacculifera
Vitreochlamys_ordinata
Vitreochlamys_aulata_NIES_878
Vitreochlamys_aulata_SAG_80_81
Vitreochlamys_nekrassovii
Chlamydomonas_moewusii
650
600
550
500
450
400
350
300
250
200
150
100
50
0

Fig. S25

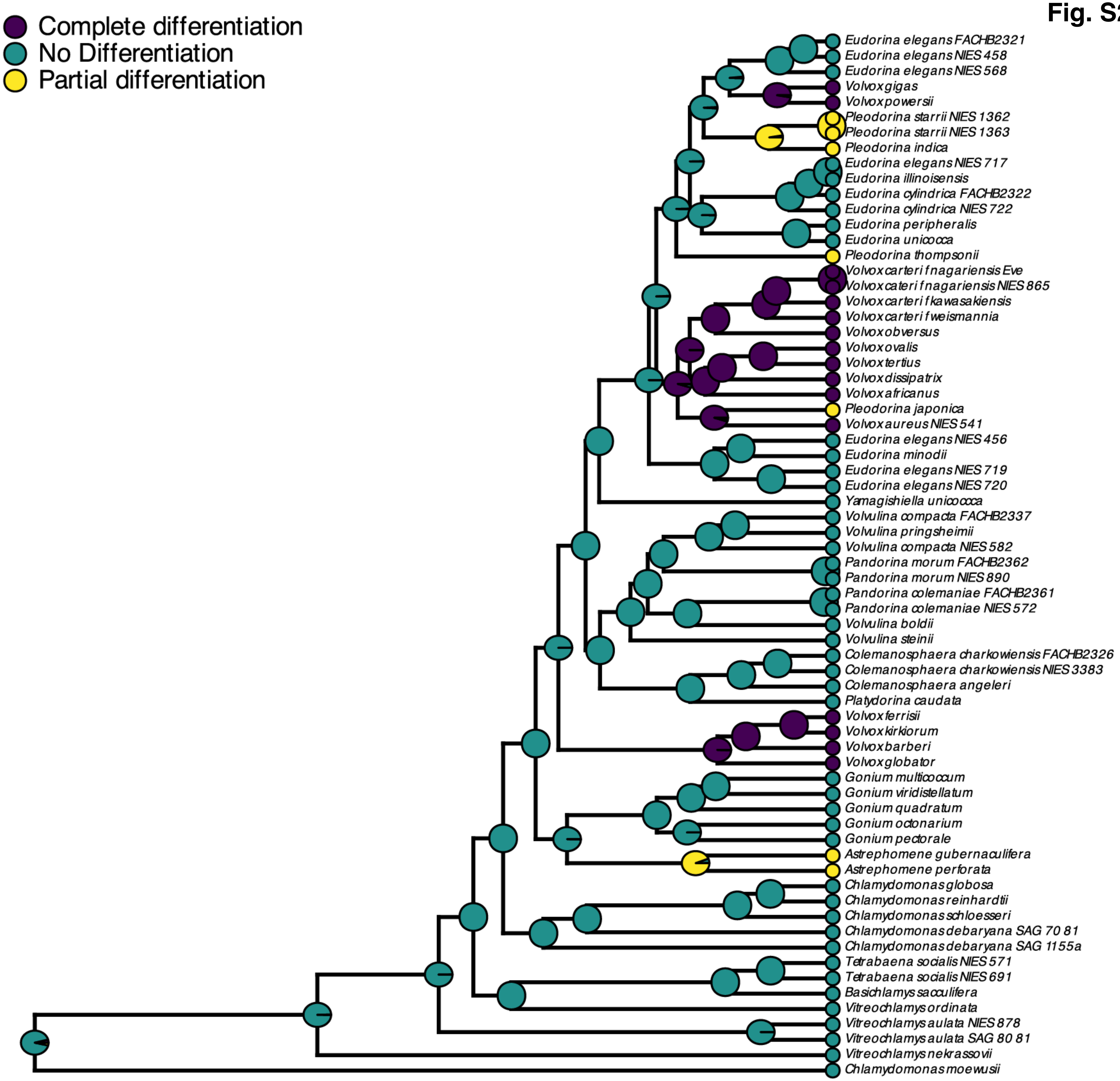
Complete differentiation
No Differentiation
Partial differentiation
Eudorina elegans FACHB2321
Eudorina elegans NIES 458
Eudorina elegans NIES 568
Volvox gigas
Volvox powersii
Pleodorina starrii NIES 1362
Pleodorina starrii NIES 1363
Pleodorina indica
Eudorina elegans NIES 717
Eudorina illinoisensis
Eudorina cylindrica FACHB2322
Eudorina cylindrica NIES 722
Eudorina peripheralis
Eudorina unicocca
Pleodorina thompsonii
Volvox carteri f nagariensis Eve
Volvox cateri f nagariensis NIES 865
Volvox carteri f kawasakiensis
Volvox carteri f weismannia
Volvox obversus
Volvox ovalis
Volvox tertius
Volvox dissipatrix
Volvox africanus
Pleodorina japonica
Volvox aureus NIES 541
Eudorina elegans NIES 456
Eudorina minodii
Eudorina elegans NIES 719
Eudorina elegans NIES 720
Yamagishiella unicocca
Volvulina compacta FACHB2337
Volvulina pringsheimii
Volvulina compacta NIES 582
Pandorina morum FACHB2362
Pandorina morum NIES 890
Pandorina colemaniae FACHB2361
Pandorina colemaniae NIES 572
Volvulina boldii
Volvulina steinii
Colemanosphaera charkowiensis FACHB2326
Colemanosphaera charkowiensis NIES 3383
Colemanosphaera angeleri
Platydorina caudata
Volvox ferrisii
Volvox kirkiorum
Volvox barberi
Volvox globator
Gonium multicoccum
Gonium viridistellatum
Gonium quadratum
Gonium octonarium
Gonium pectorale
Astrephomene gubernaculifera
Astrephomene perforata
Chlamydomonas globosa
Chlamydomonas reinhardtii
Chlamydomonas schloesseri
Chlamydomonas debaryana SAG 70 81
Chlamydomonas debaryana SAG 1155a
Tetrabaena socialis NIES 571
Tetrabaena socialis NIES 691
Basichlamys sacculifera
Vitreochlamys ordinata
Vitreochlamys aulata NIES 878
Vitreochlamys aulata SAG 80 81
Vitreochlamys nekrassovii
Chlamydomonas moewusii

Fig. S26

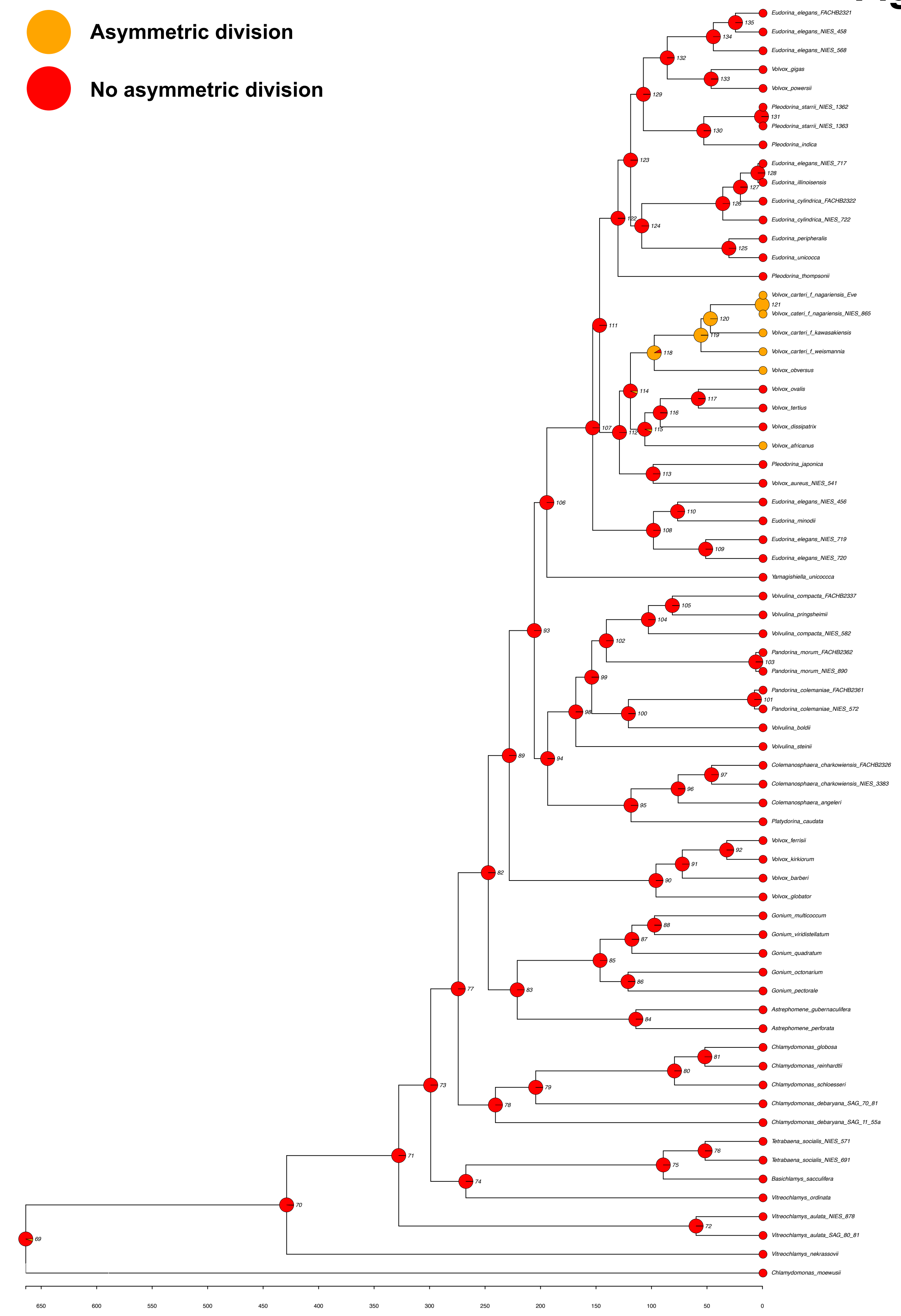

Asymmetric division
No asymmetric division
Eudorina_elegans_FACHB2321
Eudorina_elegans_NIES_458
Eudorina_elegans_NIES_568
Volvox_gigas
Volvox_powersii
Pleodorina_starrii_NIES_1362
Pleodorina_starrii_NIES_1363
Pleodorina_indica
Eudorina_elegans_NIES_717
Eudorina_illinoisensis
Eudorina_cylindrica_FACHB2322
Eudorina_cylindrica_NIES_722
Eudorina_peripheralis
Eudorina_unicocca
Pleodorina_thompsonii
Volvox_carteri_f_nagariensis_Eve
Volvox_cateri_f_nagariensis_NIES_865
Volvox_carteri_f_kawasakiensis
Volvox_carteri_f_weismannia
Volvox_obversus
Volvox_ovalis
Volvox_tertius
Volvox_dissipatrix
Volvox_africanus
Pleodorina_japonica
Volvox_aureus_NIES_541
Eudorina_elegans_NIES_456
Eudorina_minodii
Eudorina_elegans_NIES_719
Eudorina_elegans_NIES_720
Yamagishiella_unicoccca
Volvulina_compacta_FACHB2337
Volvulina_pringsheimii
Volvulina_compacta_NIES_582
Pandorina_morum_FACHB2362
Pandorina_morum_NIES_890
Pandorina_colemaniae_FACHB2361
Pandorina_colemaniae_NIES_572
Volvulina_boldii
Volvulina_steinii
Colemanosphaera_charkowiensis_FACHB2326
Colemanosphaera_charkowiensis_NIES_3383
Colemanosphaera_angeleri
Platydorina_caudata
Volvox_ferrisii
Volvox_kirkiorum
Volvox_barberi
Volvox_globator
Gonium_multicoccum
Gonium_viridistellatum
Gonium_quadratum
Gonium_octonarium
Gonium_pectorale
Astrephomene_gubernaculifera
Astrephomene_perforata
Chlamydomonas_globosa
Chlamydomonas_reinhardtii
Chlamydomonas_schloesseri
Chlamydomonas_debaryana_SAG_70_81
Chlamydomonas_debaryana_SAG_11_55a
Tetrabaena_socialis_NIES_571
Tetrabaena_socialis_NIES_691
Basichlamys_sacculifera
Vitreochlamys_ordinata
Vitreochlamys_aulata_NIES_878
Vitreochlamys_aulata_SAG_80_81
Vitreochlamys_nekrassovii
Chlamydomonas_moewusii
650 600 550 500 450 400 350 300 250 200 150 100 50 0

Fig. S27

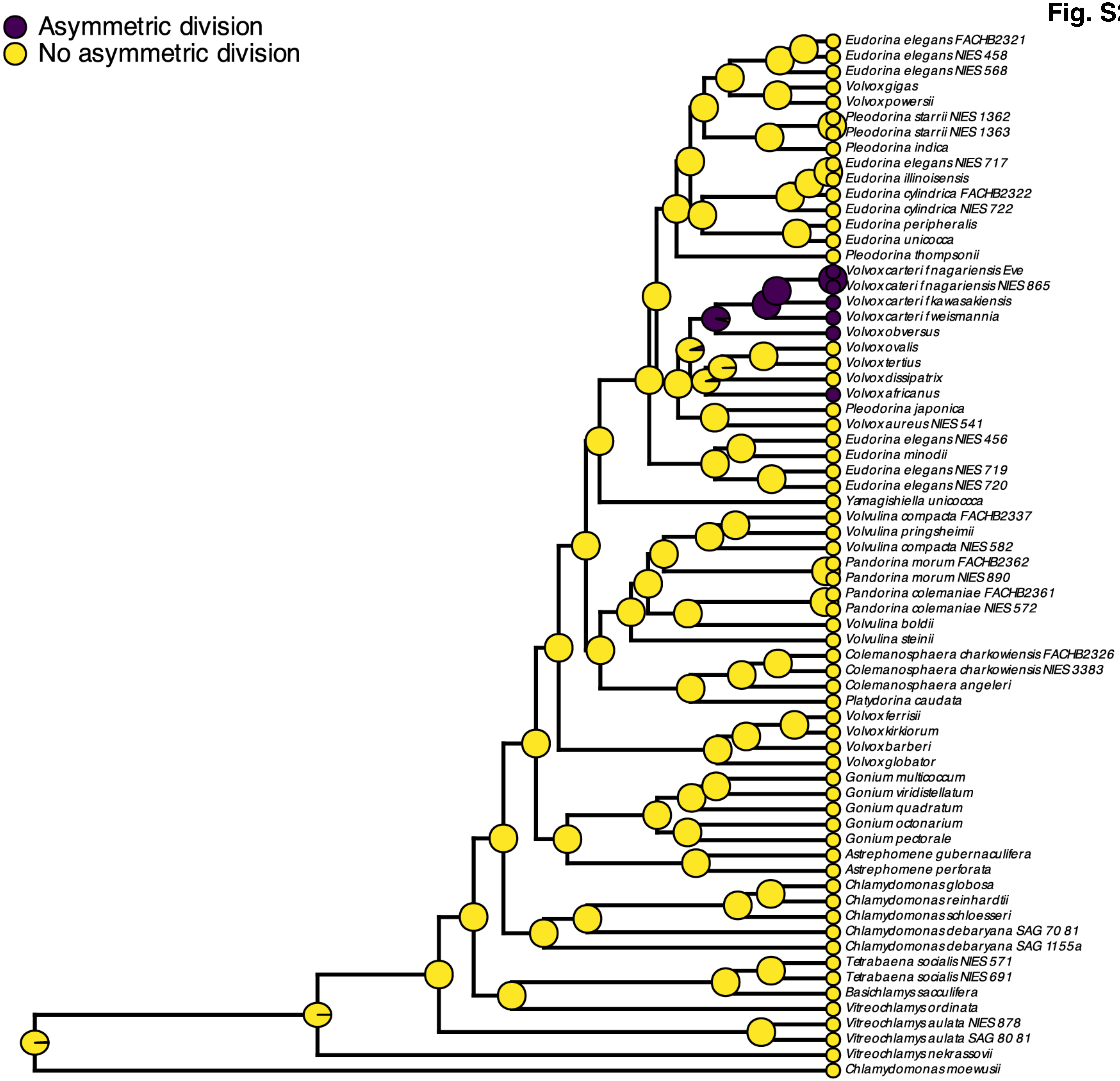

Asymmetric division
No asymmetric division
Eudorina elegans FACHB2321
Eudorina elegans NIES 458
Eudorina elegans NIES 568
Volvox gigas
Volvox powersii
Pleodorina starrii NIES 1362
Pleodorina starrii NIES 1363
Pleodorina indica
Eudorina elegans NIES 717
Eudorina illinoisensis
Eudorina cylindrica FACHB2322
Eudorina cylindrica NIES 722
Eudorina peripheralis
Eudorina unicocca
Pleodorina thompsonii
Volvox carteri f nagariensis Eve
Volvox cateri f nagariensis NIES 865
Volvox carteri f kawasakiensis
Volvox carteri f weismannia
Volvox obversus
Volvox ovalis
Volvox tertius
Volvox dissipatrix
Volvox africanus
Pleodorina japonica
Volvox aureus NIES 541
Eudorina elegans NIES 456
Eudorina minodii
Eudorina elegans NIES 719
Eudorina elegans NIES 720
Yamagishiella unicocca
Volvulina compacta FACHB2337
Volvulina pringsheimii
Volvulina compacta NIES 582
Pandorina morum FACHB2362
Pandorina morum NIES 890
Pandorina colemaniae FACHB2361
Pandorina colemaniae NIES 572
Volvulina boldii
Volvulina steinii
Colemanosphaera charkowiensis FACHB2326
Colemanosphaera charkowiensis NIES 3383
Colemanosphaera angeleri
Platydorina caudata
Volvox ferrisii
Volvox kirkiorum
Volvox barberi
Volvox globator
Gonium multicoccum
Gonium viridistellatum
Gonium quadratum
Gonium octonarium
Gonium pectorale
Astrephomene gubernaculifera
Astrephomene perforata
Chlamydomonas globosa
Chlamydomonas reinhardtii
Chlamydomonas schloesseri
Chlamydomonas debaryana SAG 70 81
Chlamydomonas debaryana SAG 1155a
Tetrabaena socialis NIES 571
Tetrabaena socialis NIES 691
Basichlamys sacculifera
Vitreochlamys ordinata
Vitreochlamys aulata NIES 878
Vitreochlamys aulata SAG 80 81
Vitreochlamys nekrassovii
Chlamydomonas moewusii

Fig. S28

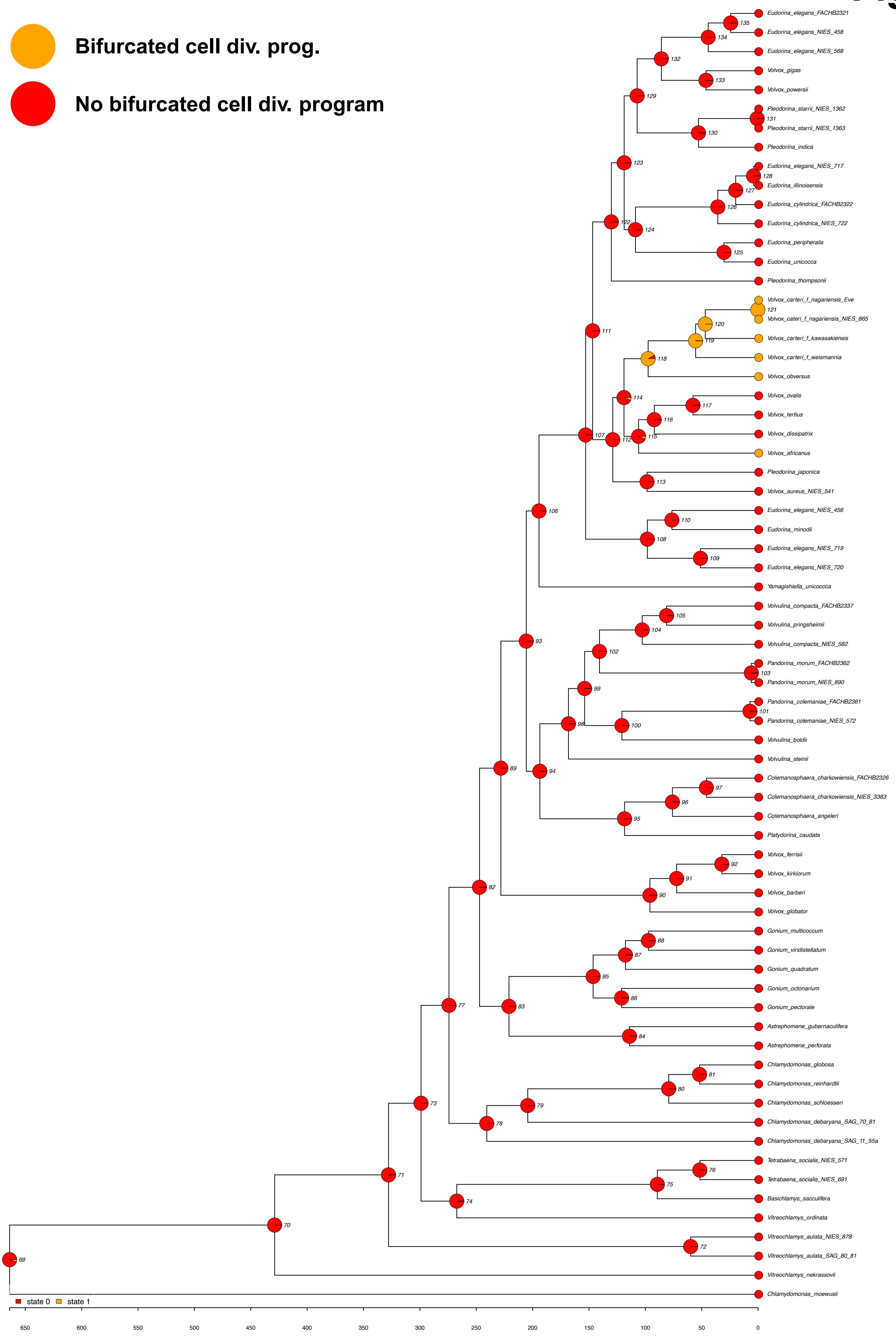

Bifurcated cell div. prog.
No bifurcated cell div. program
Eudorina_elegans_FACHB2321
Eudorina_elegans_NIES_458
Eudorina_elegans_NIES_568
Volvox_gigas
Volvox_powersii
Pleodorina_starrii_NIES_1362
Pleodorina_starrii_NIES_1363
Pleodorina_indica
Eudorina_elegans_NIES_717
Eudorina_illinoisensis
Eudorina_cylindrica_FACHB2322
Eudorina_cylindrica_NIES_722
Eudorina_peripheralis
Eudorina_unicocca
Pleodorina_thompsonii
Volvox_carteri_f_nagariensis_Eve
Volvox_cateri_f_nagariensis_NIES_865
Volvox_carteri_f_kawasakiensis
Volvox_carteri_f_weismannia
Volvox_obversus
Volvox_ovalis
Volvox_tertius
Volvox_dissipatrix
Volvox_africanus
Pleodorina_japonica
Volvox_aureus_NIES_541
Eudorina_elegans_NIES_456
Eudorina_minodii
Eudorina_elegans_NIES_719
Eudorina_elegans_NIES_720
Yamagishiella_unicoccca
Volvulina_compacta_FACHB2337
Volvulina_pringsheimii
Volvulina_compacta_NIES_582
Pandorina_morum_FACHB2362
Pandorina_morum_NIES_890
Pandorina_colemaniae_FACHB2361
Pandorina_colemaniae_NIES_572
Volvulina_boldii
Volvulina_steinii
Colemanosphaera_charkowiensis_FACHB2326
Colemanosphaera_charkowiensis_NIES_3383
Colemanosphaera_angeleri
Platydorina_caudata
Volvox_ferrisii
Volvox_kirkiorum
Volvox_barberi
Volvox_globator
Gonium_multicoccum
Gonium_viridistellatum
Gonium_quadratum
Gonium_octonarium
Gonium_pectorale
Astrephomene_gubernaculifera
Astrephomene_perforata
Chlamydomonas_globosa
Chlamydomonas_reinhardtii
Chlamydomonas_schloesseri
Chlamydomonas_debaryana_SAG_70_81
Chlamydomonas_debaryana_SAG_11_55a
Tetrabaena_socialis_NIES_571
Tetrabaena_socialis_NIES_691
Basichlamys_sacculifera
Vitreochlamys_ordinata
Vitreochlamys_aulata_NIES_878
Vitreochlamys_aulata_SAG_80_81
Vitreochlamys_nekrassovii
Chlamydomonas_moewusii
state 0
state 1
650
600
550
500
450
400
350
300
250
200
150
100
50
0

Fig. S29

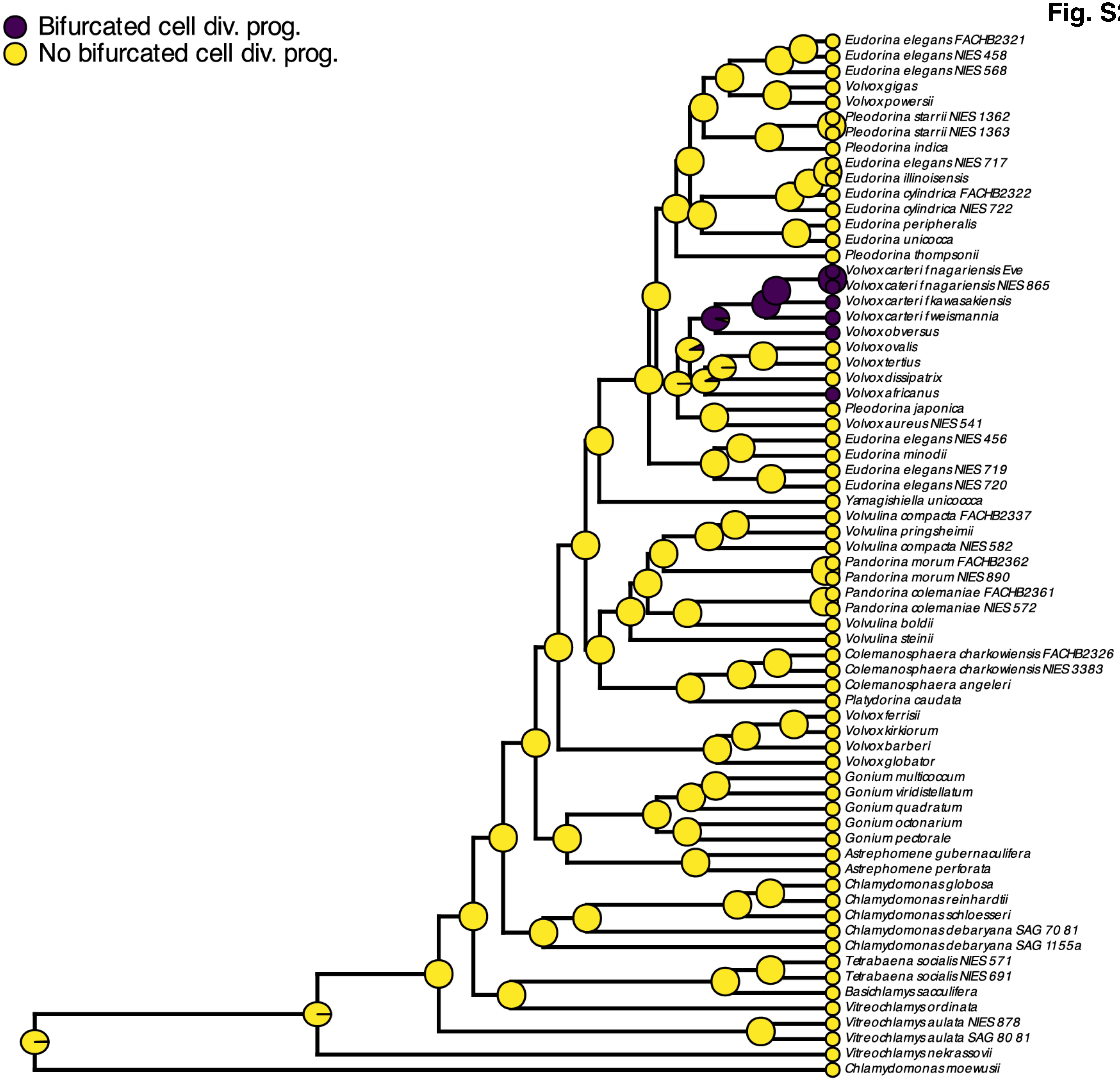

Bifurcated cell div. prog.
No bifurcated cell div. prog.
Eudorina elegans FACHB2321
Eudorina elegans NIES 458
Eudorina elegans NIES 568
Volvox gigas
Volvox powersii
Pleodorina starrii NIES 1362
Pleodorina starrii NIES 1363
Pleodorina indica
Eudorina elegans NIES 717
Eudorina illinoisensis
Eudorina cylindrica FACHB2322
Eudorina cylindrica NIES 722
Eudorina peripheralis
Eudorina unicocca
Pleodorina thompsonii
Volvox carteri f nagariensis Eve
Volvox cateri f nagariensis NIES 865
Volvox carteri f kawasakiensis
Volvox carteri f weismannia
Volvox obversus
Volvox ovalis
Volvox tertius
Volvox dissipatrix
Volvox africanus
Pleodorina japonica
Volvox aureus NIES 541
Eudorina elegans NIES 456
Eudorina minodii
Eudorina elegans NIES 719
Eudorina elegans NIES 720
Yamagishiella unicocca
Volvulina compacta FACHB2337
Volvulina pringsheimii
Volvulina compacta NIES 582
Pandorina morum FACHB2362
Pandorina morum NIES 890
Pandorina colemaniae FACHB2361
Pandorina colemaniae NIES 572
Volvulina boldii
Volvulina steinii
Colemanosphaera charkowiensis FACHB2326
Colemanosphaera charkowiensis NIES 3383
Colemanosphaera angeleri
Platydorina caudata
Volvox ferrisii
Volvox kirkiorum
Volvox barberi
Volvox globator
Gonium multicoccum
Gonium viridistellatum
Gonium quadratum
Gonium octonarium
Gonium pectorale
Astrephomene gubernaculifera
Astrephomene perforata
Chlamydomonas globosa
Chlamydomonas reinhardtii
Chlamydomonas schloesseri
Chlamydomonas debaryana SAG 70 81
Chlamydomonas debaryana SAG 1155a
Tetrabaena socialis NIES 571
Tetrabaena socialis NIES 691
Basichlamys sacculifera
Vitreochlamys ordinata
Vitreochlamys aulata NIES 878
Vitreochlamys aulata SAG 80 81
Vitreochlamys nekrassovii
Chlamydomonas moewusii

Fig. S30

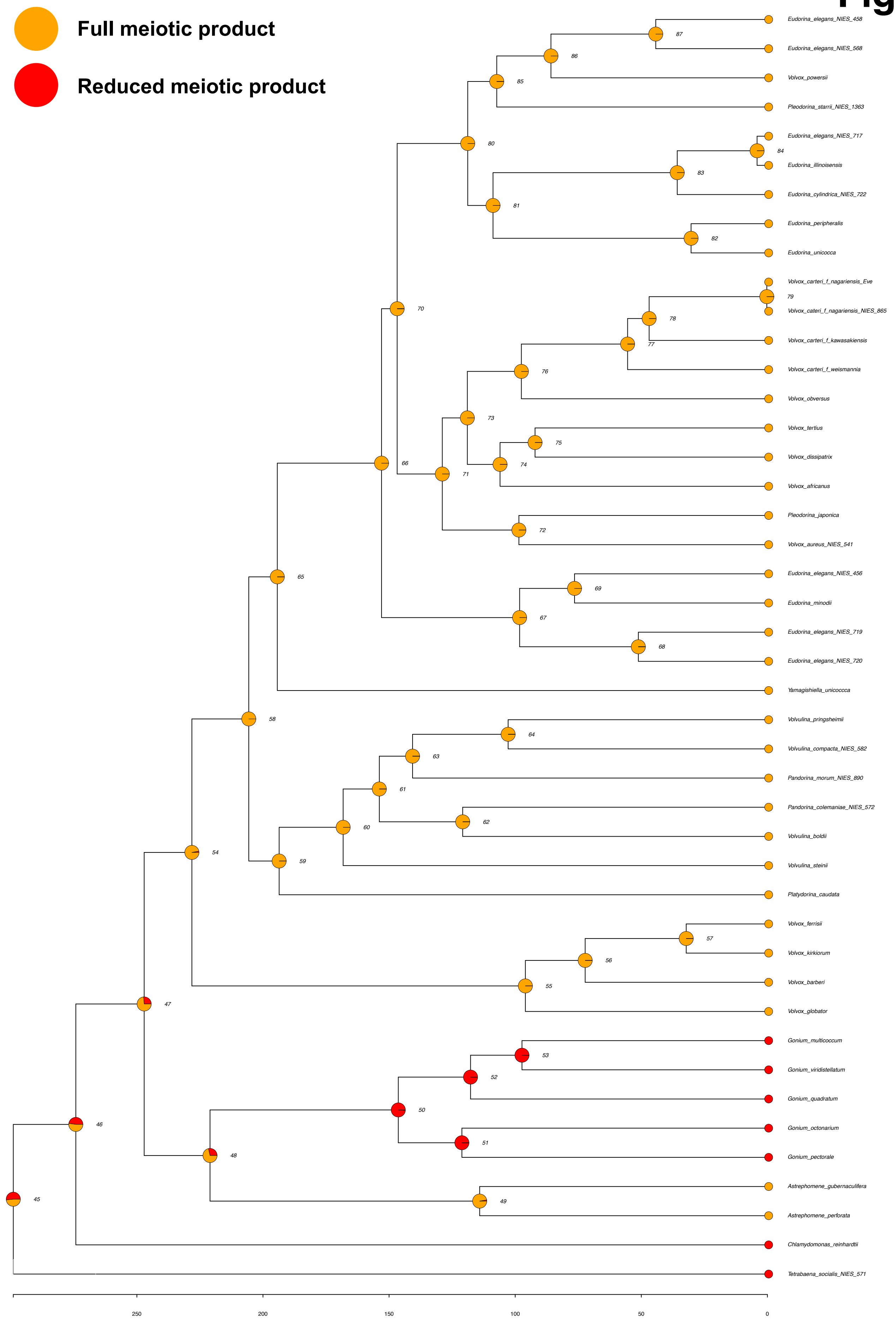

Full meiotic product
Reduced meiotic product
Eudorina_elegans_NIES_458
Eudorina_elegans_NIES_568
Volvox_powersii
Pleodorina_starrii_NIES_1363
Eudorina_elegans_NIES_717
Eudorina_illinoisensis
Eudorina_cylindrica_NIES_722
Eudorina_peripheralis
Eudorina_unicocca
Volvox_carteri_f_nagariensis_Eve
Volvox_cateri_f_nagariensis_NIES_865
Volvox_carteri_f_kawasakiensis
Volvox_carteri_f_weismannia
Volvox_obversus
Volvox_tertius
Volvox_dissipatrix
Volvox_africanus
Pleodorina_japonica
Volvox_aureus_NIES_541
Eudorina_elegans_NIES_456
Eudorina_minodii
Eudorina_elegans_NIES_719
Eudorina_elegans_NIES_720
Yamagishiella_unicoccca
Volvulina_pringsheimii
Volvulina_compacta_NIES_582
Pandorina_morum_NIES_890
Pandorina_colemaniae_NIES_572
Volvulina_boldii
Volvulina_steinii
Platydorina_caudata
Volvox_ferrisii
Volvox_kirkiorum
Volvox_barberi
Volvox_globator
Gonium_multicoccum
Gonium_viridistellatum
Gonium_quadratum
Gonium_octonarium
Gonium_pectorale
Astrephomene_gubernaculifera
Astrephomene_perforata
Chlamydomonas_reinhardtii
Tetrabaena_socialis_NIES_571
87
86
85
84
83
80
81
82
79
78
77
70
76
73
75
74
66
71
72
65
69
67
68
58
64
63
61
62
60
59
54
57
56
55
47
53
52
50
46
51
48
49
45
250
200
150
100
50
0

Fig. S31

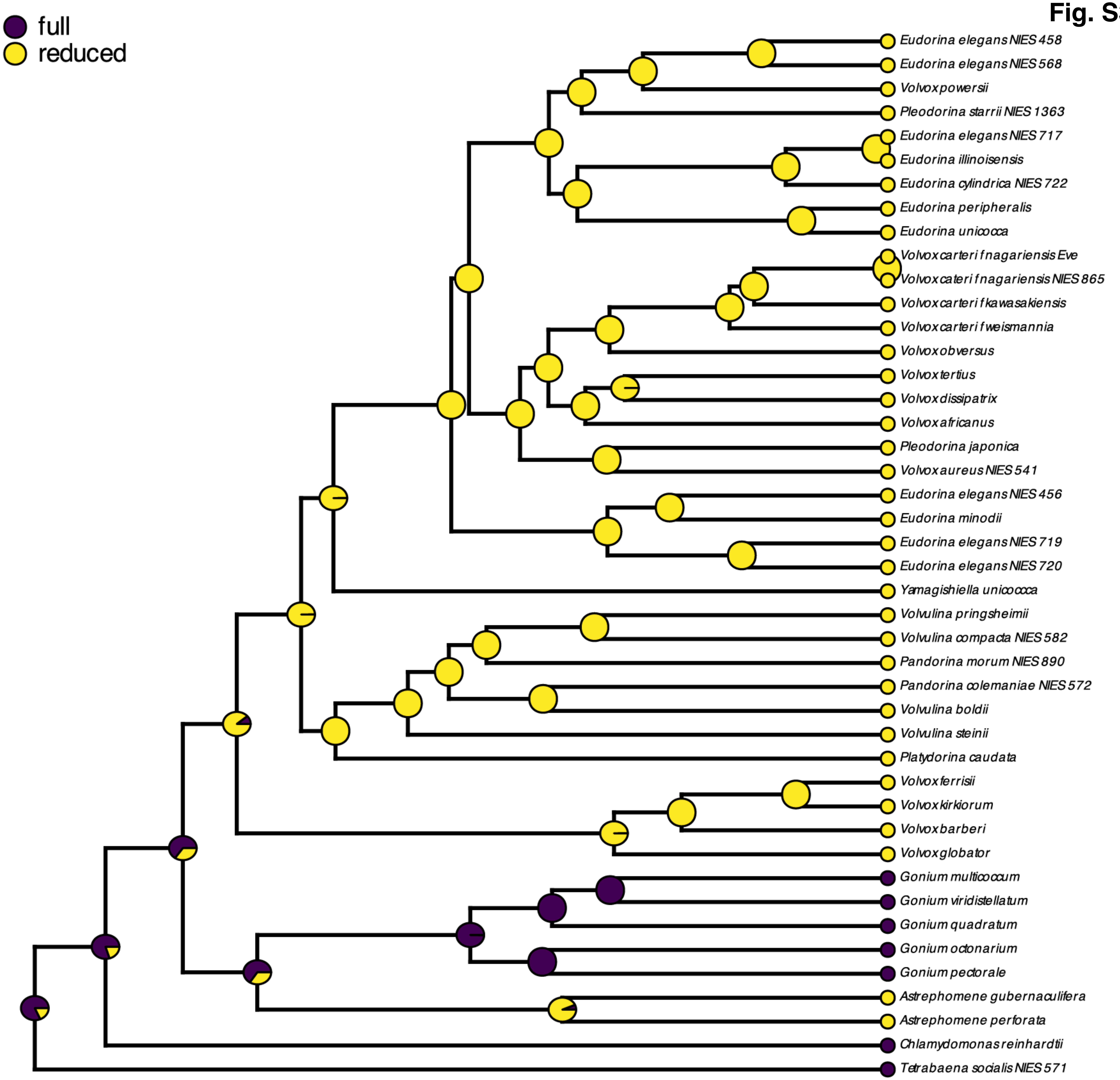

full
reduced
Eudorina elegans NIES 458
Eudorina elegans NIES 568
Volvox powersii
Pleodorina starrii NIES 1363
Eudorina elegans NIES 717
Eudorina illinoisensis
Eudorina cylindrica NIES 722
Eudorina peripheralis
Eudorina unicocca
Volvox carteri f nagariensis Eve
Volvox cateri f nagariensis NIES 865
Volvox carteri f kawasakiensis
Volvox carteri f weismannia
Volvox obversus
Volvox tertius
Volvox dissipatrix
Volvox africanus
Pleodorina japonica
Volvox aureus NIES 541
Eudorina elegans NIES 456
Eudorina minodii
Eudorina elegans NIES 719
Eudorina elegans NIES 720
Yamagishiella unicocca
Volvulina pringsheimii
Volvulina compacta NIES 582
Pandorina morum NIES 890
Pandorina colemaniae NIES 572
Volvulina boldii
Volvulina steinii
Platydorina caudata
Volvox ferrisii
Volvox kirkiorum
Volvox barberi
Volvox globator
Gonium multicoccum
Gonium viridistellatum
Gonium quadratum
Gonium octonarium
Gonium pectorale
Astrephomene gubernaculifera
Astrephomene perforata
Chlamydomonas reinhardtii
Tetrabaena socialis NIES 571

Fig. S32

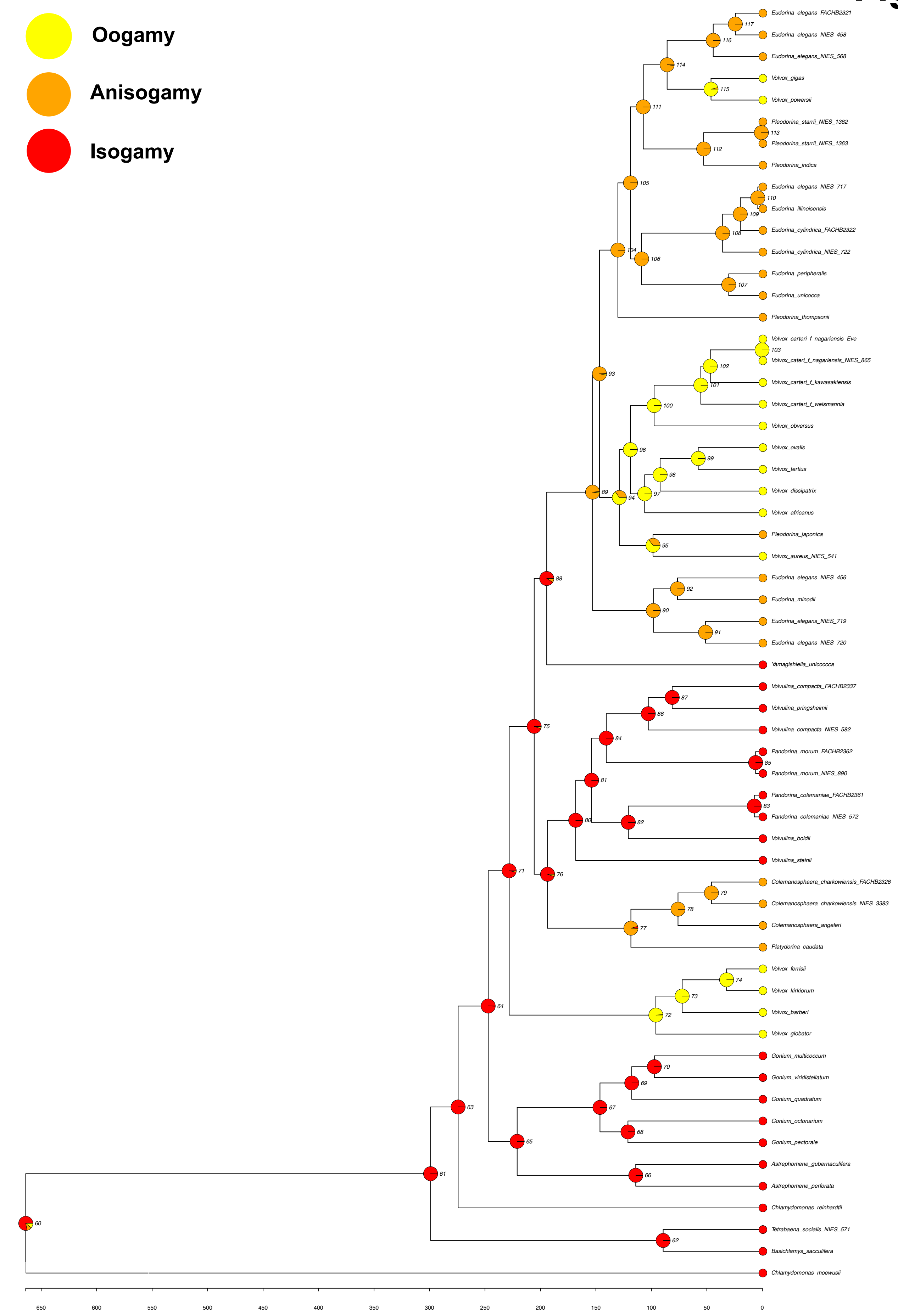

Oogamy
Anisogamy
Isogamy
Eudorina_elegans_FACHB2321
Eudorina_elegans_NIES_458
Eudorina_elegans_NIES_568
Volvox_gigas
Volvox_powersii
Pleodorina_starrii_NIES_1362
Pleodorina_starrii_NIES_1363
Pleodorina_indica
Eudorina_elegans_NIES_717
Eudorina_illinoisensis
Eudorina_cylindrica_FACHB2322
Eudorina_cylindrica_NIES_722
Eudorina_peripheralis
Eudorina_unicocca
Pleodorina_thompsonii
Volvox_carteri_f_nagariensis_Eve
Volvox_cateri_f_nagariensis_NIES_865
Volvox_carteri_f_kawasakiensis
Volvox_carteri_f_weismannia
Volvox_obversus
Volvox_ovalis
Volvox_tertius
Volvox_dissipatrix
Volvox_africanus
Pleodorina_japonica
Volvox_aureus_NIES_541
Eudorina_elegans_NIES_456
Eudorina_minodii
Eudorina_elegans_NIES_719
Eudorina_elegans_NIES_720
Yamagishiella_unicoccca
Volvulina_compacta_FACHB2337
Volvulina_pringsheimii
Volvulina_compacta_NIES_582
Pandorina_morum_FACHB2362
Pandorina_morum_NIES_890
Pandorina_colemaniae_FACHB2361
Pandorina_colemaniae_NIES_572
Volvulina_boldii
Volvulina_steinii
Colemanosphaera_charkowiensis_FACHB2326
Colemanosphaera_charkowiensis_NIES_3383
Colemanosphaera_angeleri
Platydorina_caudata
Volvox_ferrisii
Volvox_kirkiorum
Volvox_barberi
Volvox_globator
Gonium_multicoccum
Gonium_viridistellatum
Gonium_quadratum
Gonium_octonarium
Gonium_pectorale
Astrephomene_gubernaculifera
Astrephomene_perforata
Chlamydomonas_reinhardtii
Tetrabaena_socialis_NIES_571
Basichlamys_sacculifera
Chlamydomonas_moewusii
650
600
550
500
450
400
350
300
250
200
150
100
50
0

Fig. S33

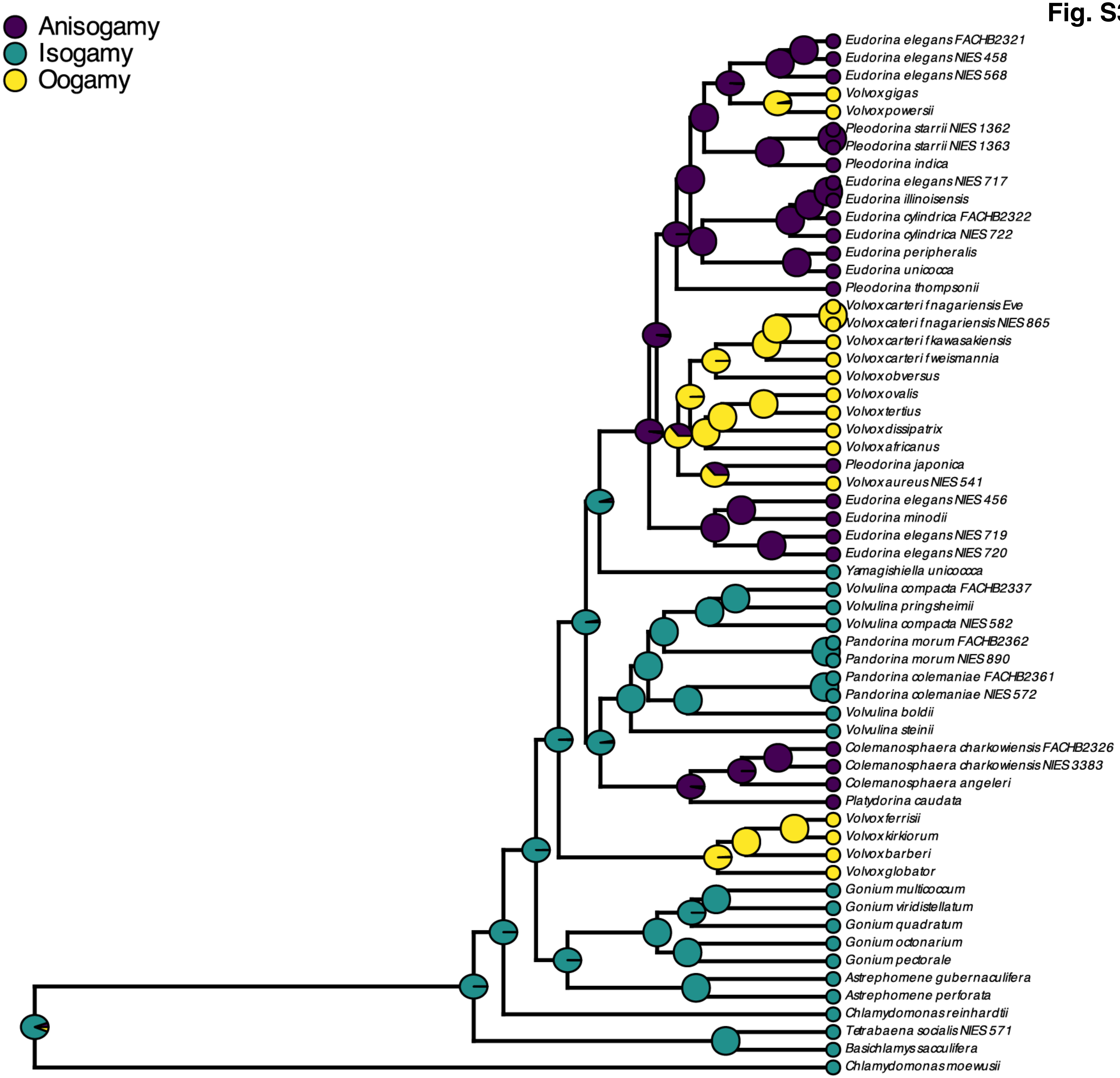
Anisogamy
Isogamy
Oogamy
Eudorina elegans FACHB2321
Eudorina elegans NIES 458
Eudorina elegans NIES 568
Volvox gigas
Volvox powersii
Pleodorina starrii NIES 1362
Pleodorina starrii NIES 1363
Pleodorina indica
Eudorina elegans NIES 717
Eudorina illinoisensis
Eudorina cylindrica FACHB2322
Eudorina cylindrica NIES 722
Eudorina peripheralis
Eudorina unicocca
Pleodorina thompsonii
Volvox carteri f nagariensis Eve
Volvox cateri f nagariensis NIES 865
Volvox carteri f kawasakiensis
Volvox carteri f weismannia
Volvox obversus
Volvox ovalis
Volvox tertius
Volvox dissipatrix
Volvox africanus
Pleodorina japonica
Volvox aureus NIES 541
Eudorina elegans NIES 456
Eudorina minodii
Eudorina elegans NIES 719
Eudorina elegans NIES 720
Yamagishiella unicocca
Volvulina compacta FACHB2337
Volvulina pringsheimii
Volvulina compacta NIES 582
Pandorina morum FACHB2362
Pandorina morum NIES 890
Pandorina colemaniae FACHB2361
Pandorina colemaniae NIES 572
Volvulina boldii
Volvulina steinii
Colemanosphaera charkowiensis FACHB2326
Colemanosphaera charkowiensis NIES 3383
Colemanosphaera angeleri
Platydorina caudata
Volvox ferrisii
Volvox kirkiorum
Volvox barberi
Volvox globator
Gonium multicoccum
Gonium viridistellatum
Gonium quadratum
Gonium octonarium
Gonium pectorale
Astrephomene gubernaculifera
Astrephomene perforata
Chlamydomonas reinhardtii
Tetrabaena socialis NIES 571
Basichlamys sacculifera
Chlamydomonas moewusii

Fig. S34

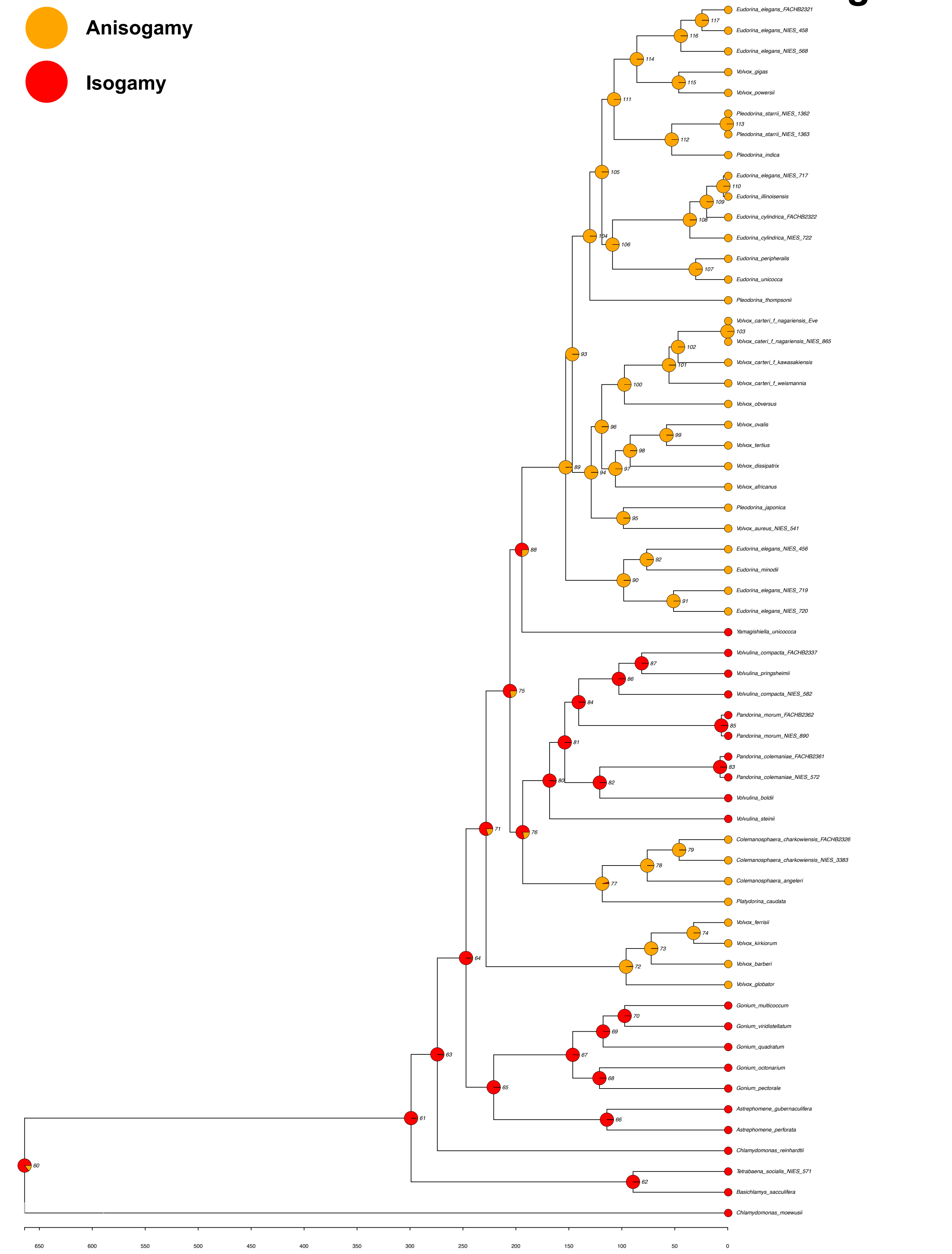
Anisogamy
Isogamy
Eudorina_elegans_FACHB2321
Eudorina_elegans_NIES_458
Eudorina_elegans_NIES_568
Volvox_gigas
Volvox_powersii
Pleodorina_starrii_NIES_1362
Pleodorina_starrii_NIES_1363
Pleodorina_indica
Eudorina_elegans_NIES_717
Eudorina_illinoisensis
Eudorina_cylindrica_FACHB2322
Eudorina_cylindrica_NIES_722
Eudorina_peripheralis
Eudorina_unicocca
Pleodorina_thompsonii
Volvox_carteri_f_nagariensis_Eve
Volvox_cateri_f_nagariensis_NIES_865
Volvox_carteri_f_kawasakiensis
Volvox_carteri_f_weismannia
Volvox_obversus
Volvox_ovalis
Volvox_tertius
Volvox_dissipatrix
Volvox_africanus
Pleodorina_japonica
Volvox_aureus_NIES_541
Eudorina_elegans_NIES_456
Eudorina_minodii
Eudorina_elegans_NIES_719
Eudorina_elegans_NIES_720
Yamagishiella_unicoccca
Volvulina_compacta_FACHB2337
Volvulina_pringsheimii
Volvulina_compacta_NIES_582
Pandorina_morum_FACHB2362
Pandorina_morum_NIES_890
Pandorina_colemaniae_FACHB2361
Pandorina_colemaniae_NIES_572
Volvulina_boldii
Volvulina_steinii
Colemanosphaera_charkowiensis_FACHB2326
Colemanosphaera_charkowiensis_NIES_3383
Colemanosphaera_angeleri
Platydorina_caudata
Volvox_ferrisii
Volvox_kirkiorum
Volvox_barberi
Volvox_globator
Gonium_multicoccum
Gonium_viridistellatum
Gonium_quadratum
Gonium_octonarium
Gonium_pectorale
Astrephomene_gubernaculifera
Astrephomene_perforata
Chlamydomonas_reinhardtii
Tetrabaena_socialis_NIES_571
Basichlamys_sacculifera
Chlamydomonas_moewusii
60 61 62 63 64 65 66 67 68 69 70 71 72 73 74 75 76 77 78 79 80 81 82 83 84 85 86 87 88 89 90 91 92 93 94 95 96 97 98 99 100 101 102 103 104 105 106 107 108 109 110 111 112 113 114 115 116 117
650 600 550 500 450 400 350 300 250 200 150 100 50 0

Fig. S35

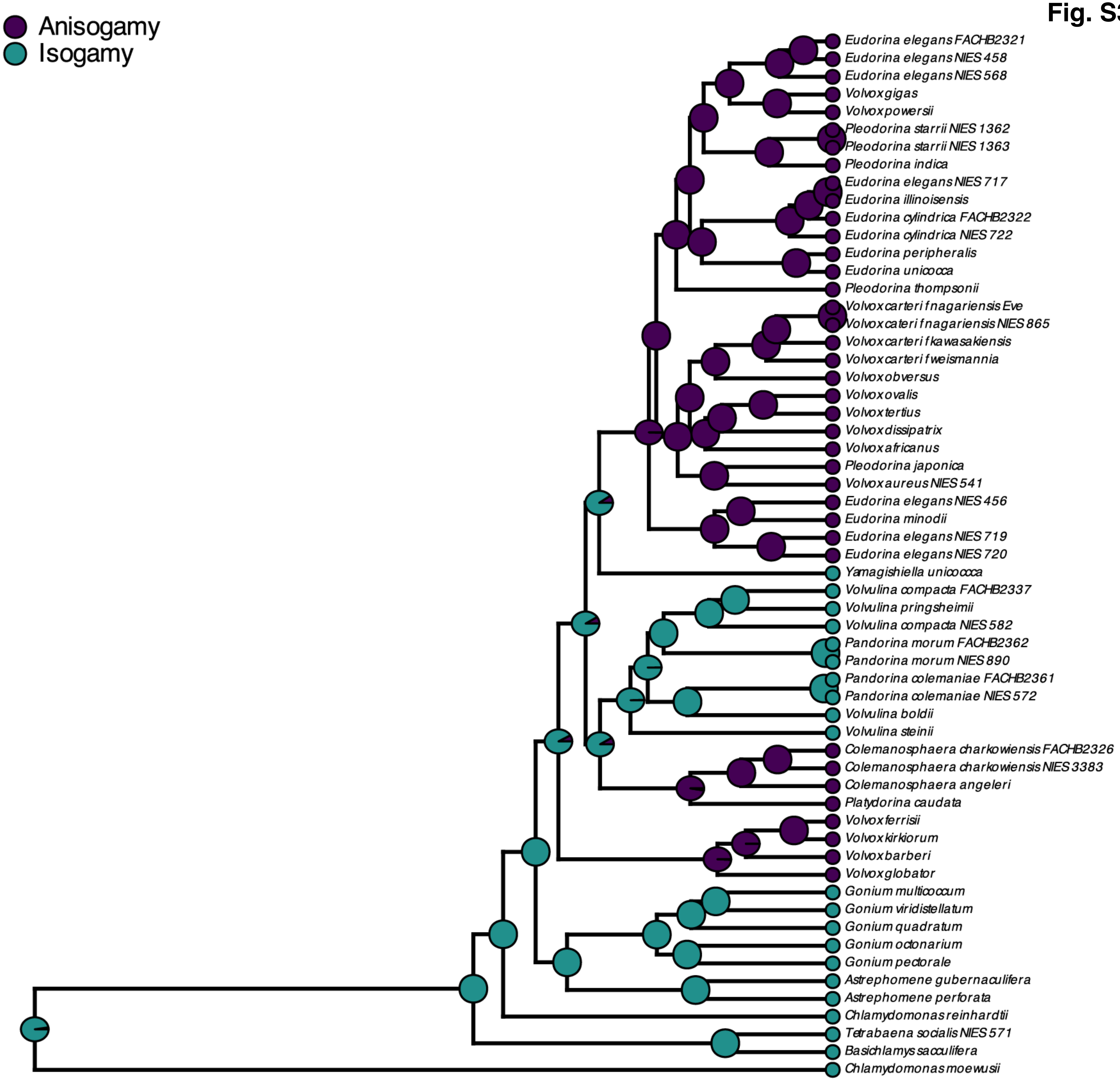

Anisogamy
Isogamy
Eudorina elegans FACHB2321
Eudorina elegans NIES 458
Eudorina elegans NIES 568
Volvox gigas
Volvox powersii
Pleodorina starrii NIES 1362
Pleodorina starrii NIES 1363
Pleodorina indica
Eudorina elegans NIES 717
Eudorina illinoisensis
Eudorina cylindrica FACHB2322
Eudorina cylindrica NIES 722
Eudorina peripheralis
Eudorina unicocca
Pleodorina thompsonii
Volvox carteri f nagariensis Eve
Volvox cateri f nagariensis NIES 865
Volvox carteri f kawasakiensis
Volvox carteri f weismannia
Volvox obversus
Volvox ovalis
Volvox tertius
Volvox dissipatrix
Volvox africanus
Pleodorina japonica
Volvox aureus NIES 541
Eudorina elegans NIES 456
Eudorina minodii
Eudorina elegans NIES 719
Eudorina elegans NIES 720
Yamagishiella unicocca
Volvulina compacta FACHB2337
Volvulina pringsheimii
Volvulina compacta NIES 582
Pandorina morum FACHB2362
Pandorina morum NIES 890
Pandorina colemaniae FACHB2361
Pandorina colemaniae NIES 572
Volvulina boldii
Volvulina steinii
Colemanosphaera charkowiensis FACHB2326
Colemanosphaera charkowiensis NIES 3383
Colemanosphaera angeleri
Platydorina caudata
Volvox ferrisii
Volvox kirkiorum
Volvox barberi
Volvox globator
Gonium multicoccum
Gonium viridistellatum
Gonium quadratum
Gonium octonarium
Gonium pectorale
Astrephomene gubernaculifera
Astrephomene perforata
Chlamydomonas reinhardtii
Tetrabaena socialis NIES 571
Basichlamys sacculifera
Chlamydomonas moewusii

Fig. S36

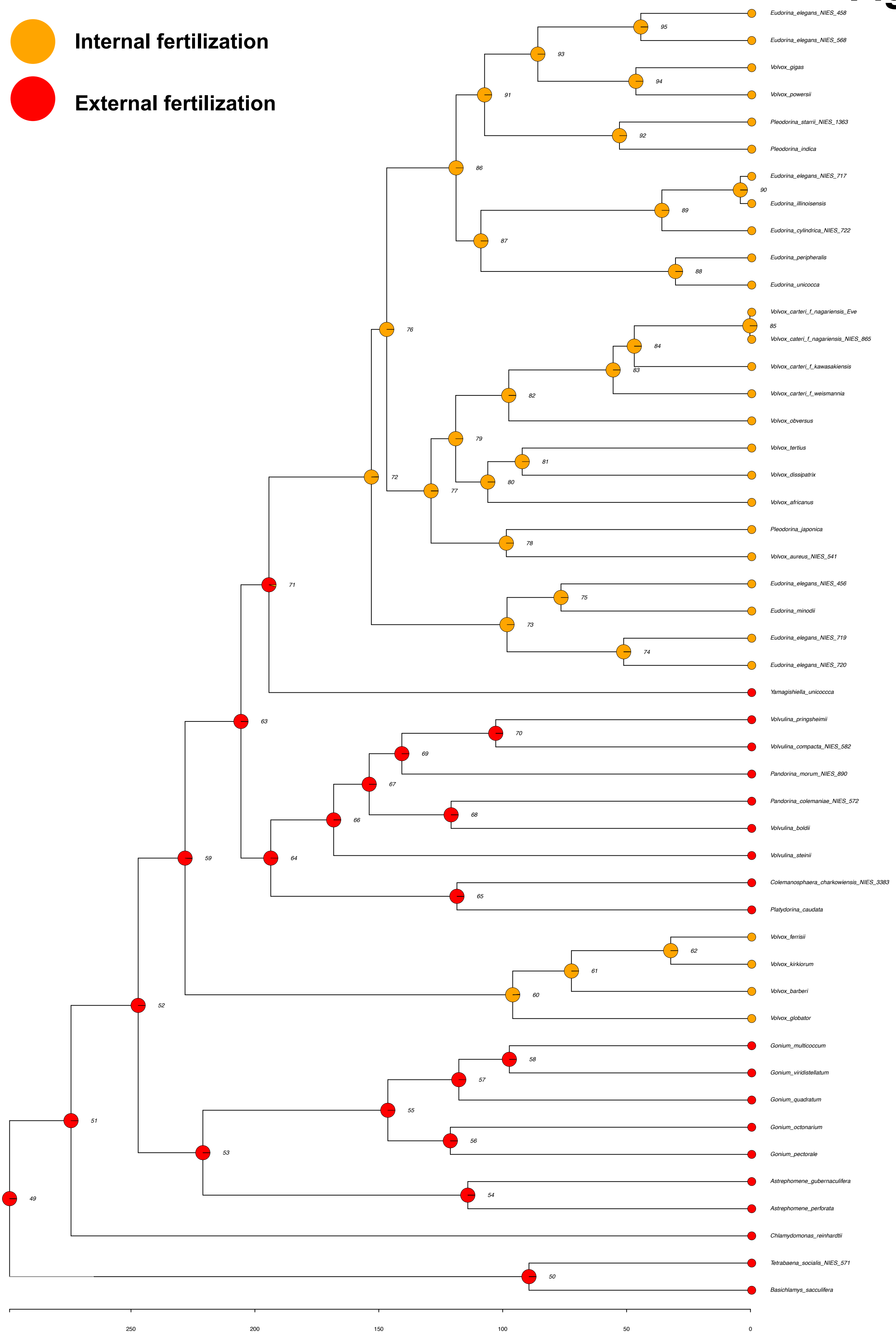

Internal fertilization
External fertilization
Eudorina_elegans_NIES_458
Eudorina_elegans_NIES_568
Volvox_gigas
Volvox_powersii
Pleodorina_starrii_NIES_1363
Pleodorina_indica
Eudorina_elegans_NIES_717
Eudorina_illinoisensis
Eudorina_cylindrica_NIES_722
Eudorina_peripheralis
Eudorina_unicocca
Volvox_carteri_f_nagariensis_Eve
Volvox_cateri_f_nagariensis_NIES_865
Volvox_carteri_f_kawasakiensis
Volvox_carteri_f_weismannia
Volvox_obversus
Volvox_tertius
Volvox_dissipatrix
Volvox_africanus
Pleodorina_japonica
Volvox_aureus_NIES_541
Eudorina_elegans_NIES_456
Eudorina_minodii
Eudorina_elegans_NIES_719
Eudorina_elegans_NIES_720
Yamagishiella_unicoccca
Volvulina_pringsheimii
Volvulina_compacta_NIES_582
Pandorina_morum_NIES_890
Pandorina_colemaniae_NIES_572
Volvulina_boldii
Volvulina_steinii
Colemanosphaera_charkowiensis_NIES_3383
Platydorina_caudata
Volvox_ferrisii
Volvox_kirkiorum
Volvox_barberi
Volvox_globator
Gonium_multicoccum
Gonium_viridistellatum
Gonium_quadratum
Gonium_octonarium
Gonium_pectorale
Astrephomene_gubernaculifera
Astrephomene_perforata
Chlamydomonas_reinhardtii
Tetrabaena_socialis_NIES_571
Basichlamys_sacculifera
250
200
150
100
50
0

Fig. S37

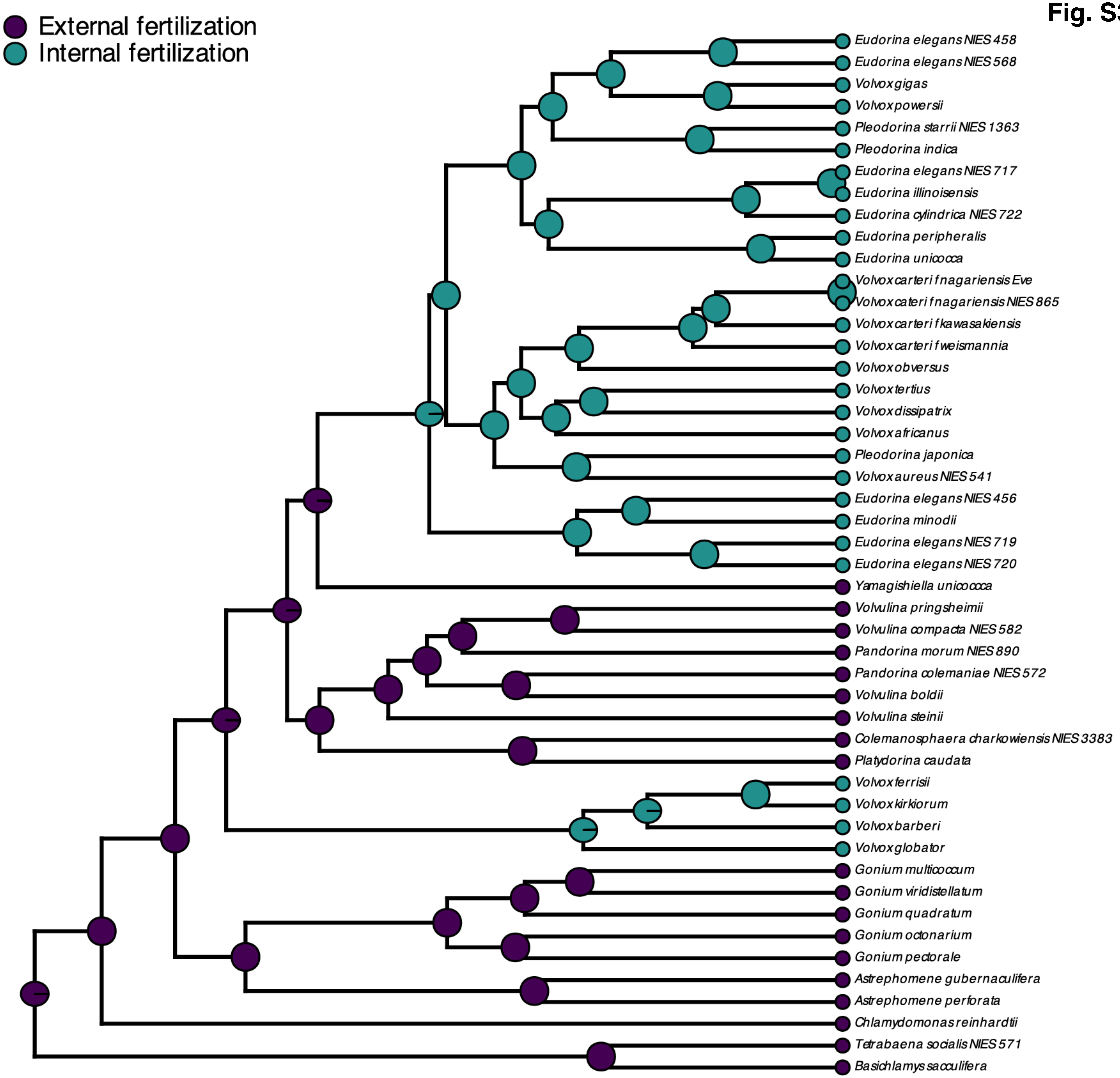

External fertilization
Internal fertilization
Eudorina elegans NIES 458
Eudorina elegans NIES 568
Volvox gigas
Volvox powersii
Pleodorina starrii NIES 1363
Pleodorina indica
Eudorina elegans NIES 717
Eudorina illinoisensis
Eudorina cylindrica NIES 722
Eudorina peripheralis
Eudorina unicocca
Volvox carteri f nagariensis Eve
Volvox cateri f nagariensis NIES 865
Volvox carteri f kawasakiensis
Volvox carteri f weismannia
Volvox obversus
Volvox tertius
Volvox dissipatrix
Volvox africanus
Pleodorina japonica
Volvox aureus NIES 541
Eudorina elegans NIES 456
Eudorina minodii
Eudorina elegans NIES 719
Eudorina elegans NIES 720
Yamagishiella unicocca
Volvulina pringsheimii
Volvulina compacta NIES 582
Pandorina morum NIES 890
Pandorina colemaniae NIES 572
Volvulina boldii
Volvulina steinii
Colemanosphaera charkowiensis NIES 3383
Platydorina caudata
Volvox ferrisii
Volvox kirkiorum
Volvox barberi
Volvox globator
Gonium multicoccum
Gonium viridistellatum
Gonium quadratum
Gonium octonarium
Gonium pectorale
Astrephomene gubernaculifera
Astrephomene perforata
Chlamydomonas reinhardtii
Tetrabaena socialis NIES 571
Basichlamys sacculifera

Fig. S38

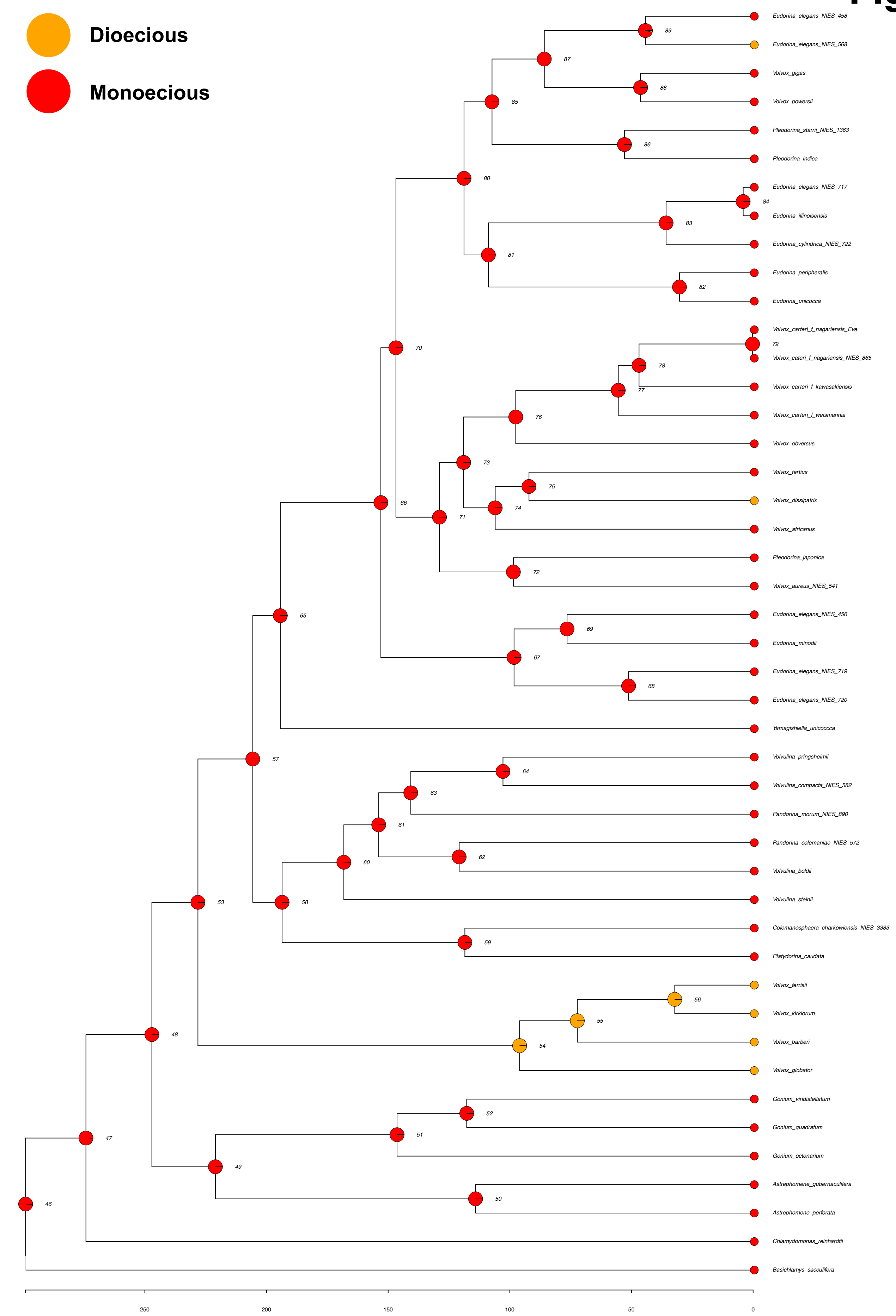

Dioecious
Monoecious
Eudorina_elegans_NIES_458
Eudorina_elegans_NIES_568
Volvox_gigas
Volvox_powersii
Pleodorina_starrii_NIES_1363
Pleodorina_indica
Eudorina_elegans_NIES_717
Eudorina_illinoisensis
Eudorina_cylindrica_NIES_722
Eudorina_peripheralis
Eudorina_unicocca
Volvox_carteri_f_nagariensis_Eve
Volvox_cateri_f_nagariensis_NIES_865
Volvox_carteri_f_kawasakiensis
Volvox_carteri_f_weismannia
Volvox_obversus
Volvox_tertius
Volvox_dissipatrix
Volvox_africanus
Pleodorina_japonica
Volvox_aureus_NIES_541
Eudorina_elegans_NIES_456
Eudorina_minodii
Eudorina_elegans_NIES_719
Eudorina_elegans_NIES_720
Yamagishiella_unicoccca
Volvulina_pringsheimii
Volvulina_compacta_NIES_582
Pandorina_morum_NIES_890
Pandorina_colemaniae_NIES_572
Volvulina_boldii
Volvulina_steinii
Colemanosphaera_charkowiensis_NIES_3383
Platydorina_caudata
Volvox_ferrisii
Volvox_kirkiorum
Volvox_barberi
Volvox_globator
Gonium_viridistellatum
Gonium_quadratum
Gonium_octonarium
Astrephomene_gubernaculifera
Astrephomene_perforata
Chlamydomonas_reinhardtii
Basichlamys_sacculifera
89
87
88
85
86
80
84
83
81
82
70
79
78
77
76
73
75
74
66
71
72
65
69
67
68
57
64
63
61
62
60
53
58
59
56
55
48
54
52
47
51
49
46
50
250
200
150
100
50
0

Fig. S39

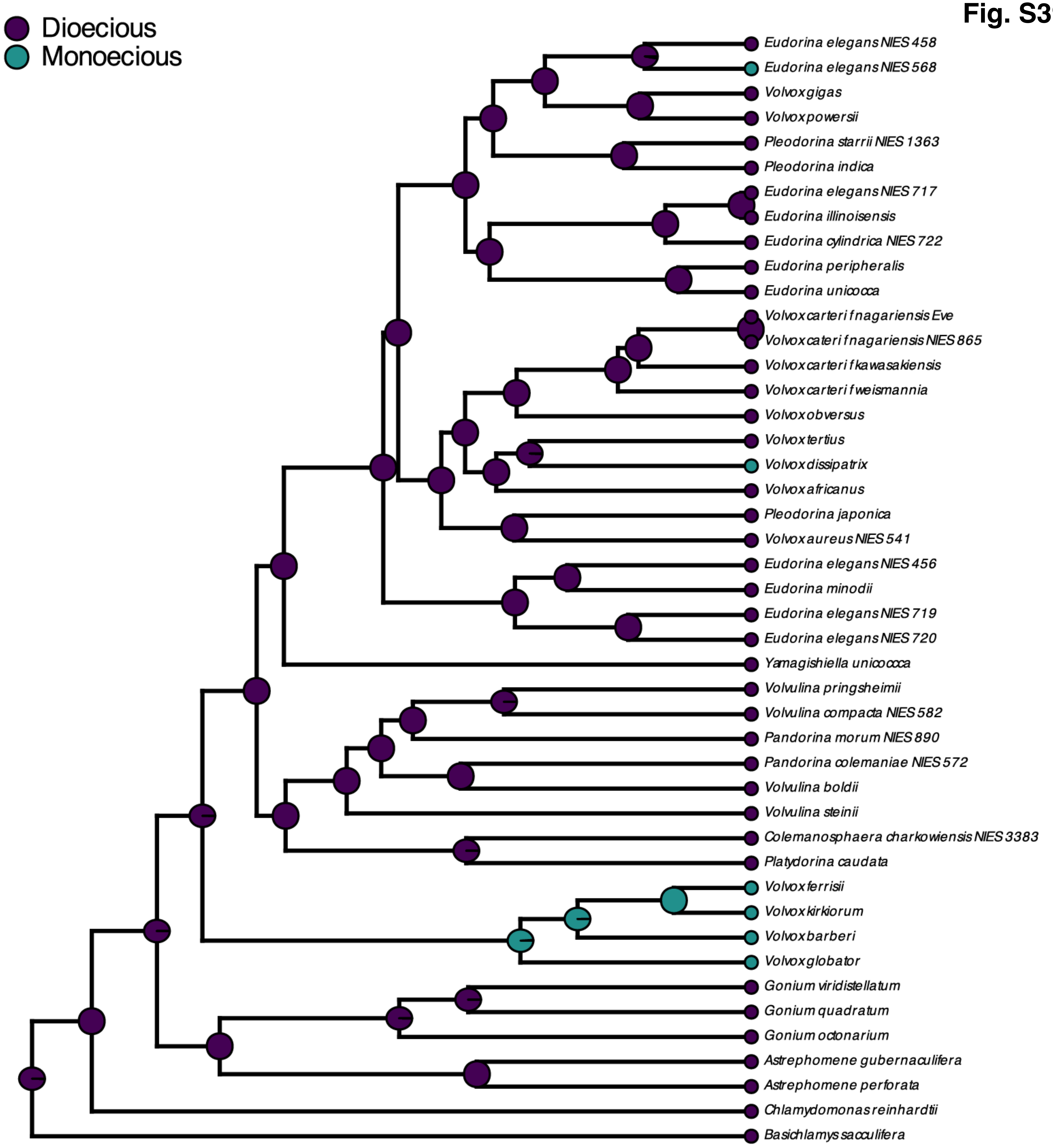
Dioecious
Monoecious
Eudorina elegans NIES 458
Eudorina elegans NIES 568
Volvox gigas
Volvox powersii
Pleodorina starrii NIES 1363
Pleodorina indica
Eudorina elegans NIES 717
Eudorina illinoisensis
Eudorina cylindrica NIES 722
Eudorina peripheralis
Eudorina unicocca
Volvox carteri f nagariensis Eve
Volvox cateri f nagariensis NIES 865
Volvox carteri f kawasakiensis
Volvox carteri f weismannia
Volvox obversus
Volvox tertius
Volvox dissipatrix
Volvox africanus
Pleodorina japonica
Volvox aureus NIES 541
Eudorina elegans NIES 456
Eudorina minodii
Eudorina elegans NIES 719
Eudorina elegans NIES 720
Yamagishiella unicocca
Volvulina pringsheimii
Volvulina compacta NIES 582
Pandorina morum NIES 890
Pandorina colemaniae NIES 572
Volvulina boldii
Volvulina steinii
Colemanosphaera charkowiensis NIES 3383
Platydorina caudata
Volvox ferrisii
Volvox kirkiorum
Volvox barberi
Volvox globator
Gonium viridistellatum
Gonium quadratum
Gonium octonarium
Astrephomene gubernaculifera
Astrephomene perforata
Chlamydomonas reinhardtii
Basichlamys sacculifera

Fig. S40

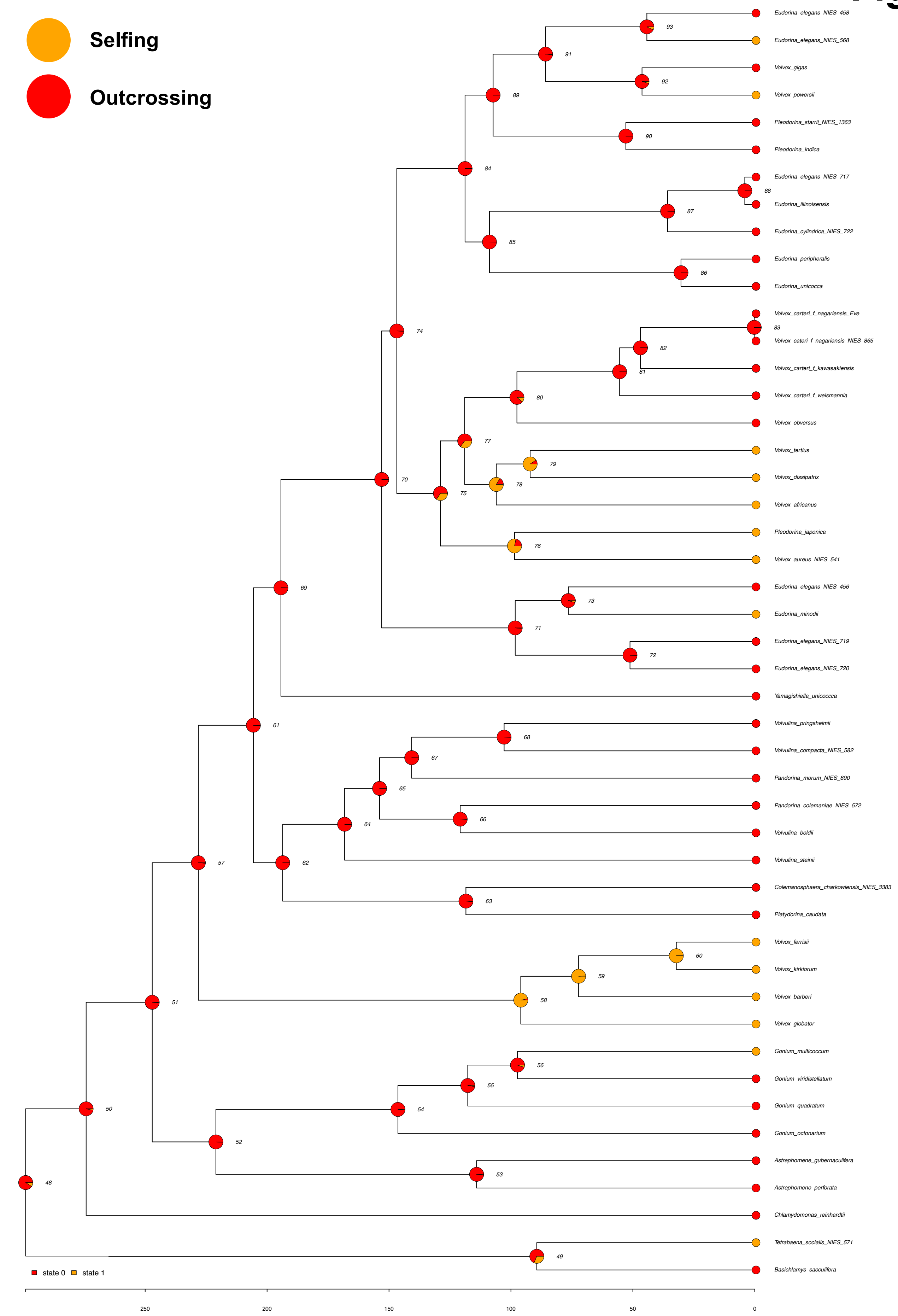

Selfing
Outcrossing
Eudorina_elegans_NIES_458
Eudorina_elegans_NIES_568
Volvox_gigas
Volvox_powersii
Pleodorina_starrii_NIES_1363
Pleodorina_indica
Eudorina_elegans_NIES_717
Eudorina_illinoisensis
Eudorina_cylindrica_NIES_722
Eudorina_peripheralis
Eudorina_unicocca
Volvox_carteri_f_nagariensis_Eve
Volvox_cateri_f_nagariensis_NIES_865
Volvox_carteri_f_kawasakiensis
Volvox_carteri_f_weismannia
Volvox_obversus
Volvox_tertius
Volvox_dissipatrix
Volvox_africanus
Pleodorina_japonica
Volvox_aureus_NIES_541
Eudorina_elegans_NIES_456
Eudorina_minodii
Eudorina_elegans_NIES_719
Eudorina_elegans_NIES_720
Yamagishiella_unicoccca
Volvulina_pringsheimii
Volvulina_compacta_NIES_582
Pandorina_morum_NIES_890
Pandorina_colemaniae_NIES_572
Volvulina_boldii
Volvulina_steinii
Colemanosphaera_charkowiensis_NIES_3383
Platydorina_caudata
Volvox_ferrisii
Volvox_kirkiorum
Volvox_barberi
Volvox_globator
Gonium_multicoccum
Gonium_viridistellatum
Gonium_quadratum
Gonium_octonarium
Astrephomene_gubernaculifera
Astrephomene_perforata
Chlamydomonas_reinhardtii
Tetrabaena_socialis_NIES_571
Basichlamys_sacculifera
93
92
91
90
89
88
87
86
85
84
83
82
81
80
77
79
78
75
74
70
76
73
71
72
69
68
67
65
66
64
61
62
63
57
60
59
58
51
56
55
54
50
52
53
48
49
state 0
state 1
250
200
150
100
50
0

Fig. S41

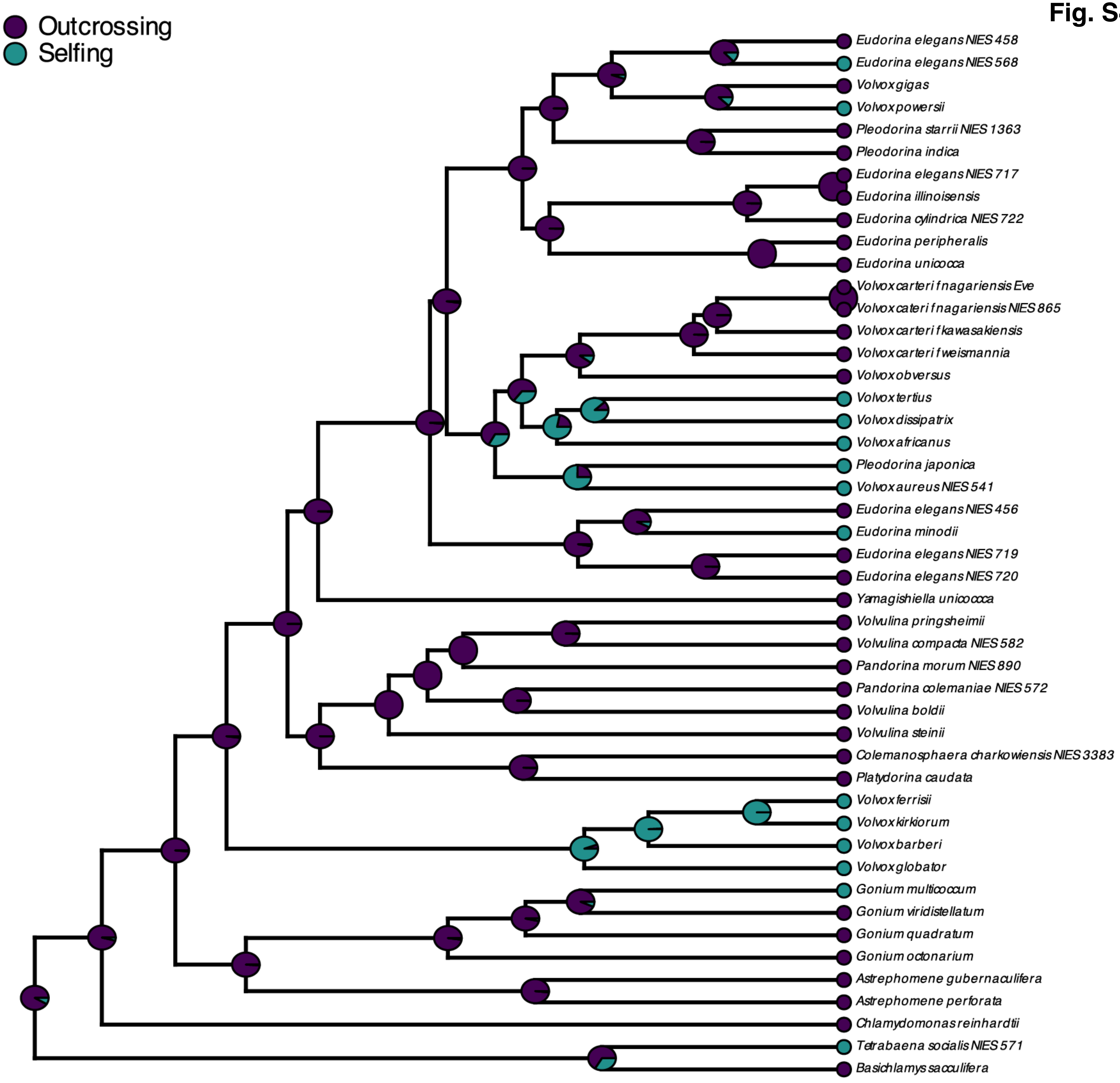
Outcrossing
Selfing
Eudorina elegans NIES 458
Eudorina elegans NIES 568
Volvox gigas
Volvox powersii
Pleodorina starrii NIES 1363
Pleodorina indica
Eudorina elegans NIES 717
Eudorina illinoisensis
Eudorina cylindrica NIES 722
Eudorina peripheralis
Eudorina unicocca
Volvox carteri f nagariensis Eve
Volvox cateri f nagariensis NIES 865
Volvox carteri f kawasakiensis
Volvox carteri f weismannia
Volvox obversus
Volvox tertius
Volvox dissipatrix
Volvox africanus
Pleodorina japonica
Volvox aureus NIES 541
Eudorina elegans NIES 456
Eudorina minodii
Eudorina elegans NIES 719
Eudorina elegans NIES 720
Yamagishiella unicocca
Volvulina pringsheimii
Volvulina compacta NIES 582
Pandorina morum NIES 890
Pandorina colemaniae NIES 572
Volvulina boldii
Volvulina steinii
Colemanosphaera charkowiensis NIES 3383
Platydorina caudata
Volvox ferrisii
Volvox kirkiorum
Volvox barberi
Volvox globator
Gonium multicoccum
Gonium viridistellatum
Gonium quadratum
Gonium octonarium
Astrephomene gubernaculifera
Astrephomene perforata
Chlamydomonas reinhardtii
Tetrabaena socialis NIES 571
Basichlamys sacculifera

Fig. S42

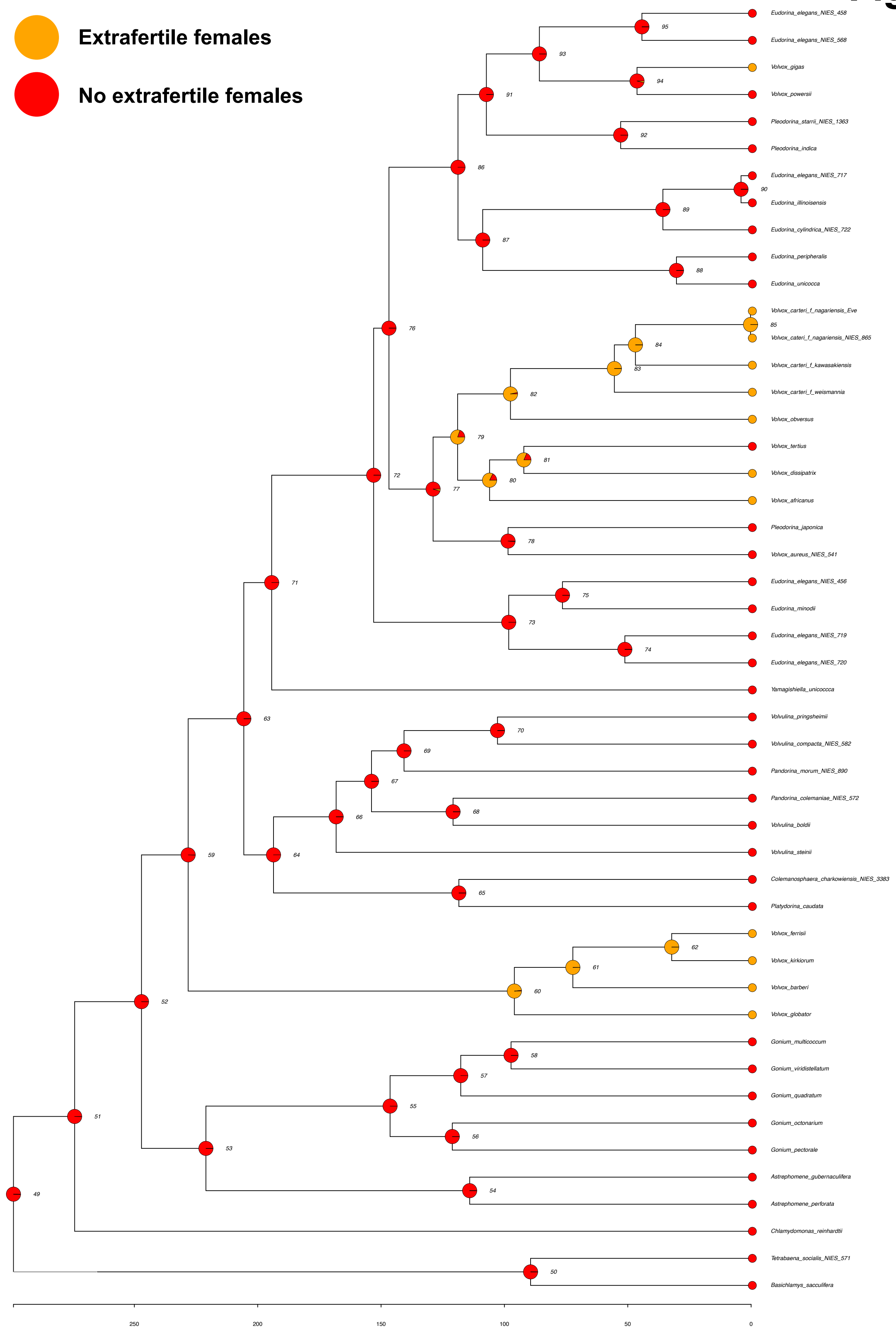

Extrafertile females
No extrafertile females
Eudorina_elegans_NIES_458
Eudorina_elegans_NIES_568
Volvox_gigas
Volvox_powersii
Pleodorina_starrii_NIES_1363
Pleodorina_indica
Eudorina_elegans_NIES_717
Eudorina_illinoisensis
Eudorina_cylindrica_NIES_722
Eudorina_peripheralis
Eudorina_unicocca
Volvox_carteri_f_nagariensis_Eve
Volvox_cateri_f_nagariensis_NIES_865
Volvox_carteri_f_kawasakiensis
Volvox_carteri_f_weismannia
Volvox_obversus
Volvox_tertius
Volvox_dissipatrix
Volvox_africanus
Pleodorina_japonica
Volvox_aureus_NIES_541
Eudorina_elegans_NIES_456
Eudorina_minodii
Eudorina_elegans_NIES_719
Eudorina_elegans_NIES_720
Yamagishiella_unicoccca
Volvulina_pringsheimii
Volvulina_compacta_NIES_582
Pandorina_morum_NIES_890
Pandorina_colemaniae_NIES_572
Volvulina_boldii
Volvulina_steinii
Colemanosphaera_charkowiensis_NIES_3383
Platydorina_caudata
Volvox_ferrisii
Volvox_kirkiorum
Volvox_barberi
Volvox_globator
Gonium_multicoccum
Gonium_viridistellatum
Gonium_quadratum
Gonium_octonarium
Gonium_pectorale
Astrephomene_gubernaculifera
Astrephomene_perforata
Chlamydomonas_reinhardtii
Tetrabaena_socialis_NIES_571
Basichlamys_sacculifera
250
200
150
100
50
0

Fig. S43

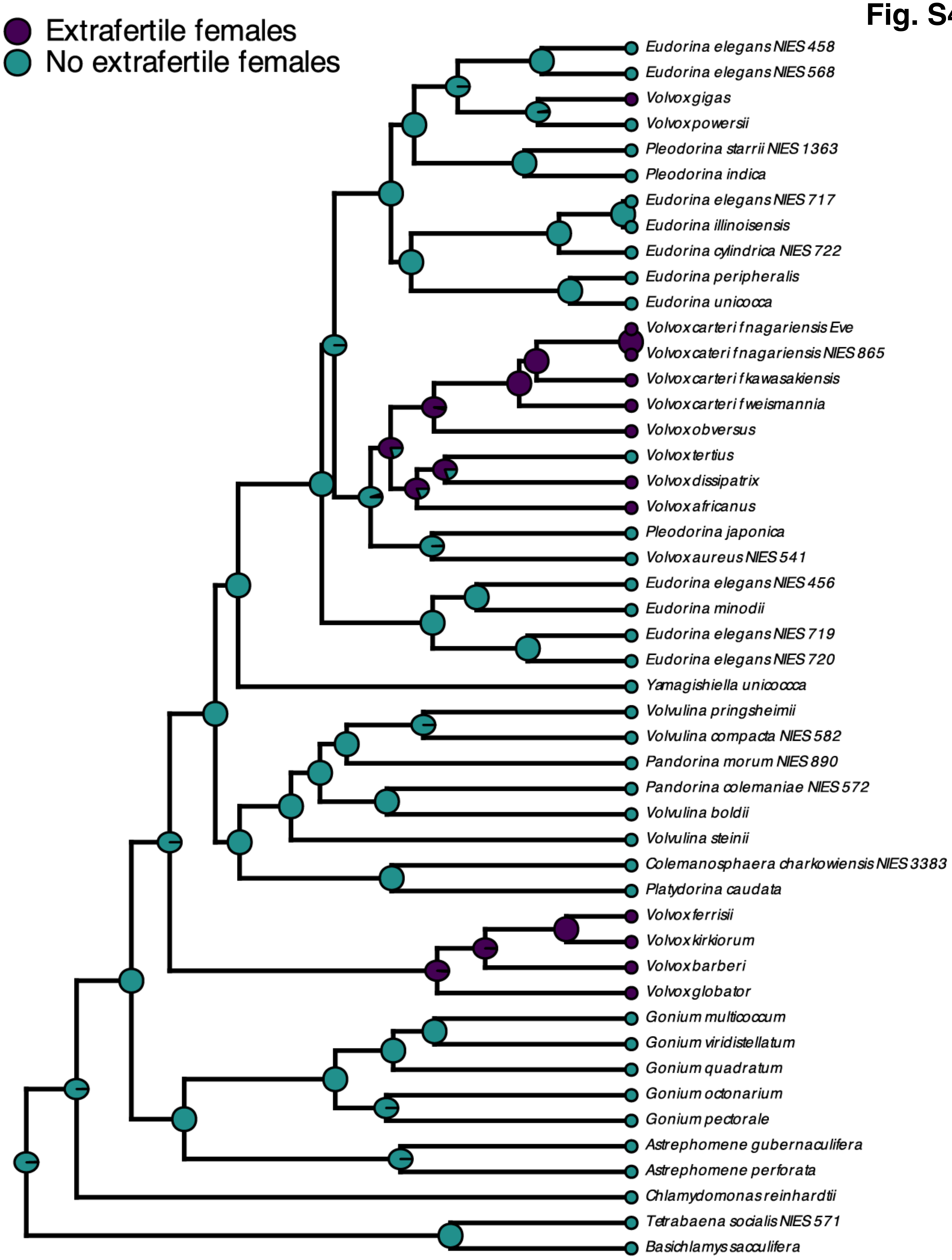

Extrafertile females
No extrafertile females
Eudorina elegans NIES 458
Eudorina elegans NIES 568
Volvox gigas
Volvox powersii
Pleodorina starrii NIES 1363
Pleodorina indica
Eudorina elegans NIES 717
Eudorina illinoisensis
Eudorina cylindrica NIES 722
Eudorina peripheralis
Eudorina unicocca
Volvox carteri f nagariensis Eve
Volvox cateri f nagariensis NIES 865
Volvox carteri f kawasakiensis
Volvox carteri f weismannia
Volvox obversus
Volvox tertius
Volvox dissipatrix
Volvox africanus
Pleodorina japonica
Volvox aureus NIES 541
Eudorina elegans NIES 456
Eudorina minodii
Eudorina elegans NIES 719
Eudorina elegans NIES 720
Yamagishiella unicocca
Volvulina pringsheimii
Volvulina compacta NIES 582
Pandorina morum NIES 890
Pandorina colemaniae NIES 572
Volvulina boldii
Volvulina steinii
Colemanosphaera charkowiensis NIES 3383
Platydorina caudata
Volvox ferrisii
Volvox kirkiorum
Volvox barberi
Volvox globator
Gonium multicoccum
Gonium viridistellatum
Gonium quadratum
Gonium octonarium
Gonium pectorale
Astrephomene gubernaculifera
Astrephomene perforata
Chlamydomonas reinhardtii
Tetrabaena socialis NIES 571
Basichlamys sacculifera

Fig. S44

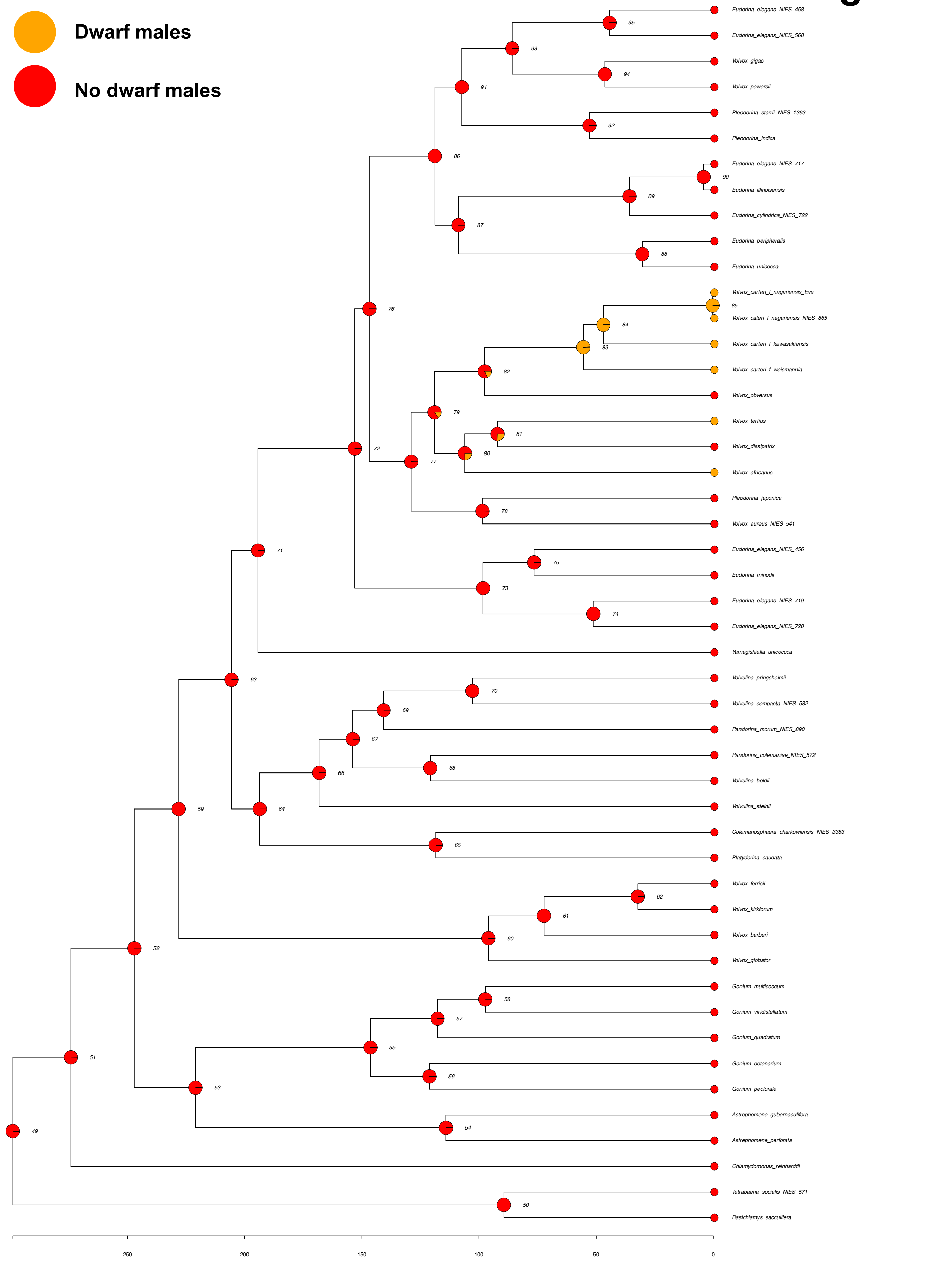
Dwarf males
No dwarf males
Eudorina_elegans_NIES_458
Eudorina_elegans_NIES_568
Volvox_gigas
Volvox_powersii
Pleodorina_starrii_NIES_1363
Pleodorina_indica
Eudorina_elegans_NIES_717
Eudorina_illinoisensis
Eudorina_cylindrica_NIES_722
Eudorina_peripheralis
Eudorina_unicocca
Volvox_carteri_f_nagariensis_Eve
Volvox_cateri_f_nagariensis_NIES_865
Volvox_carteri_f_kawasakiensis
Volvox_carteri_f_weismannia
Volvox_obversus
Volvox_tertius
Volvox_dissipatrix
Volvox_africanus
Pleodorina_japonica
Volvox_aureus_NIES_541
Eudorina_elegans_NIES_456
Eudorina_minodii
Eudorina_elegans_NIES_719
Eudorina_elegans_NIES_720
Yamagishiella_unicoccca
Volvulina_pringsheimii
Volvulina_compacta_NIES_582
Pandorina_morum_NIES_890
Pandorina_colemaniae_NIES_572
Volvulina_boldii
Volvulina_steinii
Colemanosphaera_charkowiensis_NIES_3383
Platydorina_caudata
Volvox_ferrisii
Volvox_kirkiorum
Volvox_barberi
Volvox_globator
Gonium_multicoccum
Gonium_viridistellatum
Gonium_quadratum
Gonium_octonarium
Gonium_pectorale
Astrephomene_gubernaculifera
Astrephomene_perforata
Chlamydomonas_reinhardtii
Tetrabaena_socialis_NIES_571
Basichlamys_sacculifera
250
200
150
100
50
0

Fig. S45

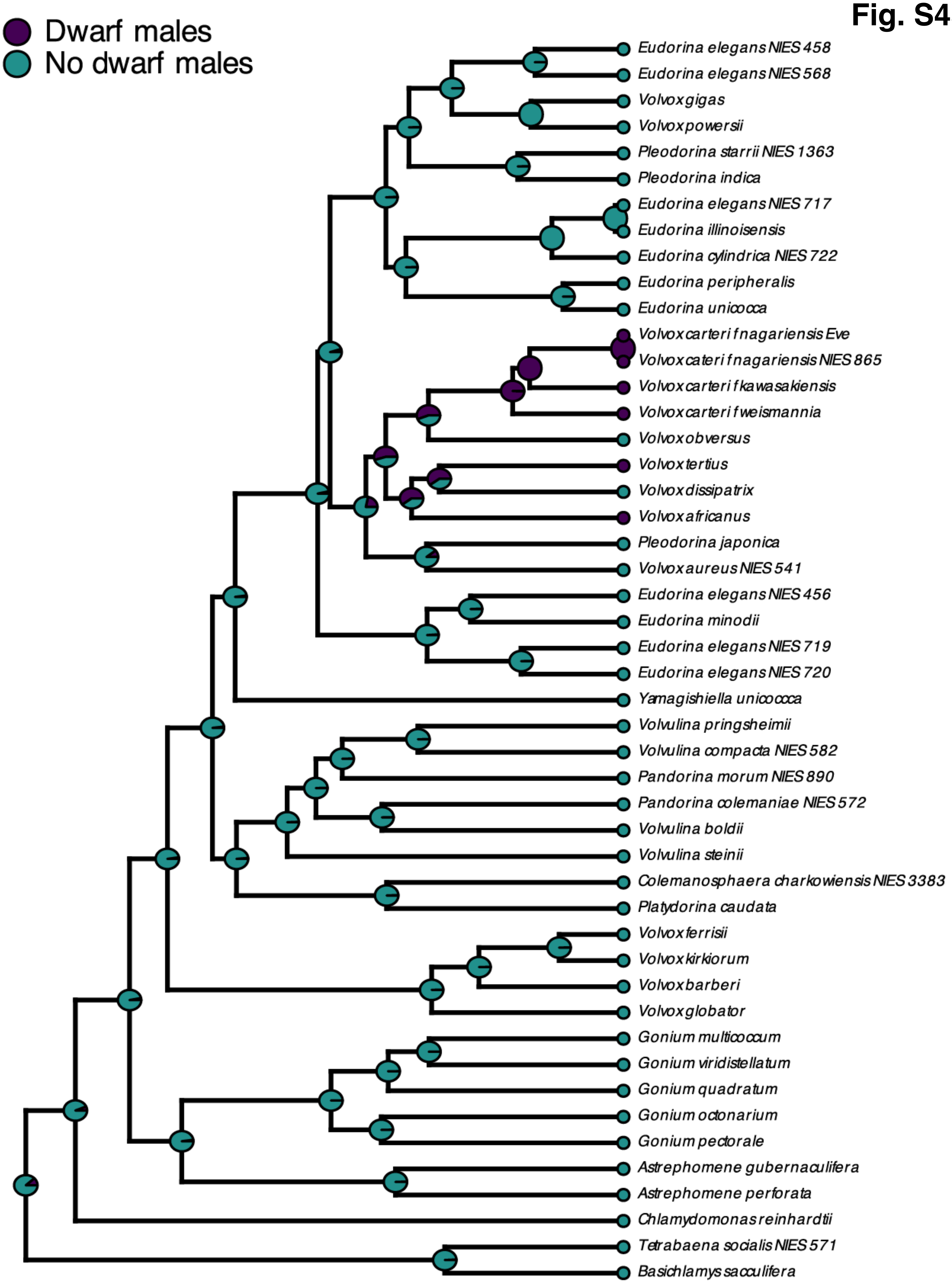
Dwarf males
No dwarf males
Eudorina elegans NIES 458
Eudorina elegans NIES 568
Volvox gigas
Volvox powersii
Pleodorina starrii NIES 1363
Pleodorina indica
Eudorina elegans NIES 717
Eudorina illinoisensis
Eudorina cylindrica NIES 722
Eudorina peripheralis
Eudorina unicocca
Volvox carteri f nagariensis Eve
Volvox cateri f nagariensis NIES 865
Volvox carteri f kawasakiensis
Volvox carteri f weismannia
Volvox obversus
Volvox tertius
Volvox dissipatrix
Volvox africanus
Pleodorina japonica
Volvox aureus NIES 541
Eudorina elegans NIES 456
Eudorina minodii
Eudorina elegans NIES 719
Eudorina elegans NIES 720
Yamagishiella unicocca
Volvulina pringsheimii
Volvulina compacta NIES 582
Pandorina morum NIES 890
Pandorina colemaniae NIES 572
Volvulina boldii
Volvulina steinii
Colemanosphaera charkowiensis NIES 3383
Platydorina caudata
Volvox ferrisii
Volvox kirkiorum
Volvox barberi
Volvox globator
Gonium multicoccum
Gonium viridistellatum
Gonium quadratum
Gonium octonarium
Gonium pectorale
Astrephomene gubernaculifera
Astrephomene perforata
Chlamydomonas reinhardtii
Tetrabaena socialis NIES 571
Basichlamys sacculifera

Fig. S46

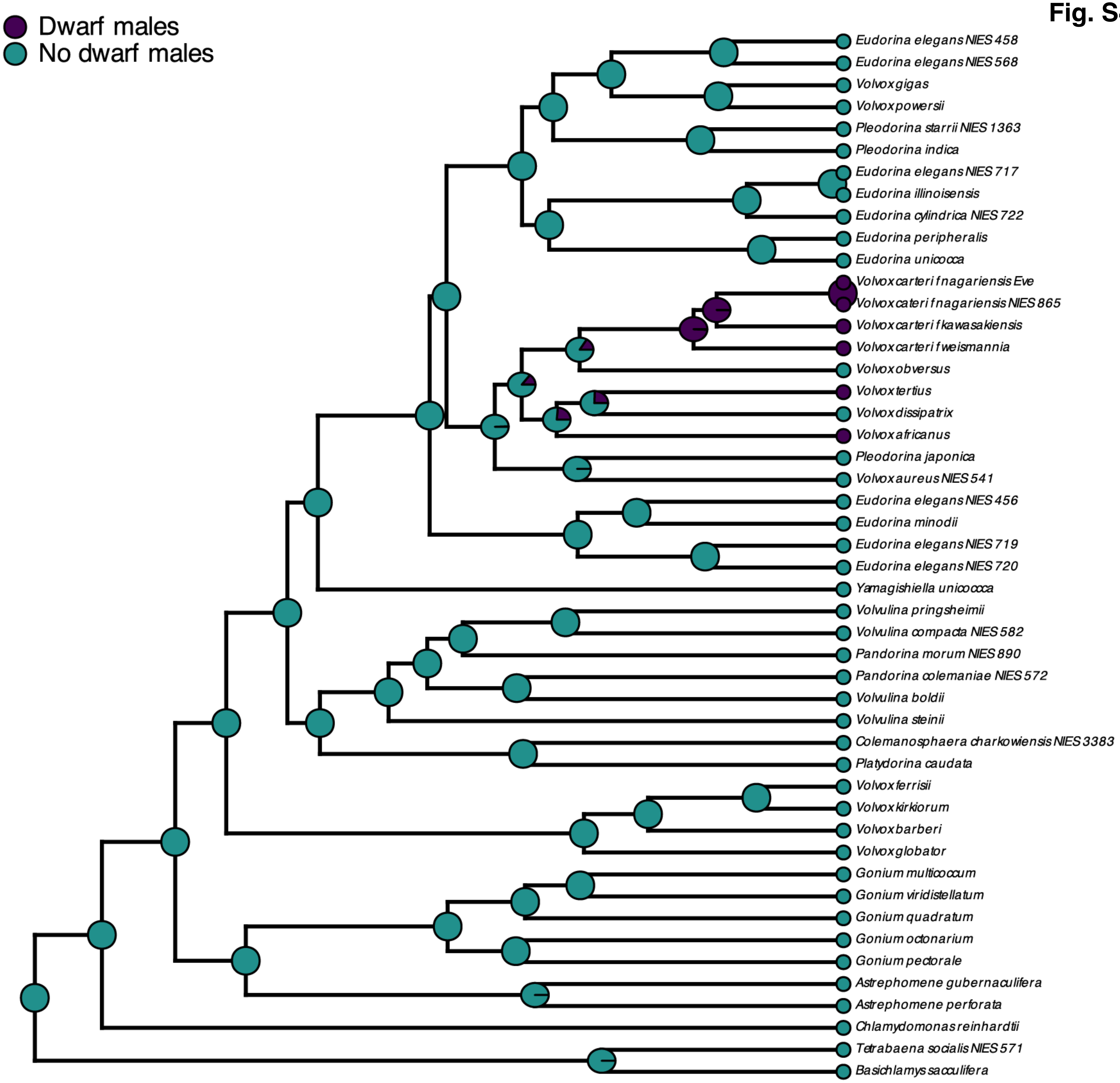

Dwarf males
No dwarf males
Eudorina elegans NIES 458
Eudorina elegans NIES 568
Volvox gigas
Volvox powersii
Pleodorina starrii NIES 1363
Pleodorina indica
Eudorina elegans NIES 717
Eudorina illinoisensis
Eudorina cylindrica NIES 722
Eudorina peripheralis
Eudorina unicocca
Volvox carteri f nagariensis Eve
Volvox cateri f nagariensis NIES 865
Volvox carteri f kawasakiensis
Volvox carteri f weismannia
Volvox obversus
Volvox tertius
Volvox dissipatrix
Volvox africanus
Pleodorina japonica
Volvox aureus NIES 541
Eudorina elegans NIES 456
Eudorina minodii
Eudorina elegans NIES 719
Eudorina elegans NIES 720
Yamagishiella unicocca
Volvulina pringsheimii
Volvulina compacta NIES 582
Pandorina morum NIES 890
Pandorina colemaniae NIES 572
Volvulina boldii
Volvulina steinii
Colemanosphaera charkowiensis NIES 3383
Platydorina caudata
Volvox ferrisii
Volvox kirkiorum
Volvox barberi
Volvox globator
Gonium multicoccum
Gonium viridistellatum
Gonium quadratum
Gonium octonarium
Gonium pectorale
Astrephomene gubernaculifera
Astrephomene perforata
Chlamydomonas reinhardtii
Tetrabaena socialis NIES 571
Basichlamys sacculifera
